# GCN5-mediated regulation of pathological cardiac hypertrophy via activation of the TAK1-JNK/p38 signaling pathway

**DOI:** 10.1038/s41419-022-04881-y

**Published:** 2022-04-30

**Authors:** Jia Li, Chenghui Yan, Yilong Wang, Can Chen, Haibo Yu, Dan Liu, Kai Huang, Yaling Han

**Affiliations:** 1 Department of Cardiology, Union Hospital, Tongji Medical College, Huazhong University of Science and Technology, Wuhan, China; 2 Clinic Center of Human Gene Research, Union Hospital, Tongji Medical College, Huazhong University of Science and Technology, Wuhan, China; 3 Cardiovascular Research Institute and Department of Cardiology, General Hospital of Northern Theater Command, Shenyang, China; 4 Department of Cardiology, The First Affiliated Hospital of Sun Yat-Sen University, Guangzhou, China

**Keywords:** Heart failure, Cell signalling

## Abstract

Pathological cardiac hypertrophy is a process of abnormal remodeling of cardiomyocytes in response to pressure overload or other stress stimuli, resulting in myocardial injury, which is a major risk factor for heart failure, leading to increased morbidity and mortality. General control nonrepressed protein 5 (GCN5)/lysine acetyltransferase 2 A, a member of the histone acetyltransferase and lysine acetyltransferase families, regulates a variety of physiological and pathological events. However, the function of GCN5 in pathological cardiac hypertrophy remains unclear. This study aimed to explore the role of GCN5 in the development of pathological cardiac hypertrophy. GCN5 expression was increased in isolated neonatal rat cardiomyocytes (NRCMs) and mouse hearts of a hypertrophic mouse model. GCN5 overexpression aggravated the cardiac hypertrophy triggered by transverse aortic constriction surgery. In contrast, inhibition of GCN5 impairs the development of pathological cardiac hypertrophy. Similar results were obtained upon stimulation of NRCMs (having GCN5 overexpressed or knocked down) with phenylephrine. Mechanistically, our results indicate that GCN5 exacerbates cardiac hypertrophy via excessive activation of the transforming growth factor β-activated kinase 1 (TAK1)-c-Jun N-terminal kinase (JNK)/p38 signaling pathway. Using a TAK1-specific inhibitor in rescue experiments confirmed that the activation of TAK1 is essential for GCN5-mediated cardiac hypertrophy. In summary, the current study elucidated the role of GCN5 in promotion of cardiac hypertrophy, thereby implying it to be a potential target for treatment.

## Introduction

Cardiovascular disease, which is the leading cause of death worldwide, has become a global challenge [1]. Cardiac hypertrophy, which is characterized by the enlargement of cardiomyocytes and interstitial fibrosis, is the primary response of the heart to mechanical stress induced by extrinsic factors, including increased pressure or volume overload in hypertension and valvular diseases, or intrinsic factors such as ischemia-induced cardiac remodeling and expansive cardiomyopathy [2, 3]. The complex process of cardiac hypertrophy involves abnormal protein synthesis, excessive activation of multiple factors and pathways, accumulation of extracellular matrix, and fetal cardiac gene re-expression, leading to cardiac dysfunction and eventually heart failure [4].

Cardiac hypertrophic stimuli lead to excessive activation of intricate but coordinated signaling pathways, such as the mitogen-activated protein kinase (MAPK), protein kinase C, phosphoinositide-3-kinase–protein kinase B/Akt (PI3K-PKB/Akt), and calcineurin/nuclear factor of activated T cell pathways [5–7]. These are significant signaling pathways involved in the development of cardiac hypertrophy. The MAPK signaling cascade, which consists of a three-tiered kinase cascade (MAP3K-MAP2K-MAPK), plays a prominent role in mediating cardiac hypertrophy [8]. As a pivotal member of the MAP3K family, transforming growth factor beta (TGF-β)-activated kinase 1 (TAK1 or MAP3K7) can be activated by TGF-β, bone morphogenetic protein, and other cytokines, and it is tightly regulated through its binding partners and via protein modifications [9]. In vivo activation of TAK1 requires association of TAK1 with TAK1-binding protein 1 (TAB1), which triggers the phosphorylation of TAK1 [10, 11]. Once activated, TAK1 regulates the activation of c-Jun N-terminal kinase (JNK) and p38 MAPKs [10, 12]. Therefore, regulation of TAK1 activation may serve as an potential target for preventing cardiac hypertrophy.

General control nonrepressed protein 5 (GCN5)/lysine acetyltransferase 2 A (KAT2A) acts as a histone acetyltransferase and a lysine acetyltransferase, and it is involved in multiple cellular processes, such as DNA repair [13], gene transcription [14], differentiation [15], cell cycle regulation [16], and nucleosome assembly [17] through histone acetylation, histone succinylation, and recruitment of transcriptional co-activators [18, 19]. In addition, GCN5 acetylates other non-histone substrates, including T-box transcription factor 5 [20], androgen receptor [21], polo-like kinase 4 [22], and CCAAT enhancer-binding protein beta [15]. However, the role of GCN5 in the development of cardiac hypertrophy remains poorly understood.

In the present study, we examined the expression of GCN5 in an animal model of cardiac hypertrophy induced by transverse aortic constriction (TAC) and in cultured neonatal rat cardiomyocytes (NRCMs) stimulated with phenylephrine (PE). We then manipulated GCN5 levels via adeno-associated virus 9 in vivo and adenovirus in vitro to investigate the effect of GCN5 on cardiac hypertrophy. Furthermore, we blocked the TAK1-JNK/p38 signaling pathway with a TAK1 inhibitor (iTAK1), owing to which the effects of GCN5 overexpression were inhibited in both TAC- and PE-induced cardiac hypertrophy models. Therefore, this study aimed to explore the role of GCN5 in pathological cardiac hypertrophy.

## Results

### GCN5 expression is upregulated in hypertrophic hearts and cardiomyocytes

**Fig. 1:**
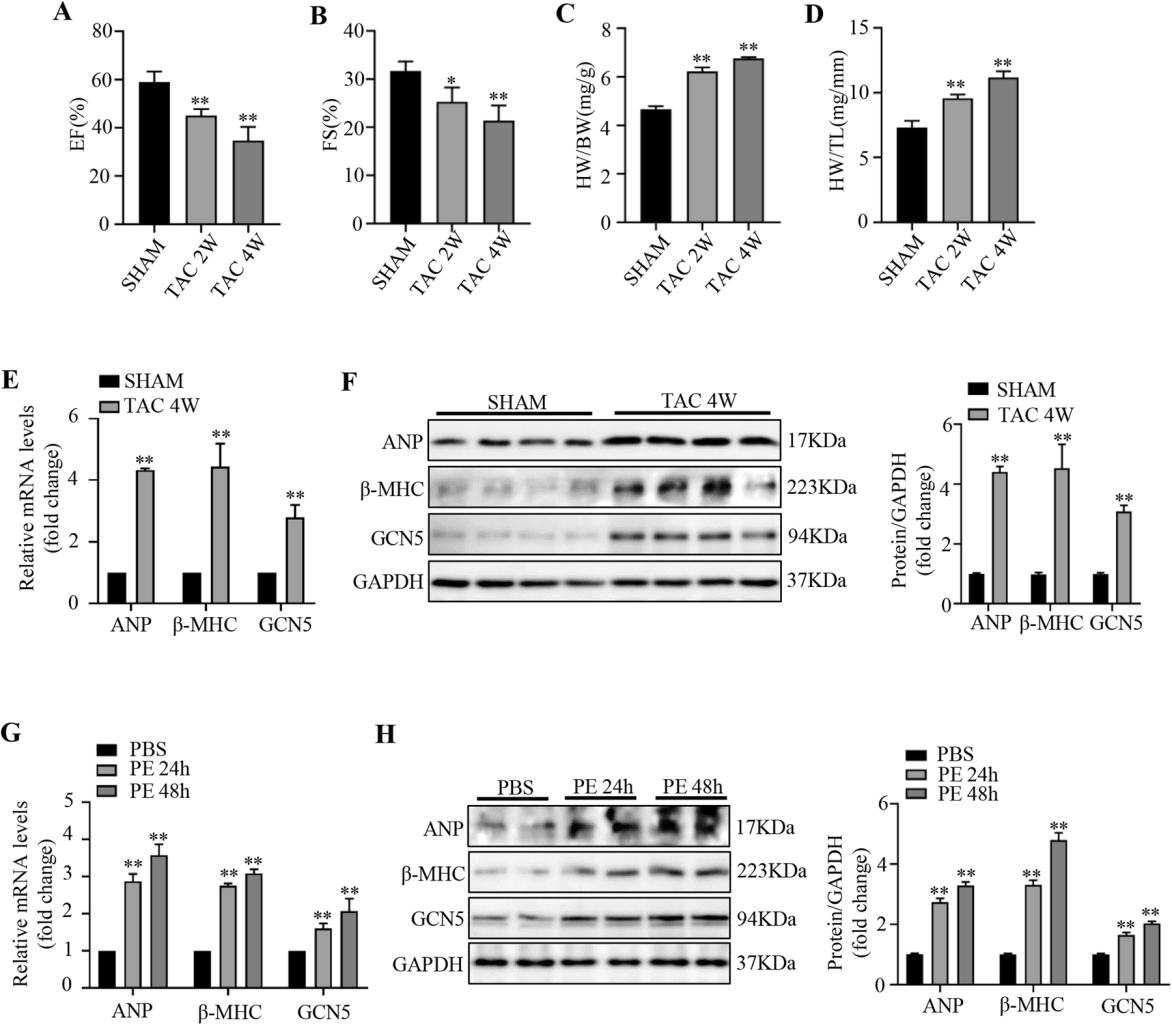
GCN5 expression is upregulated in hypertrophic mouse hearts and cardiomyocytes. **A**–**D** EF values, FS values, HW/BW ratios, and HW/TL ratios in mice subjected to sham or TAC surgery at 2 or 4 weeks. **E** ANP, β-MHC, and GCN5 mRNA levels in the hearts of mice subjected to sham or TAC surgery. **F** ANP, β-MHC, and GCN5 protein levels in the hearts of mice subjected to sham or TAC surgery. (*n* = 4 mice/group, ^∗^*P* < 0.05 vs SHAM, ^∗∗^*P* < 0.01 vs SHAM) (**G**) ANP, β-MHC, and GCN5 mRNA levels in NRCMs treated with PBS or PE (50 μM). **H** ANP, β-MHC, and GCN5 protein levels in NRCMs treated with PBS or PE (^∗∗^*P* < 0.01 vs PBS). Data are shown as the mean ± SD from three independent experiments.

To explore the potential effect of GCN5 on cardiac hypertrophy, we established a model of pathological cardiac hypertrophy via TAC surgery; we detected cardiac function using transthoracic echocardiography (Fig. 1A–D) and investigated the remodeling of histology (Supplementary Fig. 1A-B) at 2 and 4 weeks after sham or TAC surgery. Compared with the hearts of sham-operated mice, the hearts of mice subjected to TAC for 4 weeks exhibited higher expression of GCN5, as evidenced by immunohistochemical staining (Supplementary Fig. 1C). Furthermore, there was a noticeable increase in the mRNA and protein levels of GCN5 and cardiac hypertrophic markers, such as atrial natriuretic peptide (ANP) and β-myosin heavy chain (β-MHC) (Fig. 1E-F). PE was used to stimulate NRCMs to prepare a cardiac hypertrophic model in vitro. We analyzed the cell surface of NRCMs after PE stimulation (Supplementary Fig. 1D) and found that NRCMs treated with PE (50 μM) for 24 h and 48 h also showed an elevated expression of GCN5 and cardiac hypertrophic markers, both at the mRNA and protein levels (Fig. 1G-H). Collectively, these findings show that GCN5 expression is elevated in cardiac hypertrophy.

### GCN5 overexpression exacerbates pressure overload-induced cardiac hypertrophy in vivo

**Fig. 2:**
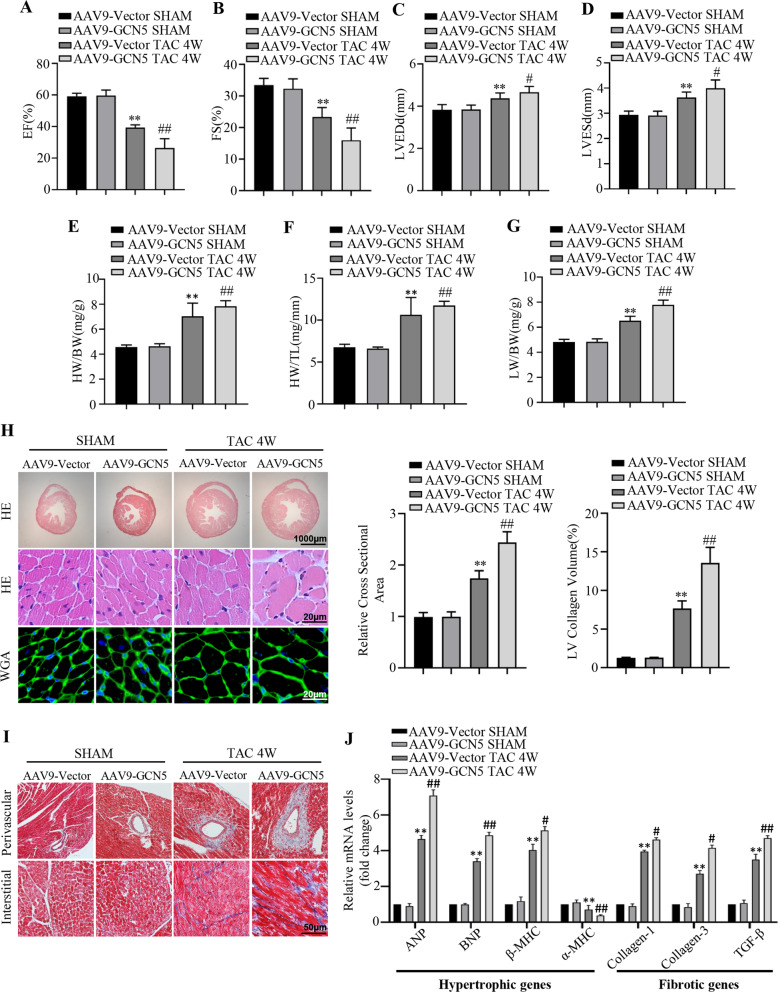
GCN5 overexpression accelerates pressure overload-induced cardiac hypertrophy and dysfunction. **A**–**D** Echocardiographic assessment of EF, FS, LVEDd, and LVESd values in GCN5-overexpressing mice and control mice subjected to sham or TAC surgery at 4 weeks (*n* = 10–12 mice/group). **E**–**G** HW/BW, HW/TL, and LW/BW ratios in GCN5-overexpressing mice and control mice subjected to sham or TAC surgery at 4 weeks (*n* = 10–12 mice/group). **H** Representative images of heart sections stained with H&E and WGA to analyze heart and cardiomyocyte size, respectively (*n* = 5 mice/group). **I** Representative images of heart sections stained with Masson’s trichrome stain to analyze perivascular and interstitial fibrotic area (*n* = 5 mice/group). **J** Effects of GCN5 overexpression on the mRNA levels of the hypertrophic marker genes and fibrotic marker genes in the hearts of mice subjected to sham or TAC surgery at 4 weeks (*n* = 5–7 mice/group, ^∗∗^*P* < 0.01 vs SHAM, ^#^*P* < 0.05 vs AAV9-Vector TAC 4 W, ^##^*P* < 0.01 vs AAV9-Vector TAC 4 W). Data are shown as the mean ± SD from three independent experiments.

To investigate the role of GCN5 in pressure overload-induced cardiac hypertrophy in vivo, GCN5 was overexpressed in mice using the AAV9 system containing null and GCN5 via tail vein injection. Under TAC treatment, GCN5 overexpression led to worse cardiac function than the control group (Fig. 2A–D), as well as by the increase in heart weight (HW)/body weight (BW), HW/tibia length (TL), and lung weight (LW)/BW (Fig. 2E–G). However, under basal condition, overexpressed GCN5 did not exhibit any heart differences compared with control group which was evidenced by similar ejection fraction (EF), fractional shortening (FS) values, left ventricular end-diastolic diameter (LVEDd) and left ventricular end-systolic diameter (LVESd) values. Cardiac remodeling is the main characteristic of cardiac hypertrophy, and enlargement of cardiomyocytes and interstitial fibrosis are acknowledged manifestations of this process. Histological examination revealed significantly greater cardiomyocyte cross-sectional areas in GCN5-overexpressing mice than in control mice after TAC surgery (Fig. 2H). Masson’s trichrome staining revealed that the collagen content in both the perivascular and interstitial spaces in GCN5-overexpressing mice was higher than that in control mice after TAC surgery (Fig. 2I). In addition, the mRNA levels of cardiac hypertrophy markers (ANP, BNP, α-MHC, and β-MHC) and fibrotic markers (collagen I, collagen III, and TGF-β) in GCN5-overexpressed hearts were markedly higher than those in control hearts (Fig. 2J).

As cardiomyocyte apoptosis is associated with the development of cardiac hypertrophy [23], we examined whether GCN5 impacts cardiomyocyte apoptosis during this process; levels of several apoptosis-associated proteins were quantified, and GCN5 overexpression significantly promoted apoptosis, as evidenced by increased pro-apoptotic protein levels and downregulated anti-apoptotic protein expression. GCN5 overexpression promoted apoptosis following hypertrophic stimulation (Supplementary Fig. 2A). These data demonstrate that GCN5 overexpression promotes cardiac hypertrophy and the cardiomyocyte apoptosis associated with it in vivo.

### GCN5 overexpression accelerates PE-induced cardiomyocyte hypertrophy

**Fig. 3:**
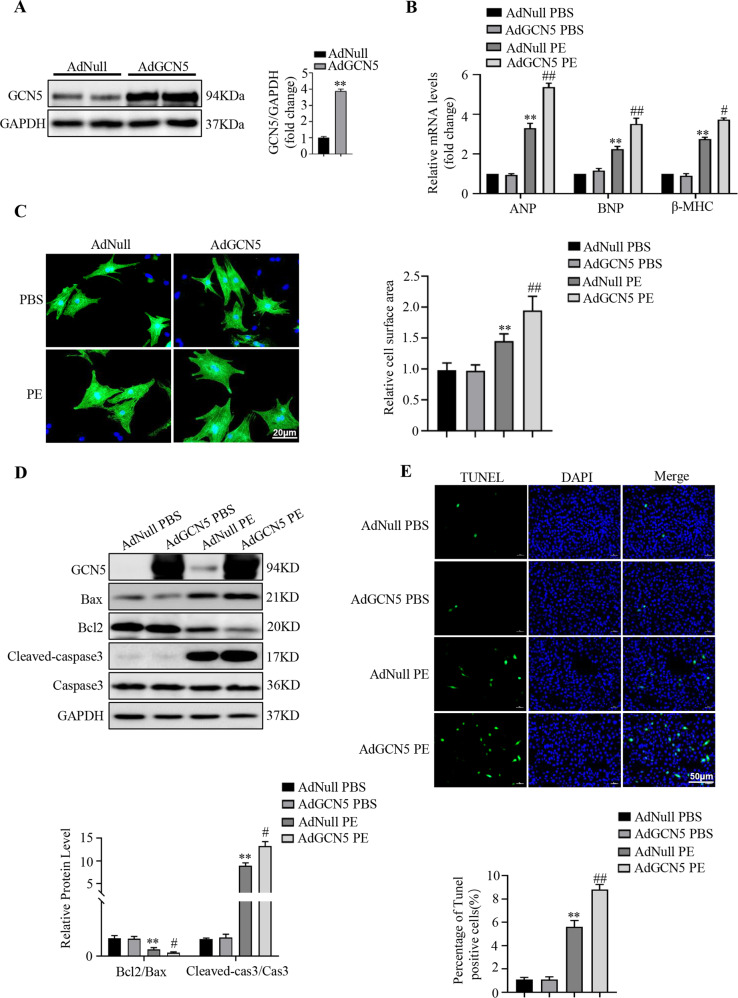
GCN5 overexpression accelerates PE-induced cardiomyocyte hypertrophy and apoptosis. **A** Immunoblotting of primary cardiomyocytes infected with AdNull or AdGCN5 for 24 h to indicate GCN5 expression levels. **B** Effects of GCN5 overexpression on the mRNA levels of hypertrophic marker genes. **C** Effects of GCN5 overexpression on cardiomyocyte surface areas. **D** Western blots showing that overexpressed GCN5 exacerbates PE-induced NRCM apoptosis. **E** Representative images of TUNEL staining, indicating apoptotic rates of NRCMs (^∗∗^*P* < 0.01 vs AdNull PBS; ^#^*P* < 0.05 vs AdNull PE, ^##^*P* < 0.01 vs AdNull PE). Data are shown as the mean ± SD from three independent experiments.

To further explore the effect of GCN5 on cardiac hypertrophy, we used PE to stimulate NRCMs, to establish a cardiomyocyte hypertrophy model in vitro. NRCMs were infected with adenoviruses containing sequences of GCN5 (AdGCN5) for GCN5 overexpression and adenoviruses containing no transgene expression cassette (AdNull) as a control (Fig. 3A). Then, we stimulated the NRCMs with PE or phosphate-buffered saline (PBS) for subsequent experiments. The infected cells were collected for analysis of fetal cardiac gene expression and immunofluorescence staining with α-actinin antibody to determine cell size. Under treatment with PE, cardiomyocytes infected with AdGCN5 exhibited higher expressions of cardiac hypertrophy markers at the mRNA and protein levels (Fig. 3B, Supplementary Fig. 2B), resulting in greater cell surface areas than cardiomyocytes infected with AdNull (Fig. 3C). However, there was no difference of expressions of cardiac hypertrophy markers and cell surface areas between control and GCN5 overexpressed groups in basal condition.

In addition, consistent with the in vivo results, while investigating the effect of GCN5 on cardiomyocyte apoptosis under hypertrophic stimulation, GCN5 overexpression further increased the expression of pro-apoptotic proteins, such as Bax and cleaved-caspase3, and decreased the level of anti-apoptotic protein Bcl2 (Fig. 3D). TdT-mediated dUTP nick-end labeling (TUNEL) staining was performed to further verify the pro-apoptotic effect of GCN5 on PE-treated cardiomyocytes (Fig. 3E).

### GCN5 deficiency alleviates PE-induced cardiomyocyte hypertrophy

**Fig. 4:**
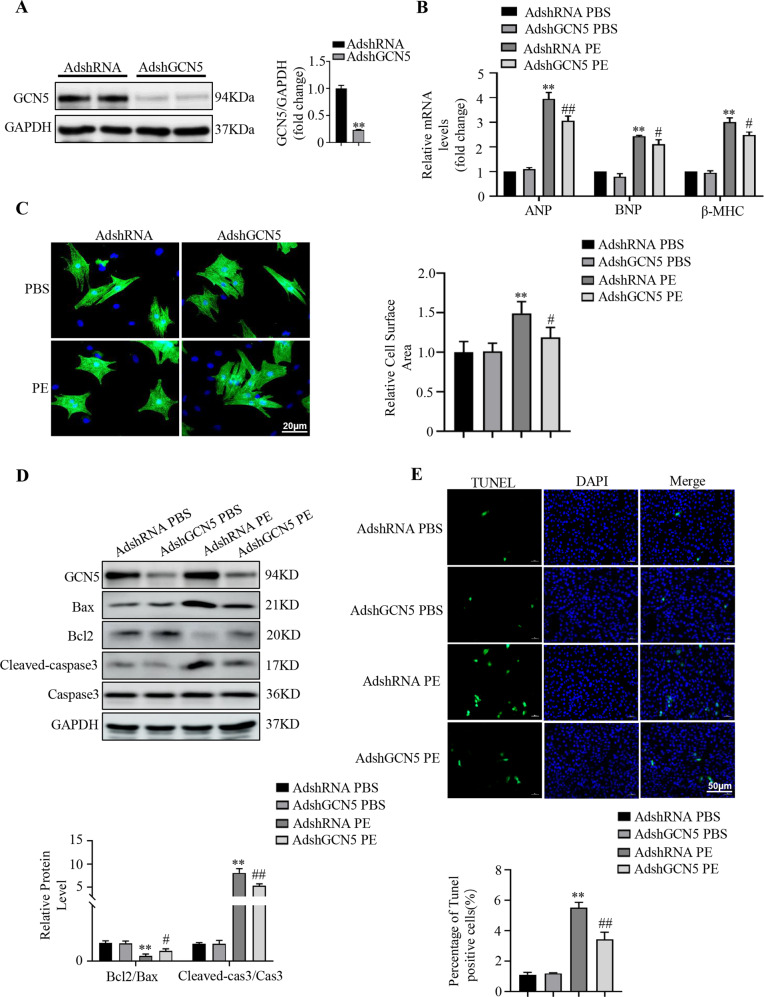
GCN5 deficiency alleviates PE-induced cardiomyocyte hypertrophy and apoptosis. **A** Immunoblotting of primary cardiomyocytes infected with AdshRNA or AdshGCN5 for 24 h to induce GCN5 expression. **B** Effects of GCN5 knockdown on the mRNA levels of hypertrophic marker genes. **C** Effects of GCN5 knockdown on cardiomyocyte surface areas. **D** Western blots showing that GCN5 knockdown alleviates PE-induced NRCM apoptosis. **E** Representative images of TUNEL staining, indicating apoptotic rates of NRCMs (^∗∗^*P* < 0.01 vs AdshRNA PBS; ^#^*P* < 0.05 vs AdshRNA PE, ^##^*P* < 0.01 vs AdshRNA PE). Data are shown as the mean ± SD from three independent experiments.

Considering the effects of GCN5 overexpression on PE-treated cardiomyocytes, we performed adenovirus-mediated GCN5 knockdown for further study (Fig. 4A). Consistent with previous results, under PE treatment, deficiency of GCN5 reduced cardiac hypertrophy marker expression at the mRNA and protein levels (Fig. 4B, Supplementary Fig. 2C).

Additionally, the cell surface areas of cardiomyocytes infected with adenoviruses containing GCN5-targeted small hairpin RNAs (AdshGCN5) were smaller than those infected with adenoviruses containing non-targeting small hairpin RNA (AdshRNA) (Fig. 4C). In addition, the expression of Bax and cleaved-caspase3 was inhibited by GCN5 knockdown (Fig. 4D). The ratio of PE-induced TUNEL-positive cardiomyocytes also decreased (Fig. 4E). These findings confirm the critical role of GCN5 in the regulation of PE-induced cardiomyocyte hypertrophy and apoptosis in vitro.

### GCN5 regulates TAK1-JNK/p38 signaling pathway in vivo and in vitro

**Fig. 5:**
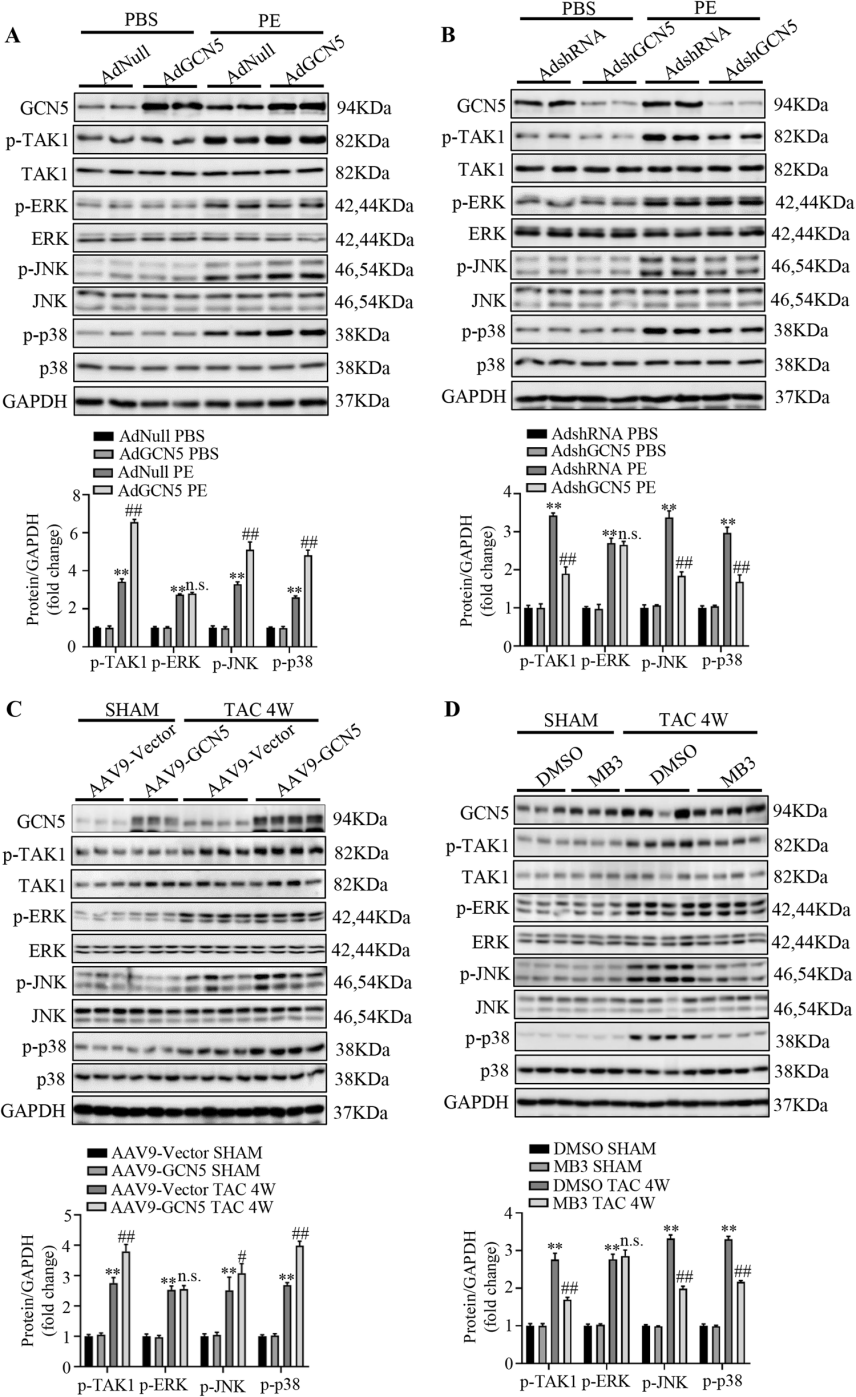
GCN5 regulates TAK1-JNK/p38 signaling pathway in hypertrophic hearts and cardiomyocytes. **A** Western blots showing the activity of TAK1-JNK/p38 signaling pathway components in NRCMs infected with AdNull or AdGCN5 for 24 h, followed by treatment with PBS or PE (^∗∗^*P* < 0.01 vs AdNull PBS; n.s., ^##^*P* < 0.01 vs AdNull PE). **B** Western blots showing the activity of TAK1-JNK/p38 signaling pathway components in NRCMs infected with AdshRNA or AdshGCN5 for 24 h, followed by treatment with PBS or PE (^∗∗^*P* < 0.01 vs AdshRNA PBS; n.s., ^##^*P* < 0.01 vs AdshRNA PE). **C** Western blot showing the activity of TAK1-JNK/p38 signaling pathway components in the hearts of GCN5-overexpressing mice or control mice subjected to sham or TAC surgery at 4 weeks (*n* = 7–9 mice/group, ^∗∗^*P* < 0.01 vs AAV9-Vector SHAM; n.s., ^#^*P* < 0.05, ^##^*P* < 0.01 vs AAV9-Vector TAC 4 W). **D** Western blot showing the activity of TAK1-JNK/p38 signaling pathway components in the hearts of GCN5-inhibited mice or control mice subjected to sham or TAC surgery at 4 weeks (*n* = 7-8 mice/group, ^∗∗^*P* < 0.01 vs DMSO SHAM; n.s., ^##^*P* < 0.01 vs DMSO TAC 4 W).

As the role of GCN5 in cardiac hypertrophy was confirmed, we explored the downstream targets regulated by GCN5 in this process. Substantial amount of research has shown that MAPK signaling members, including ERK1/2, p38, and JNK1/2, play an important role in cardiac remodeling; moreover, the MAPK signaling pathway generally mediates cardiac hypertrophy. Therefore, we evaluated the levels of TAK1, ERK, JNK, and p38 in vitro and in vivo. The total levels of MAPK pathway components were at the same baseline in all groups. However, compared with the PBS or sham group, PE stimulation and TAC significantly increased the phosphorylation of TAK1, JNK, and p38; moreover, GCN5 overexpression resulted in higher levels of these phosphorylated proteins (Fig. 5A, C). We also evaluated whether adenovirus-mediated GCN5 knockdown in NRCMs inhibited the phosphorylation of TAK1, JNK, and p38 (Fig. 5B).

Given that GCN5 is an acetyltransferase, many studies have used butyrolactone 3 (MB3) as a specific inhibitor of GCN5 [24–26]. Hence, MB3 was administered to mice via intraperitoneal injection under sham or TAC surgery for 4 weeks. The related data are presented in Supplementary Fig. 3. In agreement with the above results, inhibition of GCN5 may block the phosphorylation of TAK1, JNK, and p38 in mice, but not the levels of ERK (Fig. 5D). Collectively, these data indicate that GCN5 overexpression upregulates the TAK1-JNK/p38 signaling pathway, in cardiac hypertrophy, whereas inhibition of GCN5 downregulates it.

### Inhibition of TAK1 impairs the exaggerated hypertrophic response of GCN5 both in vivo and in vitro

**Fig. 6:**
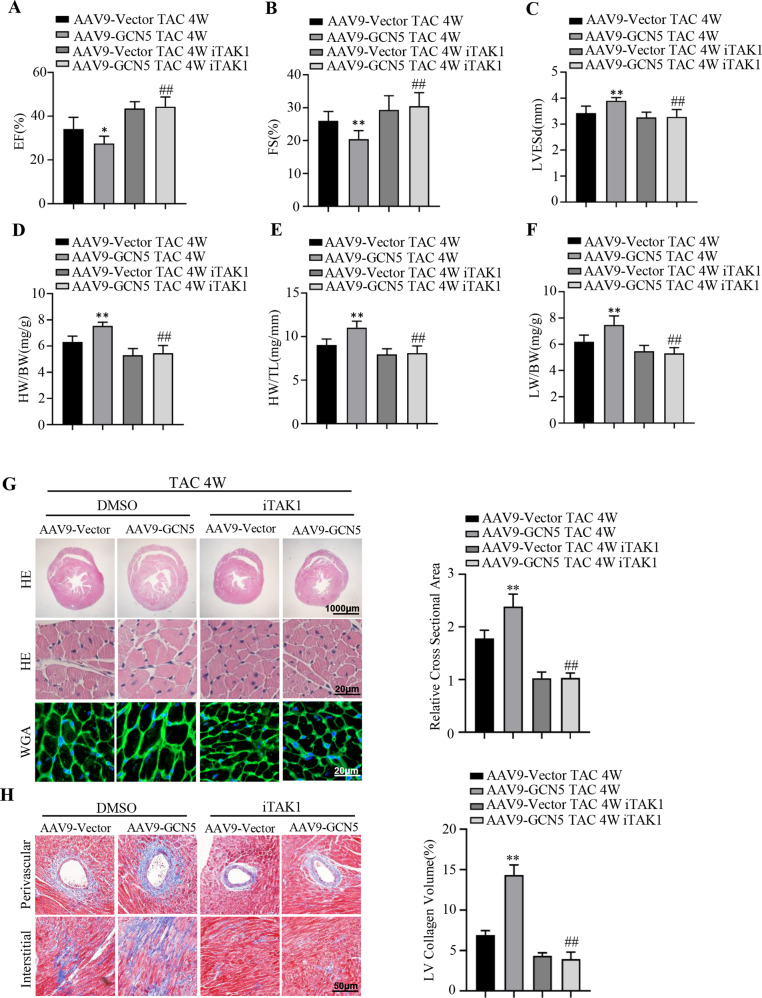
Inhibition of TAK1 attenuates the exaggerated hypertrophic effect of GCN5 overexpression. **A**–**C** Effects of iTAK1 on EF, FS, and LVESd values in GCN5-overexpressing mice and control mice after sham or TAC surgery (*n* = 6–7 mice/group). **D**–**F** Effects of iTAK1 on HW/BW, HW/TL, and LW/BW ratios in GCN5-overexpressing mice and control mice after sham or TAC surgery (*n* = 6–7 mice/group). **G** iTAK1-induced histological changes in heart sections stained with H&E and WGA to analyze heart and cardiomyocyte size, respectively. (*n* = 6-7 mice/group). **H** iTAK1-induced histological changes in heart sections stained with Masson’s trichrome stain to analyze perivascular and interstitial fibrotic area (*n* = 6–7 mice/group, ^∗^*P* < 0.05, ^∗∗^*P* < 0.01 vs AAV9-Vector TAC 4 W, ^##^*P* < 0.01 vs AAV9-GCN5 TAC 4 W).

**Fig. 7:**
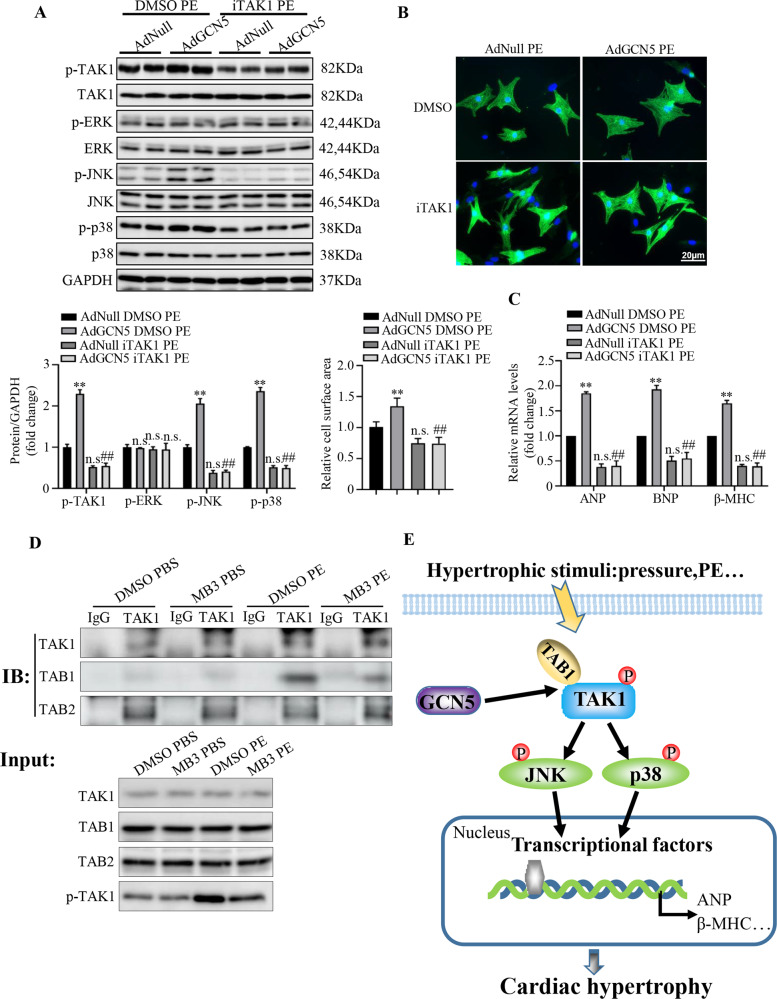
Inhibition GCN5 activity reduces binding of TAB1 and TAK1 and blocks TAK1 activation. **A** Western blots showing the activity of TAK1-JNK/p38 signaling pathway components in NRCMs infected with AdNull or AdGCN5 for 24 h, under treatment with PE and DMSO or iTAK1. **B** Effects of iTAK1 on cardiomyocyte surface areas in NRCMs infected with AdNull or AdGCN5 under PE treatment. **C** Effects of iTAK1 on hypertrophic marker genes in NRCMs infected with AdNull or AdGCN5 for 24 h under PE treatment (n.s., ^∗∗^*P* < 0.01 vs AdNull DMSO PE; n.s., ^##^*P* < 0.01 vs AdGCN5 DMSO PE; n.s. vs AdGCN5 iTAK1 PE). **D** Effects of GCN5 inhibitor MB3 on binding of TAK1 with TAB1 and TAB2 in NRCMs under PE treatment. **E** Schematic diagram showing how GCN5 regulates cardiac hypertrophy. Data are shown as the mean ± SD from three independent experiments.

To determine whether the excessive activation of TAK1-JNK/p38 is required for GCN5 overexpression-mediated cardiac hypertrophy, 5Z-7-oxozeaenol and NG25 were used as specific inhibitors of TAK1 in vivo and in vitro, respectively [27]. GCN5-overexpressing mice were intraperitoneally injected with 5Z-7-oxozeaenol (5 mg/kg) under TAC surgery for 4 weeks. After inhibition of TAK1, there was no difference in the TAC-induced heart functions (Fig. 6A–C), as well as HW/BW, HW/TL, and LW/BW (Fig. 6D–F), between GCN5-overexpressing and control mice; similar findings were noted following histological analyses (Fig. 6G, H). In vitro, NRCMs were stimulated with PE with or without NG25. Compared with the dimethyl sulfoxide (DMSO)- and PE-treated GCN5-overexpressing NRCMs, iTAK1 recovered the levels of phosphorylated TAK1, JNK, and p38 (Fig. 7A). In addition, following treatment with iTAK1, the cell surface (Fig. 7B) and mRNA levels of cardiac hypertrophy markers (Fig. 7C) were very similar, despite GCN5 overexpression and PE stimulation. These results indicate that TAK1 activation is vital for GCN5-mediated cardiac hypertrophy.

To further verify the role of TAK1 in GCN5-mediated cardiac hypertrophy with genetic strategy, TAK1 silencing model was established in vitro experiments using by si-TAK1. First, western blot was used to screen an effective TAK1 knockdown sequence. Results showed that si-TAK1-3 had the best knockdown efficiency (Supplementary Fig. 4A). During the subsequent experiments, si-TAK1-3 was used for silencing TAK1. According to the results, compared with AdGCN5+si-NC + PE group, the phosphorylation of TAK1, JNK, and p38 was obviously reduced in AdGCN5+si-TAK1 + PE group (Supplementary Fig. 4B). In addition, the cell surfaces in AdGCN5+si-TAK1 + PE group were smaller than AdGCN5+si-NC + PE group, mRNA levels of cardiac hypertrophy markers in AdGCN5+si-TAK1 + PE group were lower than AdGCN5+si-NC + PE group (Supplementary Fig. 4C-D). These results adequately demonstrated that knocking down TAK1 with genetic strategy and intervening TAK1 with specific inhibitors had the same effects on cardiac hypertrophy. TAK1 was a key point in GCN5-mediated cardiac hypertrophy.

### Inhibition of GCN5 activity decreases binding of TAB1 with TAK1 and blocks TAK1 activation

The results of GCN5-mediated regulation of cardiac hypertrophy via activated TAK1 prompted us to investigate why GCN5 may affect the phosphorylation of TAK1. MB3 had no effect on GCN5 expression levels in the heart tissues treated with TAC operation or primary NRCMs treated with PE stimulation (Supplementary Fig. 5A-B). Previous studies identified that GCN5 was a component of Ada two A containing (ATAC) complexes, and TAK1 was a cofactor of ATAC. Using co-immunoprecipitation (co-IP) assays, results revealed endogenous TAK1 was to be stably associated with native ATAC complex elements in HEK293 cells, including GCN5, ADA3, and STAF36 [28, 29]. To study the potential relationship between GCN5 and TAK1, co-IP assays were performed in our study, the results demonstrated protein-protein interaction between GCN5 and TAK1 under PE stimulation, while MB3 did not affect the interaction levels during disease conditions (Supplementary Fig. 5C-D). As a member of lysine acetyltransferases, the combination of GCN5 and TAK1 prompted us to detect whether GCN5 could affect the acetylation modification level of TAK1, the results showed that the level of acetylated TAK1 was not influenced by MB3 treatment (Supplementary Fig. 5E), indicating that GCN5 might affect activation of TAK1 through the other ways in pathological cardiac hypertrophy conditions. TAK1 is a regulator of the MAPK signaling pathway, which operates in response to signals from TGF-β and other cytokines, such as TNF-a and IL-1. TAK1 forms a heterotrimeric complex with TAK1 binding proteins (TAB1 and TAB2 or TAB3), which may bind to the N and C terminus of TAK1, respectively [30]. In vivo activation of TAK1 requires association with TAB1, which triggers phosphorylation of TAK1 [31], while during processes such as inflammation, TAB2 or TAB3 seem to be important for binding with TAK1, after stimulation, to activate TAK1 [32]. We found that in NRCMs, inhibition of GCN5 expression by MB3 decreased binding of TAB1 with TAK1 (Fig. 7D). Therefore, we speculated that GCN5 may regulate TAK1 activation by affecting TAB1 and TAK1 binding in certain ways (Fig. 7E).

## Discussion

Heart failure is a growing public health concern in modern society. Cardiac hypertrophy is an important pre-pathological condition of heart failure. Pathological cardiac hypertrophy is also a common pathological process in the development of many cardiovascular diseases, such as ischemic heart disease and valvular heart disease. Persistent pathological hypertrophy eventually leads to cardiac dysfunction and increased morbidity due to heart failure [33, 34]. Therefore, it is necessary to identify targets for prevention and treatment of cardiac hypertrophy.

Many previous studies have revealed the relationship between GCN5 expression and cancers [35, 36]; however, the expression and function of GCN5 in cardiac hypertrophy remain unclear. In the current study, we found increased expression of GCN5 at the mRNA and protein levels, following hypertrophic stimulation. In vertebrates, there are two highly similar GCN5-like paralogs: GCN5 and p300/CBP-associated factor (PCAF). GCN5 and PCAF proteins are ubiquitously expressed in adult mouse tissues and exert various biological effects. In mouse embryos, GCN5 seems to be expressed more widely and at higher levels than PCAF [31]. GCN5, a member of the GCN5-related N-acetyltransferase family of KATs, participates in the post-translational modification of multiple proteins. In addition, to histone H3, there is a growing list of non-histone proteins are involved in multiple pathophysiological processes. For example, GCN5-mediated histone acetylation governs nucleosome dynamics in spermiogenesis [37], GCN5 acetylates TUBA/alpha-tubulin in vivo and regulates the migration of vascular smooth muscle cells [38], and GCN5 has the ability to regulate cellular energetic and metabolic processes [39]. In cardiovascular systems, GCN5 has been reported to be associated with cardiac mesoderm specification and cardiogenesis [40].

To verify the effect of GCN5 on pressure overload-induced cardiac hypertrophy, gain- and loss-of-function approaches were applied in vivo and in vitro. AAV-GCN5 was injected through the tail vein of mice to overexpress GCN5. After TAC surgery, it accelerated the development of cardiac hypertrophy, as evidenced by increased cardiomyocyte cross-sectional area, fibrosis/collagen volume, and cardiac hypertrophic marker expression. Moreover, inhibition of GCN5 led to the opposite phenotype in TAC model mice, which further confirmed this conclusion. GCN5 was overexpressed or knocked down in NRCMs in vitro, which revealed that GCN5 overexpression aggravated PE-induced cardiomyocyte hypertrophy. Based on the above results, GCN5 aggravated the severity of cardiac hypertrophy under the presence of TAC or PE treatment, while GCN5 overexpression alone was not sufficient to initiate or promote cardiac hypertrophy. It further enlightened us that the increase in expression of GCN5 was a response to cardiac stress.

The MAPK signaling pathway is involved in the development of pathological cardiac hypertrophy. Previous studies have shown that MAPK signaling consists of the MAP3K-MAP2K-MAPK cascade. TAK1, a member of the MAP3K family, was discovered a few decades ago, and significant progress has been made toward elucidating its role and activation mechanism. During innate immunity, TAK1 plays a pivotal role in activation of signaling, in response to different stimuli, including cytokines, TLRs, stress, or pathogen infection [10]. Moreover, TAK1 plays an important role in cardiovascular diseases, as evidenced by many previous studies. We found that GCN5 has the ability to induce excessive activation of TAK1 in NRCMs, under treatment with PE; an inhibitor of TAK1 was used to confirm this effect of GCN5 on TAK1. Ubiquitination and phosphorylation of TAK1 and its binding partners (TAB1, TAB2, and TAB3) are key factors that affect activation of downstream molecules, such as NF-kB, JNK, and p38 MAPKs. Using a GCN5-specific inhibitor, we found that the binding of TAK1 with TAB1 was reduced. As reported in a previous study, GCN5 and PCAF have been found in SPT3-TAF9-GCN5/PCAF acetylase (STAGA) complexes. In humans, the complexes contain different components, and after extensive purification and subsequent identification, TAK1 was found to be a member of these complexes [31]. These findings provide ideas for further research to explore the exact relationship between GCN5 and TAK1 in cardiac hypertrophy.

A limitation of the current study is that the mechanism by which GCN5 affects TAK1 activation is unclear. Based on previous studies, we speculate several possible mechanisms. First, GCN5 may affect the expression of ubiquitinated/deubiquitinated or phosphorylated/dephosphorylated enzymes, which are associated with the activation of TAK1, thereby affecting TAK1 activation. Second, it is possible that the effect of GCN5 on TAK1 may occur in the STAGA complexes, such as conformational changes in space and the transformation of the interactions between components. However, the interaction between GCN5 and TAK1 requires further exploration.

In summary, we demonstrated a novel effect of GCN5 on cardiac hypertrophy. GCN5 aggravates pressure overload-induced cardiac hypertrophy by activating the TAK1-JNK/p38 signaling pathway. These results indicate GCN5 to be a potential target for therapeutic interventions in pathological cardiac hypertrophy and heart failure.

## Materials and methods

### Animals

Male C57BL/6 mice (8-10-week-old; 25–27 g) were purchased from the Research Center of the Southern Model Organisms (Shanghai, China). All animals were housed in temperature-controlled cages with a 12-h light-dark cycle and provided free access to food and water. All animal study procedures were approved by the Institutional Animal Care and Use Committee of the Ethics Committee of Tongji Medical College, Huazhong University of Science and Technology and conformed to the National Institutes of Health (NIH) Guide for the Care and Use of Laboratory Animals.

### Reagents

Antibodies against the following proteins were purchased from Cell Signaling Technology (Danvers, MA, USA): GCN5 (3305, 1:1000 dilution), p-TAK1 (9339, 1:1000 dilution), TAK1 (5206, 1:1000 dilution), p-ERK1/2 (4370, 1:1000 dilution), ERK1/2 (4695, 1:1000 dilution), p-JNK (4668, 1:1000 dilution), JNK (9252, 1:1000 dilution), p-p38 (4511, 1:1000 dilution), p38 (9212, 1:1000 dilution), TAB1 (3226, 1:1000 dilution), TAB2 (3,745, 1:1000 dilution), Acetylated-Lysine Antibody (9441 S, 1:1000 dilution) and GAPDH (5174, 1:1000 dilution). Antibodies against ANP (sc20158, 1:200 dilution) and β-MHC (sc53090, 1:200 dilution) were obtained from Santa Cruz Biotechnology (Dallas, TX, USA). Phenylephrine (P6126) and antibody against α-actinin (A7811, 1:100 dilution) were obtained from Sigma-Aldrich (St. Louis, MO, USA). A BCA protein assay kit, obtained from Thermo Fisher (Waltham, MA, USA), was used to determine the protein concentrations. MB3 (HY-129039), NG25 (HY-15434), and 5Z-7-oxozeaenol (HY-12686) were purchased from MCE (Shanghai, China).

### TAC surgery and inhibitor treatment

Cardiac hypertrophy was induced in mice through TAC surgery with partial aortic arch construction, as previously described [27]. Briefly, male mice were anesthetized with pentobarbital sodium (50 mg/kg) via intraperitoneal injection. After the toe pinch reflex disappeared, median thoracotomy was performed at the second intercostal space to expose the transverse aorta, which was tied against a 27-gauge needle; the transverse aorta was constricted using a 7-0 silk suture ligature tied firmly. After ligation, the needle was removed gently. Sham subjects underwent the same operation, except for ligation. The mice were observed until recovery on a heated pad set at 37 °C, and all mice were allocated randomly to the TAC or sham group. Mice were intraperitoneally injected with the GCN5-specific inhibitor MB3 at a concentration of 5 mg/kg once every 2 days; mice subjected to TAC were intraperitoneally injected with the TAK1 inhibitor 5Z-7-oxozeaenol at a concentration of 5 mg/kg for 4 weeks, *n* = 7–8 mice/group [34].

### Echocardiography

Cardiac function was evaluated with transthoracic echocardiography using small animal ultrasound machine (Vevo 2100, Canada). After inhalation anesthesia of isoflurance, cardiac dimensions were measured with M-mode echocardiography. The EF and FS values were calculated using computer algorithms. LVEDd and LVESd were measured by a trained observer blinded to the experimental groups.

### Histological analyses

The hearts were excised at 4 weeks after TAC, and the BW, HW, LW, and TL were recorded for further analysis. The hearts were fixed in 10% formalin and embedded in paraffin following standard histological procedures. Serial 5-μm-thick sections were obtained. Sections were stained with hematoxylin and eosin, wheat germ agglutinin, and Masson’s trichrome stain. For immunohistochemistry, the heart sections were stained using primary antibodies using an immunohistochemistry kit. Histological changes were observed and photographed using a microscope (Zeiss, Germany), and the cross-sectional area of the cardiomyocytes and degree of fibrosis were estimated using Image-Pro Plus v6.0 software.

### NRCM culture and treatment

Primary NRCMs were isolated from 1-2-day-old Sprague-Dawley rats using Hank’s balanced salt solution containing 0.03% trypsin and 0.04% collagenase type II. Fibroblasts were removed using a differential attachment technique [41]. Purified NRCMs were plated at a density of 1 × 10^6^ cells/well in gelatin-coated six-well culture plates and cultured in Dulbecco’s modified Eagle’s medium (DMEM) or F12 (C11330, Gibco) medium containing 10% fetal bovine serum, penicillin (100 U/ml), streptomycin (100 U/ml), and 5-bromodeoxyuridine (0.1 mM, inhibitor of fibroblast proliferation) at 37 °C for 24 h with 5% CO_2_. The NRCMs were starved and incubated with PE (50 μM) for 24 h to induce cardiomyocyte hypertrophy.

### Recombinant adenovirus and adeno-associated virus production

To overexpress or knockdown GCN5 in vitro, NRCMs were infected with AdGCN5 or AdshGCN5. AdNull was used as control for AdGCN5, it was packaged with an empty vector, the vector of AdNull and AdGCN5 was pAdTrack-CMV-3 × FLAG, GCN5 cDNA sequence used for constructing AdGCN5 was from Rat. AdshRNA was used as control for AdshGCN5, and AdshRNA was packaged with a meaningless sequence (TTCTCCGAACGTGTCACGT), AdshGCN5 was packaged with an effective silencing sequence (GCAGGGTGTTCTGAACTTT). Silencing sequences were designed for the Rat species, the vector of AdshRNA and AdshGCN5 was pADV-U6-shRNA-CMV-MCS.

In vivo, AAV9-GCN5 was applied to transduce cardiomyocytes overexpressing GCN5 via tail vein injection with 2 × 10^11^ viral particles, *n* = 10–12 mice/group. GCN5 was stably overexpressed after injecting three weeks. AAV9-Vector was used as control, it was packaged with an empty vector, GCN5 cDNA sequence used for constructing AAV9-GCN5 was from mouse. The specific information of AAV9-Vector and AAV9-GCN5 was listed in Supplementary Fig. 6.

### Quantitative real-time polymerase chain reaction (PCR)

Total RNA from primary cells and heart tissues was extracted using TRIzol reagent (Thermo Fisher Scientific). Total RNA was converted into cDNA using a reverse transcription kit (Takara, Japan). Quantitative real-time PCR amplification was performed using SYBR Green (Takara), and GAPDH gene expression was used for normalization. The primer sequences used are listed in Supplementary Table 1.

### Western blotting

Tissues and primary cells were lysed using RIPA buffer supplemented with protease inhibitor cocktail (Roche, Switzerland). Protein concentrations were quantified using a BCA Protein Assay Kit (Thermo Fisher Scientific). Extracted proteins were separated via SDS-PAGE, and the proteins were transferred to polyvinylidene difluoride membranes (Millipore, Bedford, MA, U.S.A.). The membranes were blocked in 5% skim milk and incubated with the respective primary antibody overnight at 4 °C. After incubation with peroxidase-conjugated secondary antibodies (Jackson ImmunoResearch Laboratories, 1:10 000 dilution). The signal was detected and quantified using Amersham Imager 680 (GE, USA). Three independent experiments were performed.

### Immunofluorescence analysis

To measure the cell surface area of cardiomyocytes, immunofluorescence staining was performed as described previously [42]. Cardiomyocytes were treated with PE (50 μM) or PBS after infection with the corresponding adenovirus for 24 h at 37 °C, under 5% CO_2_, following which they were fixed with 4% paraformaldehyde, permeabilized with 0.2% Triton X-100 in PBS, and stained with an α-actinin antibody. The cells were observed and imaged using a fluorescence microscope (Zeiss, Germany). Image J software (NIH) was used to measure the cell size.

### TUNEL assay

A TUNEL assay kit (Roche) was used to evaluate the apoptosis index of primary cardiomyocytes, in which GCN5 was overexpressed or silenced, under PE stimulation according to the manufacturer instructions.

### Immunoprecipitation (IP)

The protein of primary neonatal rat cardiomyocytes (1000 µg) was incubated with TAK1 or IgG antibody (Abcam) and protein A/G beads (Thermo Fisher Scientific) overnight at 4 °C. The beads were washed three times and resuspended in 4× loading buffer. The expression of TAK1 and TAB1 were examined using western blotting. Experiments were performed in triplicate.

### Statistical analysis

All statistical analyses were performed with SPSS v22.0 (IBM Inc., Armonk, NY, USA). In all bar graphs, data were presented as the mean ± standard deviation from at least three independent experiments. Differences between the two groups were analyzed by Student’s two-taied *t* test. Differences between multiple groups were analyzed by one-way analysis of variance (ANOVA). *P* < 0.05 was considered statistically significant.

## Supplementary information

Responses for change confirming

Supplementary Table 1

Supplementary material

Original Data File

Author Contribution Statement

Reproducibility checklist

## Data Availability

The data and material that support the findings of this study are available from the corresponding author upon reasonable request.
