# Supplementary material for: GCN5-mediated regulation of pathological cardiac hypertrophy via activation of the TAK1-JNK/p38 signaling pathway
Source: Cell Death Dis. 2022 Apr 30;13(4):421. doi: 10.1038/s41419-022-04881-y (PMC9056507; doi:10.1038/s41419-022-04881-y)
Supplement: Supplementary file 1 — Responses for change confirming [file 41419_2022_4881_MOESM1_ESM.docx]

**Dear editors:**

On behalf of my co-authors, we would like to express our deeply appreciation to you for your kind consideration and efficient work on our manuscript. According to your opinion, for the first question, we supplemented the date availability statement in the manuscript *(Page 20, line 419-421)*. And for the second question, I emailed all co-authors with the change, and asked them to reply to my email confirming that they agree to the change. I saved complete screenshots of the specific contents of the emails and collected them here.

The email I sent to my co-authors was as follows:


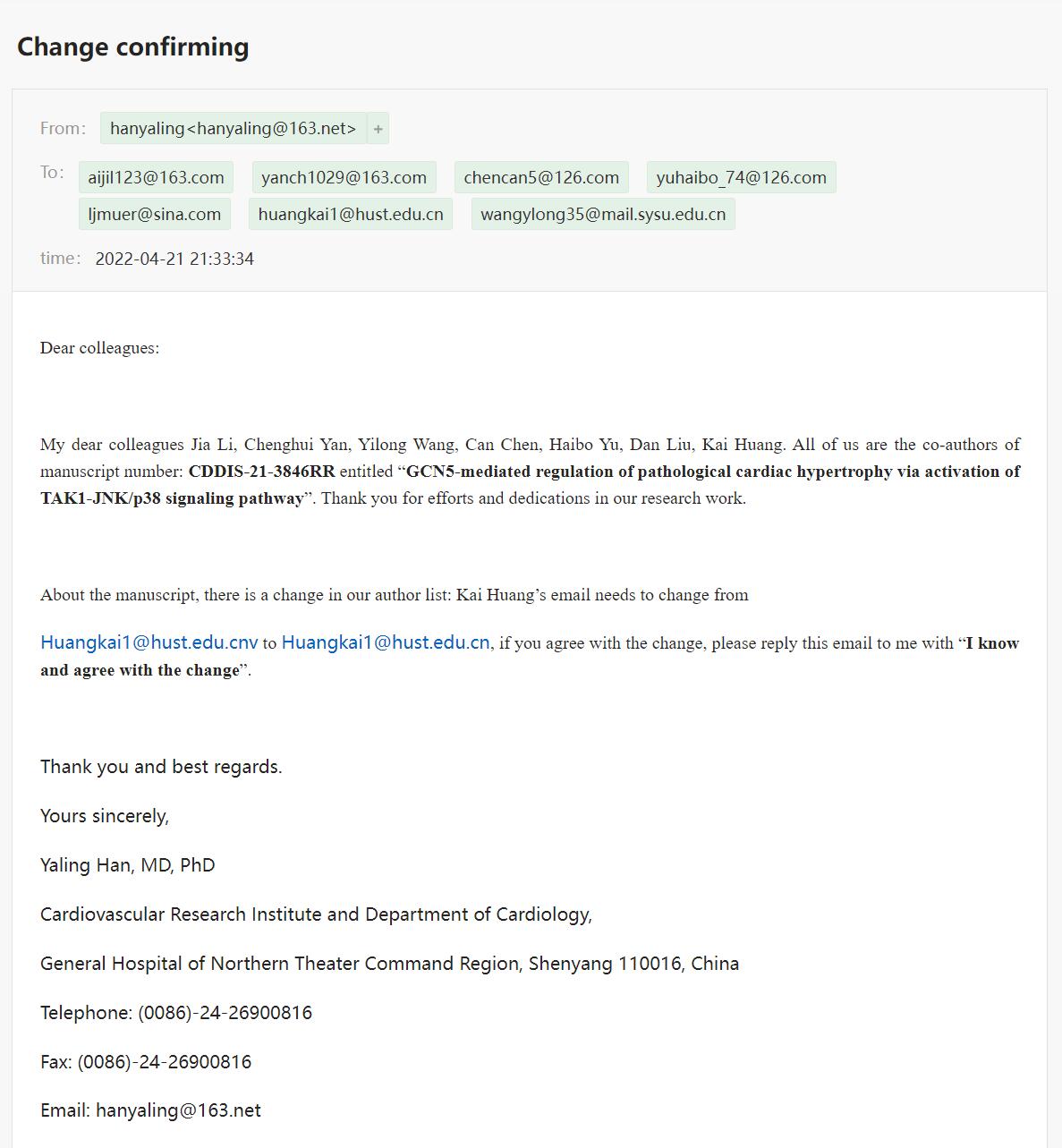


The responses from all co-authors were collected as follows:


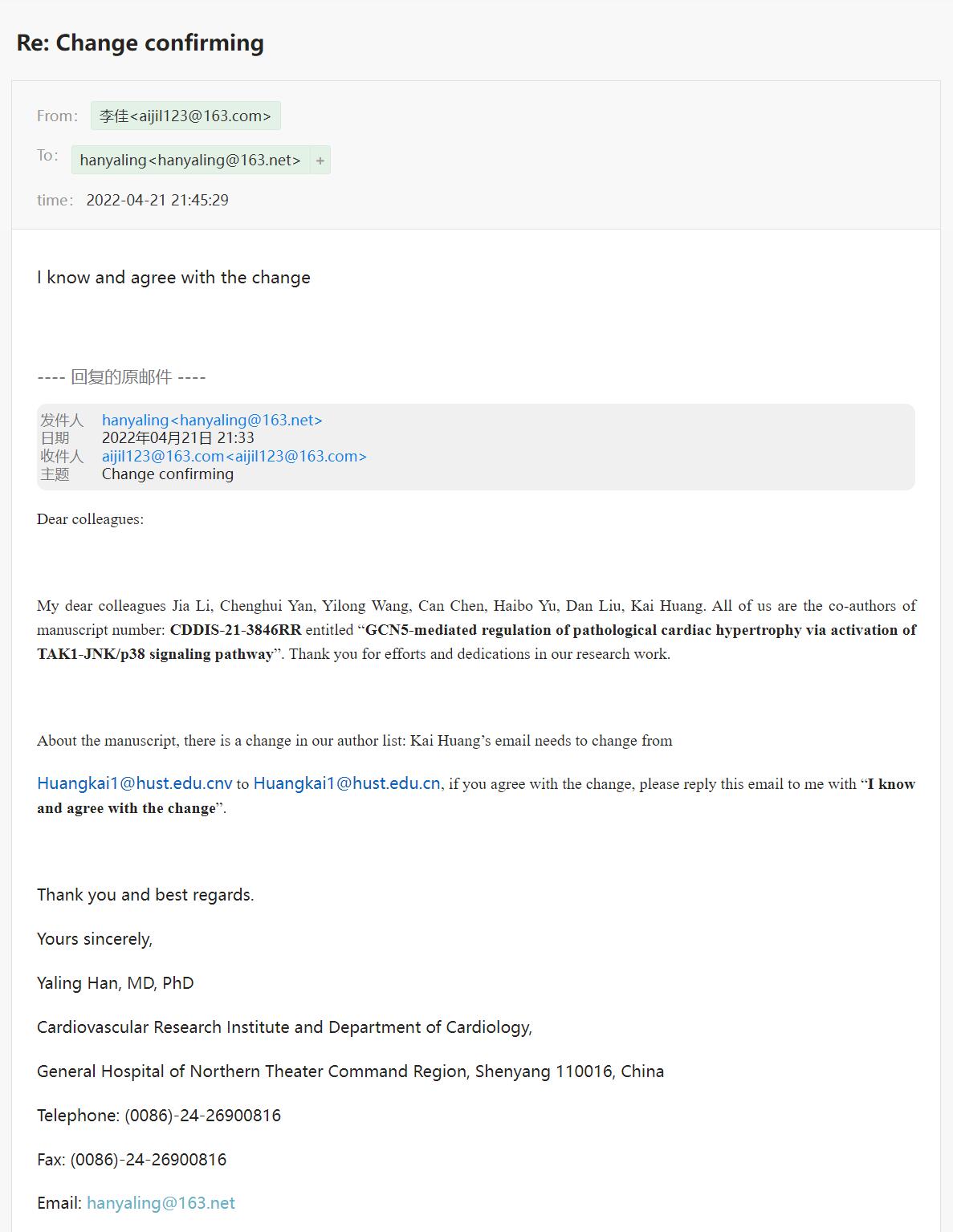


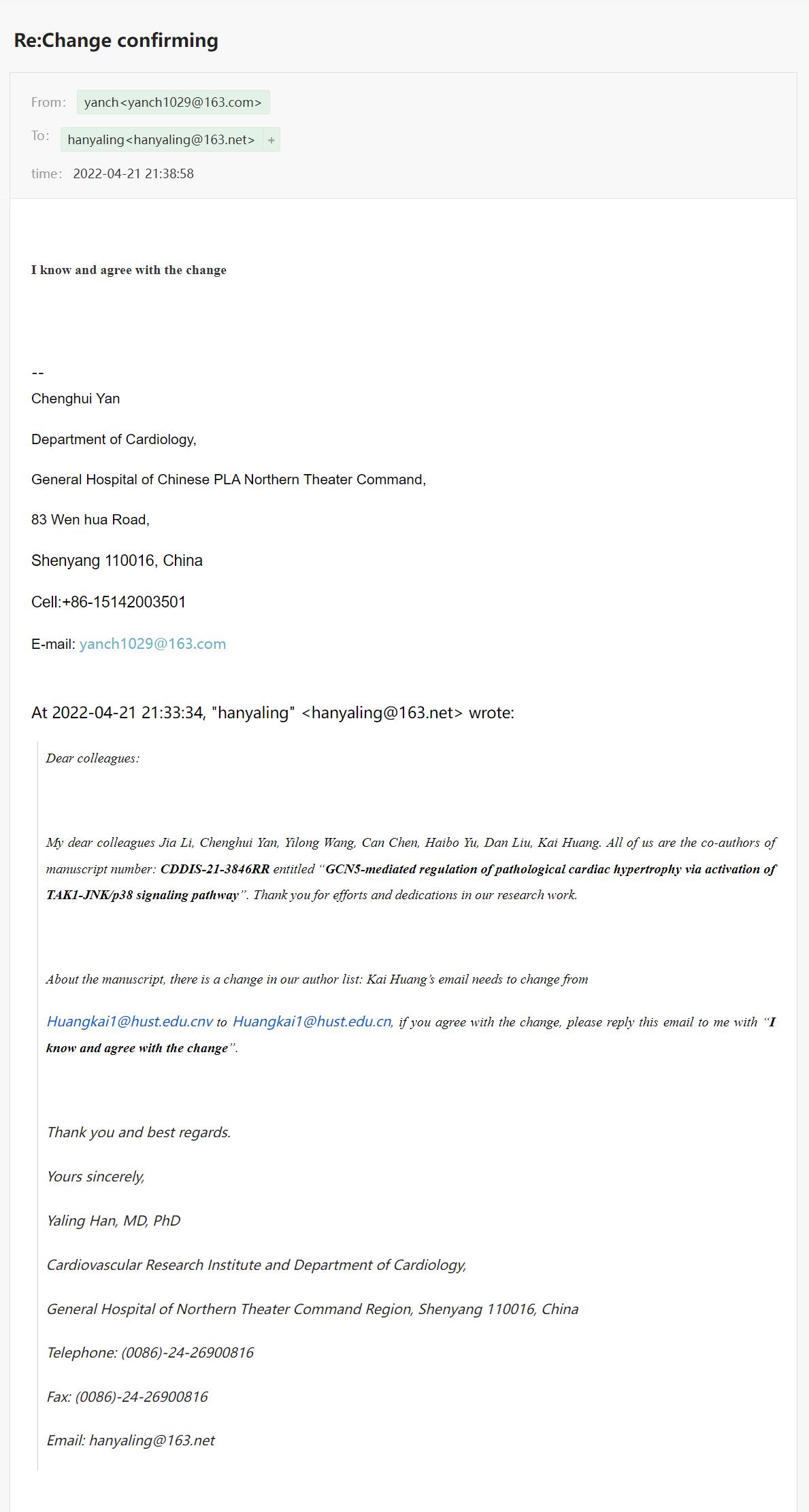


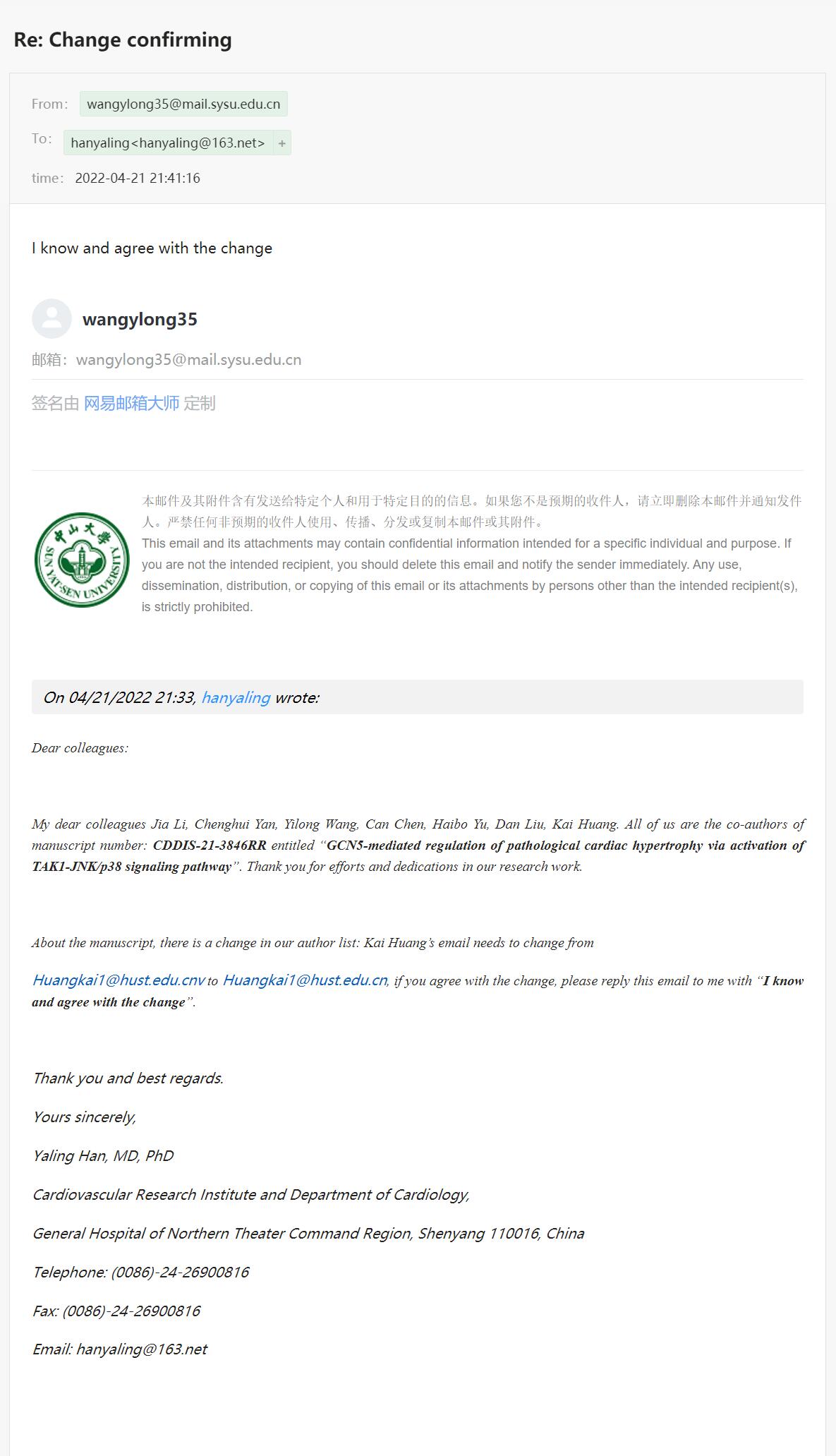


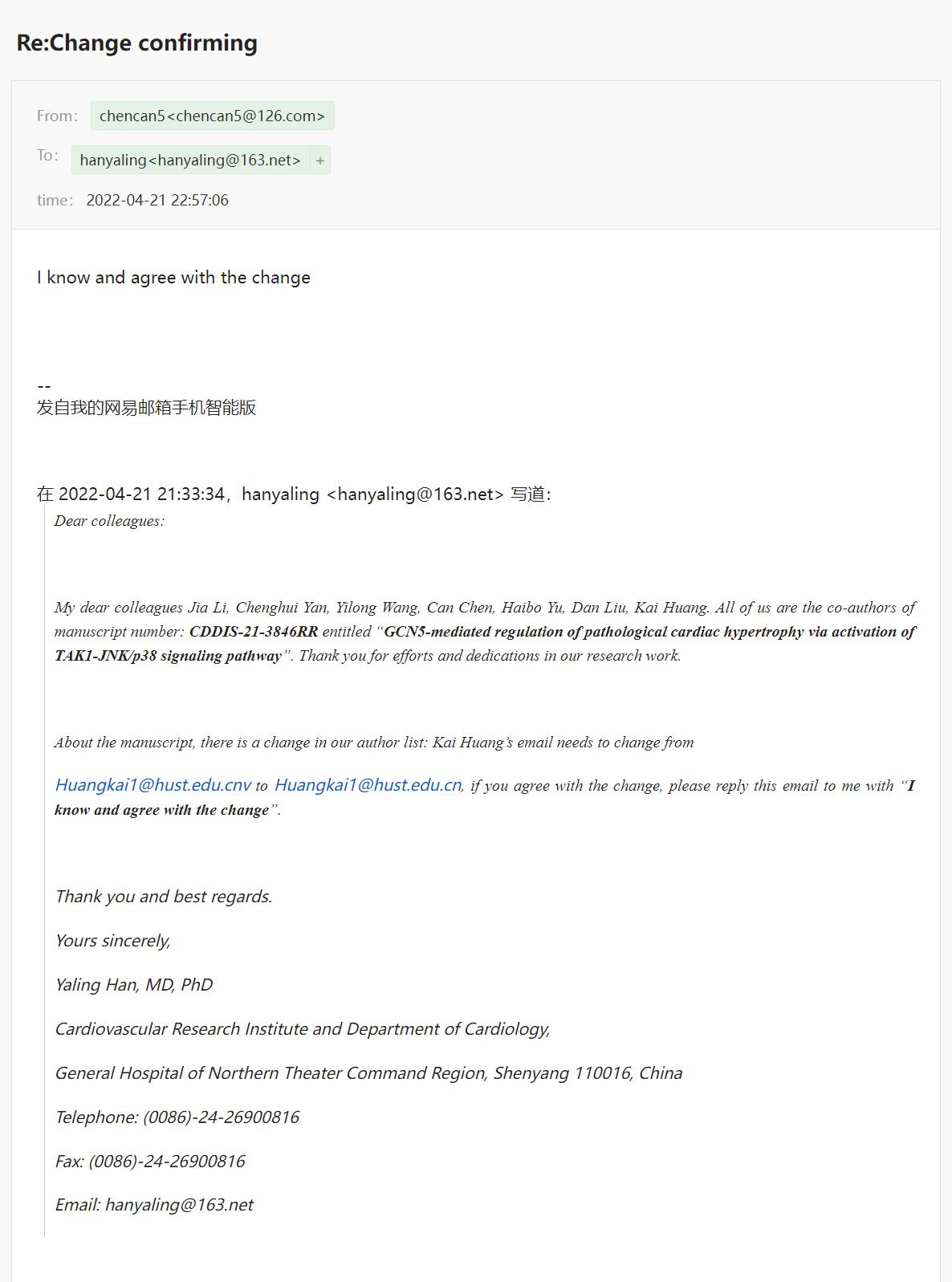


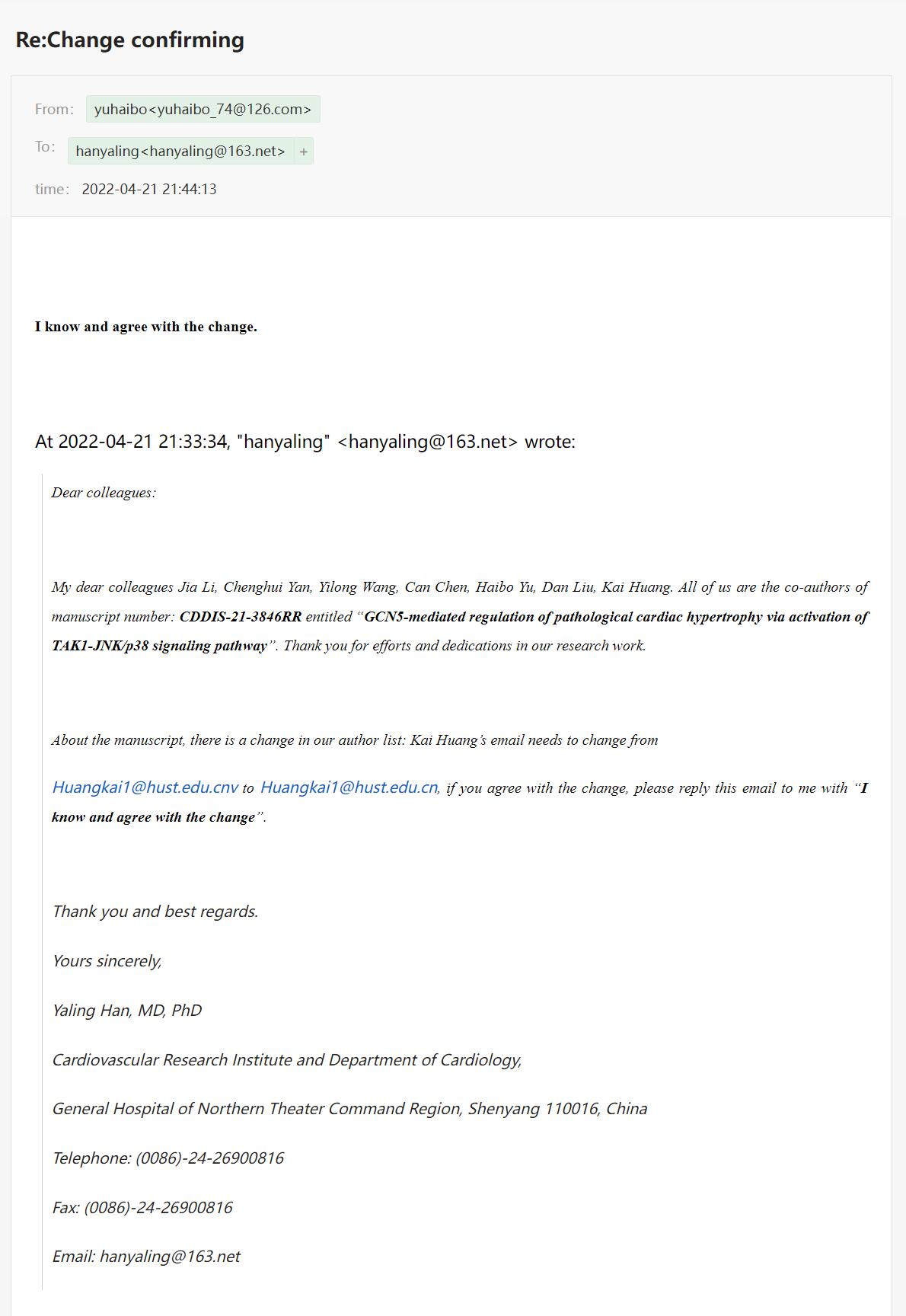


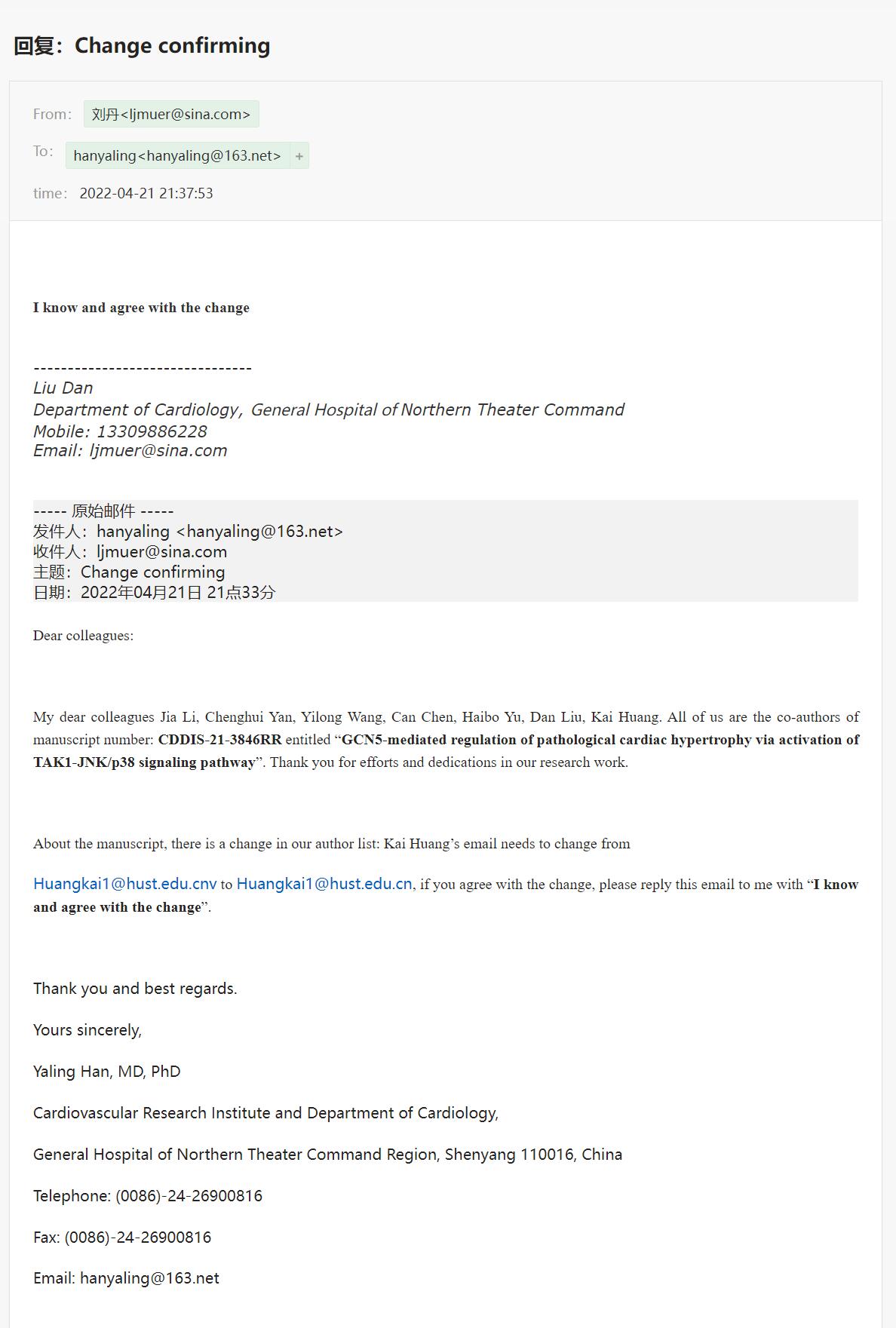


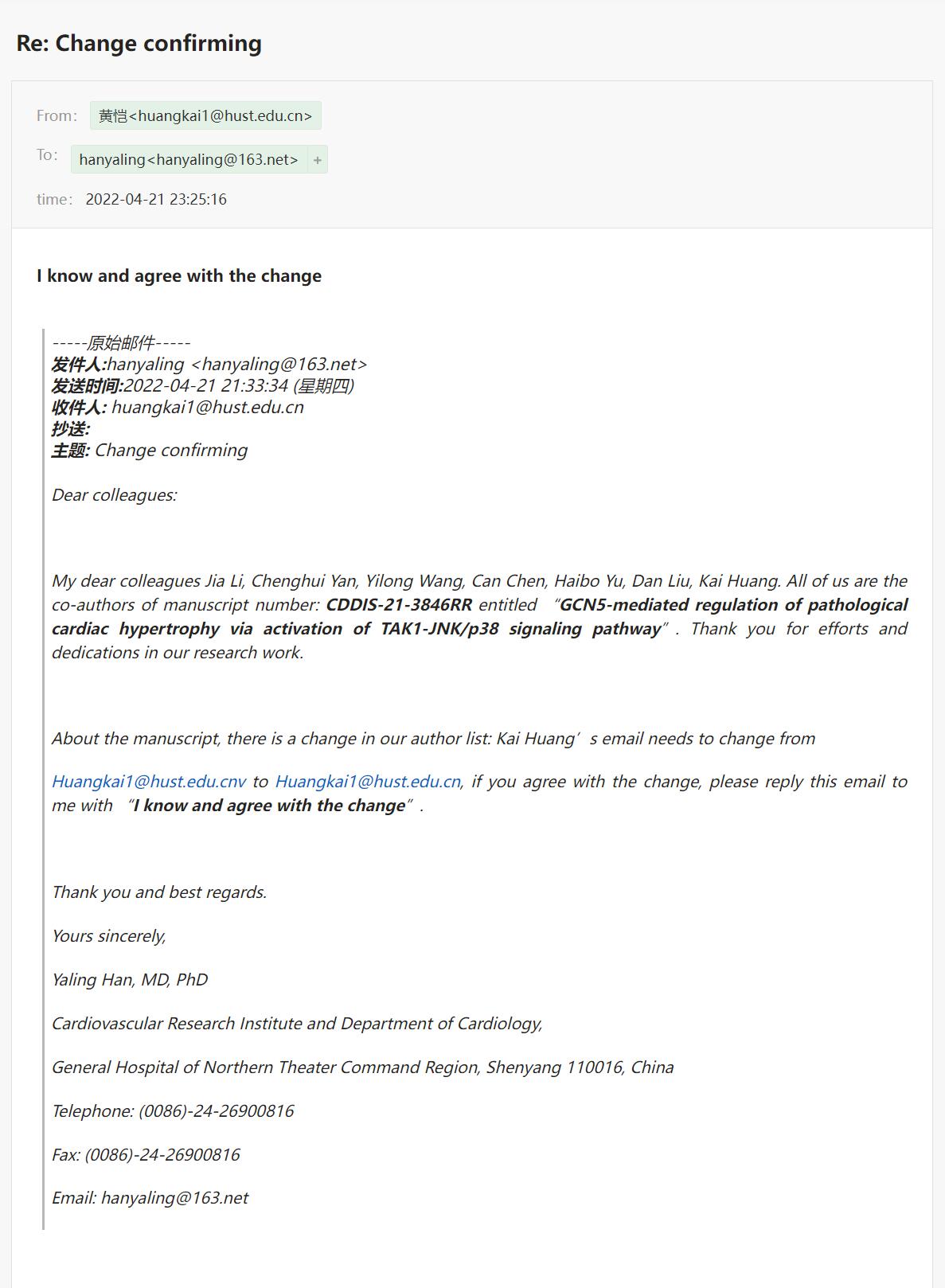


Thank you and best regards.

Yours sincerely,

Yaling Han, MD, PhD

Cardiovascular Research Institute and Department of Cardiology,

General Hospital of Northern Theater Command Region, Shenyang 110016, China

Telephone: (0086)-24-26900816

Fax: (0086)-24-26900816

Email: hanyaling@163.net
