# Supplementary material for: GCN5-mediated regulation of pathological cardiac hypertrophy via activation of the TAK1-JNK/p38 signaling pathway
Source: Cell Death Dis. 2022 Apr 30;13(4):421. doi: 10.1038/s41419-022-04881-y (PMC9056507; doi:10.1038/s41419-022-04881-y)
Supplement: Supplementary file 2 — Supplementary Table 1 [file 41419_2022_4881_MOESM2_ESM.docx]

**Supplementary Table 1. A list of the primers for Real-Time PCR**

| **Primer name** | **Forward Primer** | **Reverse Primer** |
| --- | --- | --- |
| GAPDH-Mouse | AGAACATCATCCCTGCATCC | AGTTGCTGTTGAAGTCGC |
| ANP-Mouse | ACCTGCTAGACCACCTGGAG | CCTTGGCTGTTATCTTCGGTACCGG |
| BNP-Mouse | GAAGGACCAAGGCCTCACAA | TTCAGTGCGTTACAGCCCAA |
| α-MHC-Mouse | TGCACTACGGAAACATGAAGTT | CGATGGAATAGTACACTTGCTGT |
| β-MHC-Mouse | CCGAGTCCCAGGTCAACAA | CTTCACGGGCACCCTTGGA |
| Collagen-1-Mouse | TGCTAACGTGGTTCGTGACCGT | ACATCTTGAGGTCGCGGCATGT |
| Collagen-3-Mouse | CCCAACCCAGAGATCCCATT | GAAGCACAGGAGCAGGTGTAGA |
| TGF-β-Mouse | GAGCCCGAAGCGGACTACTA | TGGTTTTCTCATAGATGGCGTTG |
| GCN5-Mouse | CAGATCCGCAAGGTCTACCC | TCCAGCCTGTCTCTCGAATG |
| GAPDH-Rat | TGTGAACGGATTTGGCCCTA | GATGGTGATGGGTTTCCCGT |
| ANP-Rat | CTGGGACCCCTCCGATAGAT | TTCGGTACCGGAAGCTGTTG |
| BNP-Rat | CAATCCACGATGCAGAAGCTG | GGCGCTGTCTTGAGACCTAA |
| β-MHC-Rat | CAGGCCAACACCAACCTGTC | TCTACTCTTCATTCAGGCCCTTGG |
| GCN5-Rat | ATTCCCCCATCTGGGAGTCA | CTCTTCTCTCCTGCTGGCAT |
