# Supplementary material for: GCN5-mediated regulation of pathological cardiac hypertrophy via activation of the TAK1-JNK/p38 signaling pathway
Source: Cell Death Dis. 2022 Apr 30;13(4):421. doi: 10.1038/s41419-022-04881-y (PMC9056507; doi:10.1038/s41419-022-04881-y)
Supplement: Supplementary file 3 — Supplementary material [file 41419_2022_4881_MOESM3_ESM.pdf]

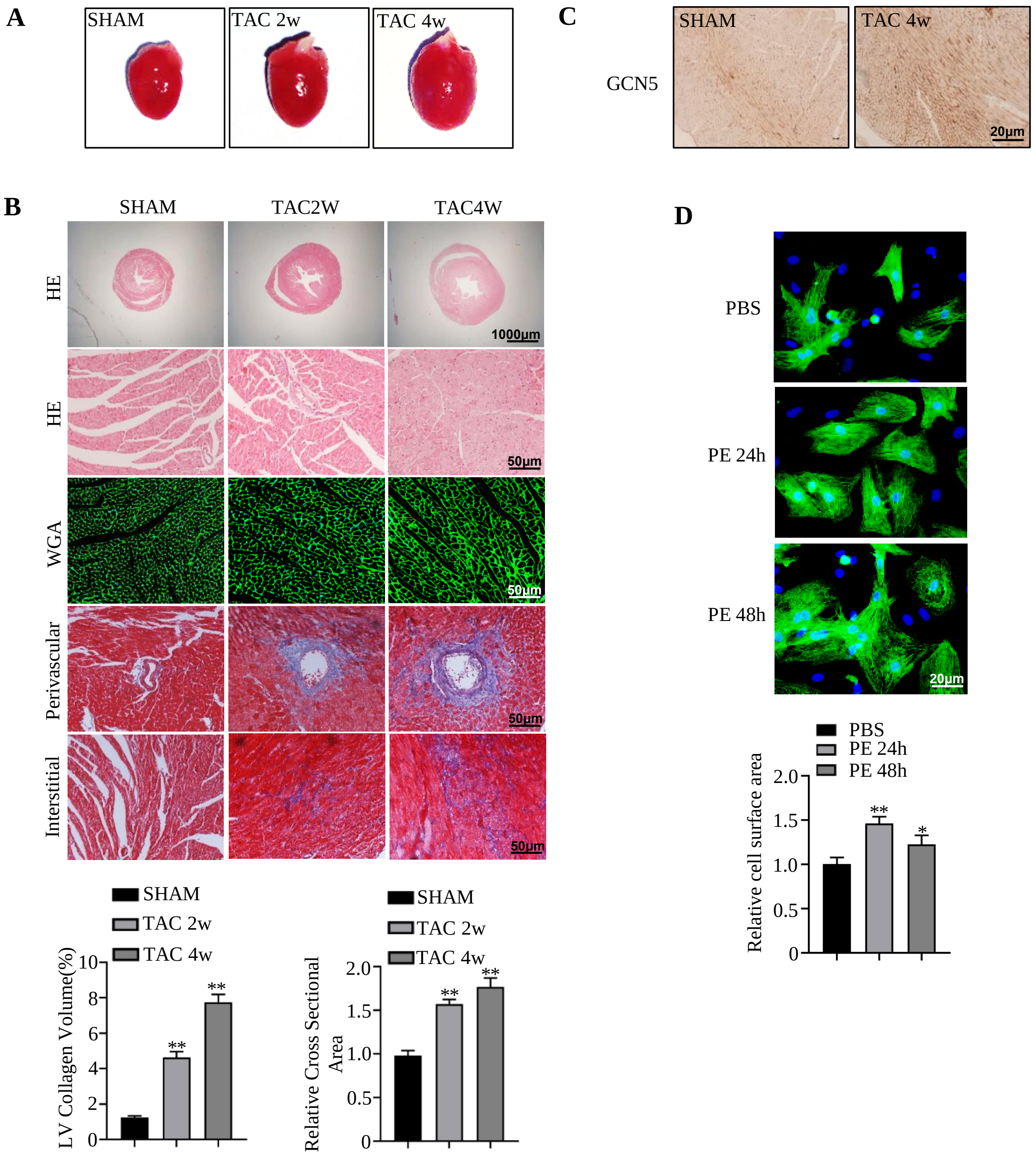

Fig. S1. Effects of TAC surgery and PE stimulation on cardiac remodeling. (A) Representative pictures showed the overall shape of heart at different time point after TAC surgery. (B) Images of heart sections stained with H&E, WGA, Masson to analyse cardiomyocyte size or perivascular and interstitial fibrotic area at different time point after TAC surgery. (n = 4 mice/time point, ** $P < 0.01$ vs SHAM). (C) Representative images of immunohischemistry with an anti-GCN5 antibody in slices from the hypertrophic mouse heart. (D) Representative images demonstrated the cell area of NRCMs stimulated with PE at different time point. (* $P < 0.05$ vs PBS, ** $P < 0.01$ vs PBS).

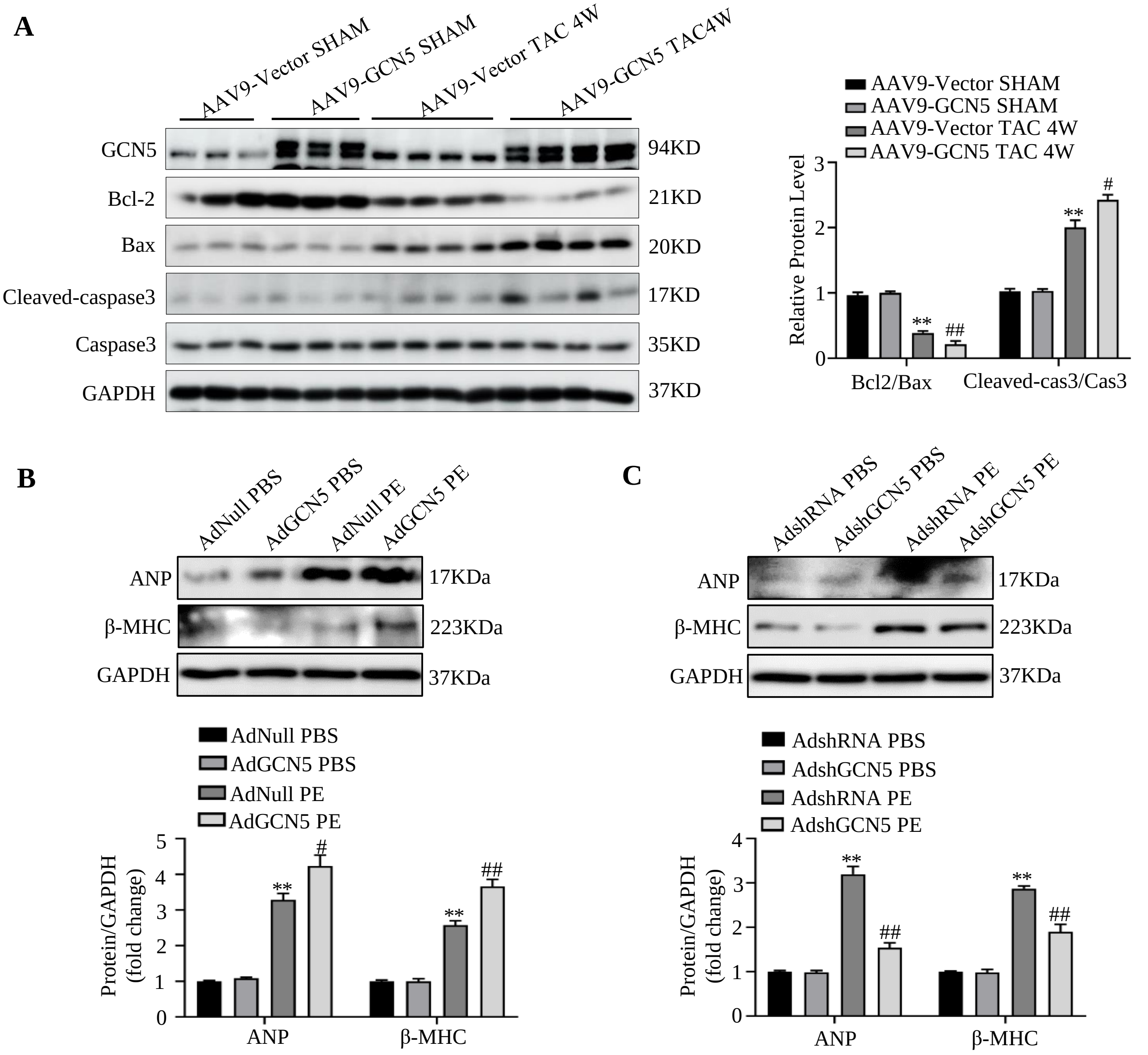

Fig. S2. GCN5 overexpression promotes cardiac hypertrophy and apoptosis after hypertrophic stimulations.

(A) Western blots showing overexpressed GCN5 exacerbates TAC-induced apoptosis. (n = 10-12 mice/group, ** $P < 0.01$ vs AAV-Vector SHAM; # $P < 0.05$, ## $P < 0.01$ vs AAV9-Vector TAC 4W). (B) Effects of GCN5 overexpression on the protein levels of hypertrophic marker genes. (** $P < 0.01$ vs AdNull PBS, # $P < 0.05$, ## $P < 0.01$ vs AdNull PE). (C) Effects of GCN5 knockdown on the protein levels of hypertrophic marker genes. (** $P < 0.01$ vs AdshRNA PBS; ## $P < 0.01$ vs AdshRNA PE). Data are shown as the means ± SDs. from three independent experiments.

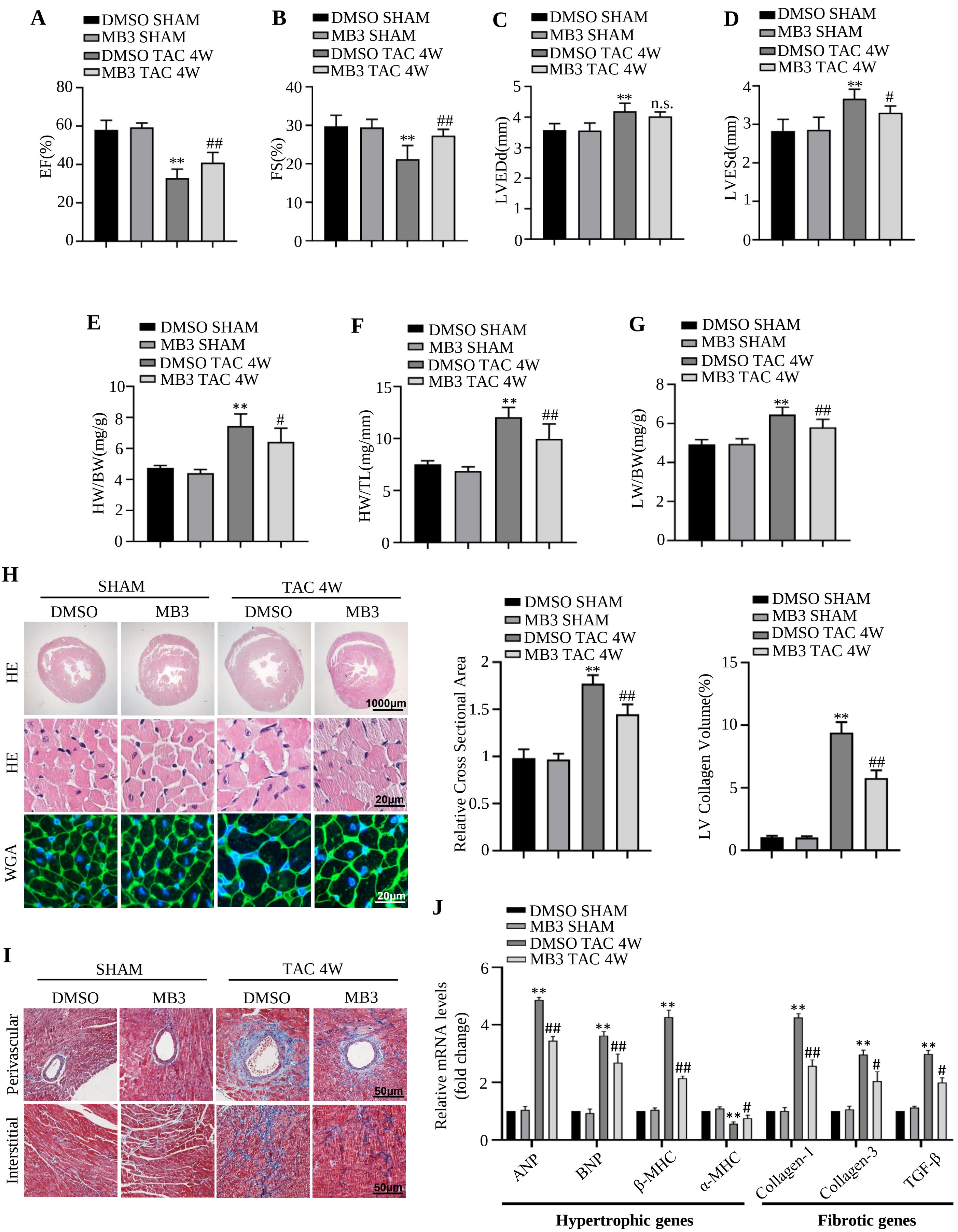

A
B
C
D
E
F
G
H
I
J
DMSO SHAM
MB3 SHAM
DMSO TAC 4W
MB3 TAC 4W
EF(%)
FS(%)
LVEDd(mm)
LVESd(mm)
n.s.
HW/BW(mg/g)
HW/TL(mg/mm)
LW/BW(mg/g)
SHAM
TAC 4W
DMSO
MB3
HE
WGA
1000μm
20μm
Relative Cross Sectional Area
LV Collagen Volume(%)
Perivascular
Interstitial
50μm
Relative mRNA levels (fold change)
ANP
BNP
β-MHC
α-MHC
Collagen-1
Collagen-3
TGF-β
Hypertrophic genes
Fibrotic genes

Fig. S3. Inhibition of GCN5 alleviates pressure overload-induced cardiac hypertrophy and dysfunction.

(A-D) Echocardiographic assessment of EF, FS, LVEDd, LVESd values in GCN5 inhibitor treated mice and control mice subjected to sham or TAC surgery at 4 weeks (n = 7-8 mice/group). (E-G) HW/BW ratios, HW/TL ratios, LW/BW ratios in GCN5 inhibitor treated mice and control mice subjected to sham or TAC surgery at 4 weeks (n = 7-8 mice/group). (H) Representative images of heart sections stained with H&E, WGA to analyse heart and cardiomyocyte size (n = 4-5 mice/group). (I) Representative images of heart sections stained with Masson to analyse perivascular and interstitial fibrotic area (n = 4-5 mice/group). (J) Effects of GCN5 inhibiting on the mRNA levels of the hypertrophic marker genes and fibrotic marker genes in the heart of mice subjected to sham or TAC surgery at 4 weeks (n= 5-6 mice/group, ∗∗ $P < 0.01$ vs DMSO SHAM; n.s., # $P < 0.05$, ## $P < 0.01$ vs DMSO TAC 4W). Data are shown as the means ± SDs. from three independent experiments.

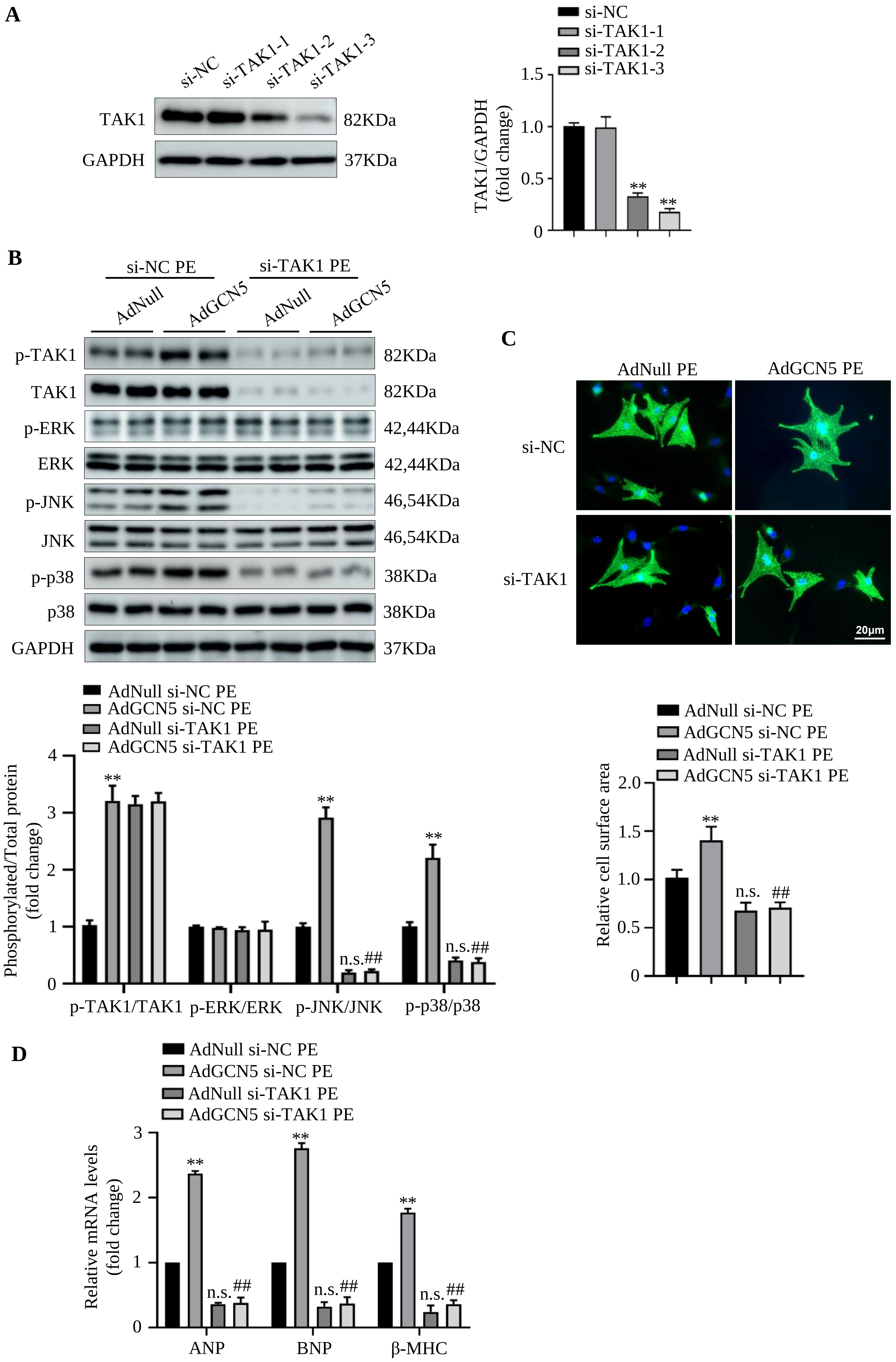
A
si-NC
si-TAK1-1
si-TAK1-2
si-TAK1-3
TAK1
82KDa
GAPDH
37KDa
TAK1/GAPDH (fold change)
1.5
1.0
0.5
0
**
**
B
si-NC PE
si-TAK1 PE
AdNull
AdGCN5
AdNull
AdGCN5
p-TAK1
82KDa
TAK1
82KDa
p-ERK
42,44KDa
ERK
42,44KDa
p-JNK
46,54KDa
JNK
46,54KDa
p-p38
38KDa
p38
38KDa
GAPDH
37KDa
AdNull si-NC PE
AdGCN5 si-NC PE
AdNull si-TAK1 PE
AdGCN5 si-TAK1 PE
Phosphorylated/Total protein (fold change)
4
3
2
1
0
**
**
n.s.##
**
n.s.##
p-TAK1/TAK1
p-ERK/ERK
p-JNK/JNK
p-p38/p38
C
AdNull PE
AdGCN5 PE
si-NC
si-TAK1
20μm
AdNull si-NC PE
AdGCN5 si-NC PE
AdNull si-TAK1 PE
AdGCN5 si-TAK1 PE
Relative cell surface area
2.0
1.5
1.0
0.5
0
**
n.s.
##
D
AdNull si-NC PE
AdGCN5 si-NC PE
AdNull si-TAK1 PE
AdGCN5 si-TAK1 PE
Relative mRNA levels (fold change)
3
2
1
0
**
**
**
n.s.
##
n.s.
##
n.s.
##
ANP
BNP
β-MHC

Fig. S4. Silencing TAK1 attenuates the exaggerated hypertrophic effect of GCN5 overexpression. (A) Knockdown efficiency of different silencing sequences (**P < 0.01 vs si-NC). (B) Effects of si-TAK1 on the activity of TAK1-JNK/p38 signaling pathway components in NRCMs infected with AdNull or AdGCN5 for 24 h, under treatment with PE. (C) Effects of si-TAK1 on cardiomyocyte surface areas in NRCMs infected with AdNull or AdGCN5 under PE treatment. (D) Effects of si-TAK1 on hypertrophic marker genes in NRCMs infected with AdNull or AdGCN5 for 24 h under PE treatment (**P < 0.01 vs AdNull si-NC PE; ##P < 0.01 vs AdGCN5 si-NC PE; n.s. vs AdGCN5 si-TAK1 PE). Data are shown as the means ± SDs. from three independent experiments.

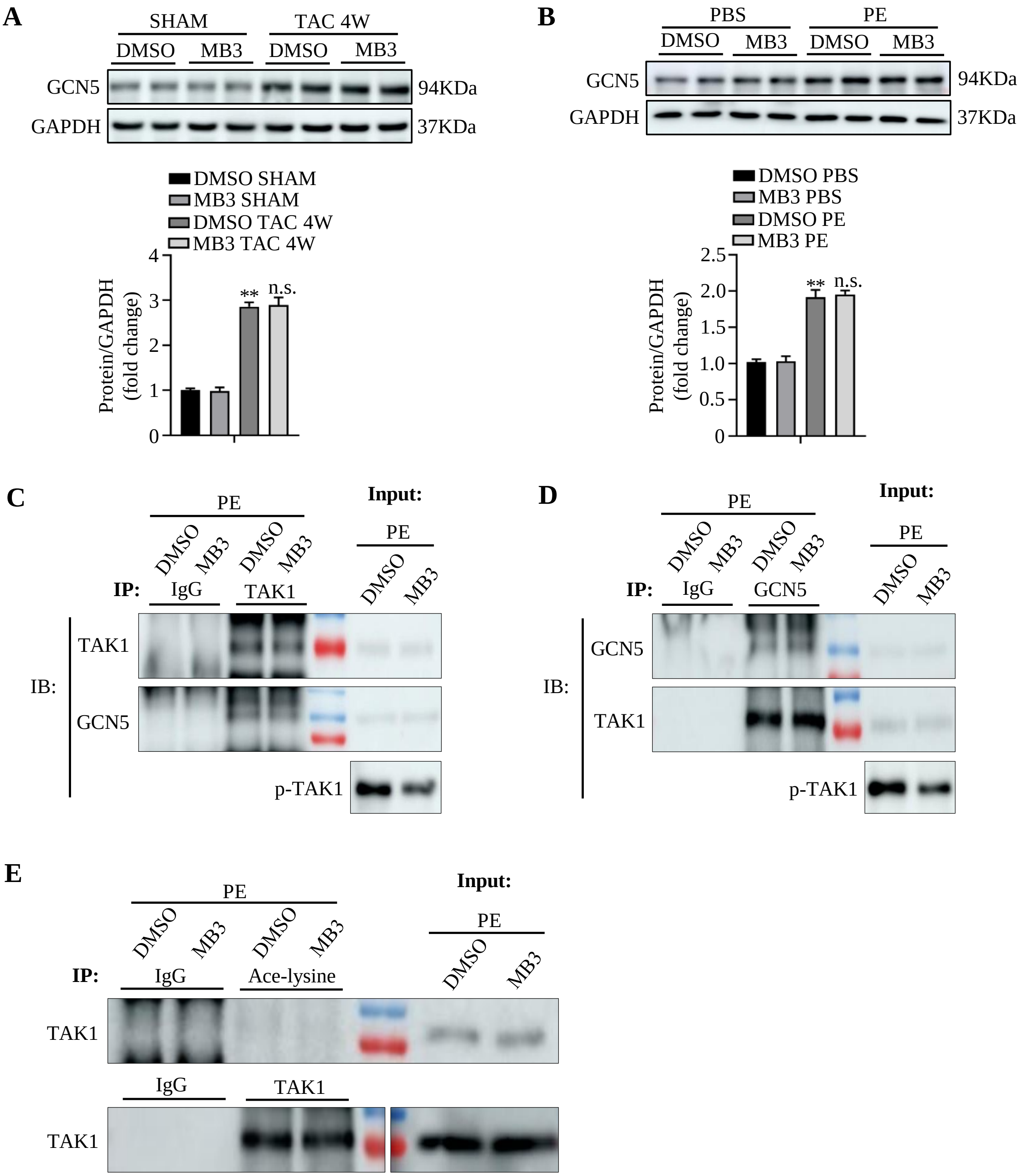

Fig. S5. Effects of MB3 on GCN5 expression and interaction between TAK1 and GCN5 in disease models.

(A) Western blots showing MB3 treatment has no effect on GCN5 expression in TAC models (∗∗ $P < 0.01$ vs DMSO SHAM, n.s. vs DMSO TAC4W). (B) Western blots showing MB3 treatment has no effect on GCN5 expression under PE stimulations (∗∗ $P < 0.01$ vs DMSO PBS, n.s. vs DMSO PE). (C-D) Effects of GCN5 inhibitor MB3 on binding of TAK1 and GCN5 (C: pull down with anti-TAK1 antibody, D: pull down with anti-GCN5 antibody). (E) Effects of GCN5 inhibitor MB3 on TAK1 acetylation in PE conditions. (∗∗ $P < 0.01$ vs DMSO SHAM, n.s. vs DMSO TAC 4W). Data are shown as the means ± SDs. from three independent experiments.

A

**Gene Name:**	Kat2a	
**GenBank ID:**	NM_020004.5	
**Gene size:**	2493	
**Species:**	Mouse	
**Upstream and downstream restriction enzyme cutting site:**	NheI	BamHI
**Resistance in prokaryotes:**	Amp	
**Name of Vector:**	H13559 pAAV-CMV-3×FLAG-P2A-mNeonGreen-tWPA	
**Name of Target:**	pAAV-CMV-Kat2a-3×FLAG-P2A-mNeonGreen-tWPA	
**Forward sequencing primer:**	CMV-F CGCAAATGGGCGGTAGGCGTG	

B

Plasmid map of empty vector (Before inserting the target gene)

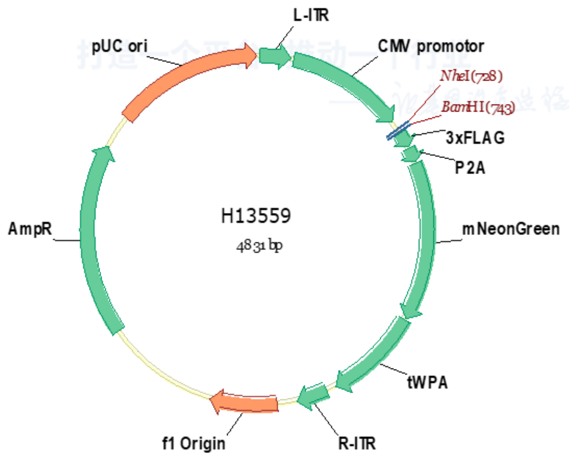

C

Plasmid map after inserting target gene

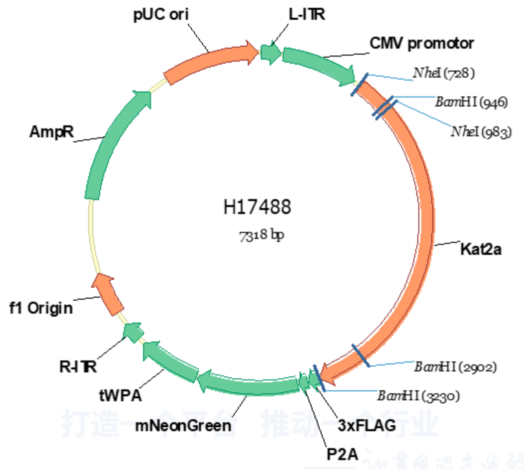

Fig. S6. Information for AAV9-Vector and AAV9-GCN5.
(A) Details for AAV-Vector and AAV-GCN5 (GCN5 is also named as Kat2a). (B) Plasmid map of empty vector. (C) Plasmid map of inserting GCN5 vector.
