# Supplementary material for: GCN5-mediated regulation of pathological cardiac hypertrophy via activation of the TAK1-JNK/p38 signaling pathway
Source: Cell Death Dis. 2022 Apr 30;13(4):421. doi: 10.1038/s41419-022-04881-y (PMC9056507; doi:10.1038/s41419-022-04881-y)
Supplement: Supplementary file 4 — Original Data File [file 41419_2022_4881_MOESM4_ESM.pptx]

## Slide 1
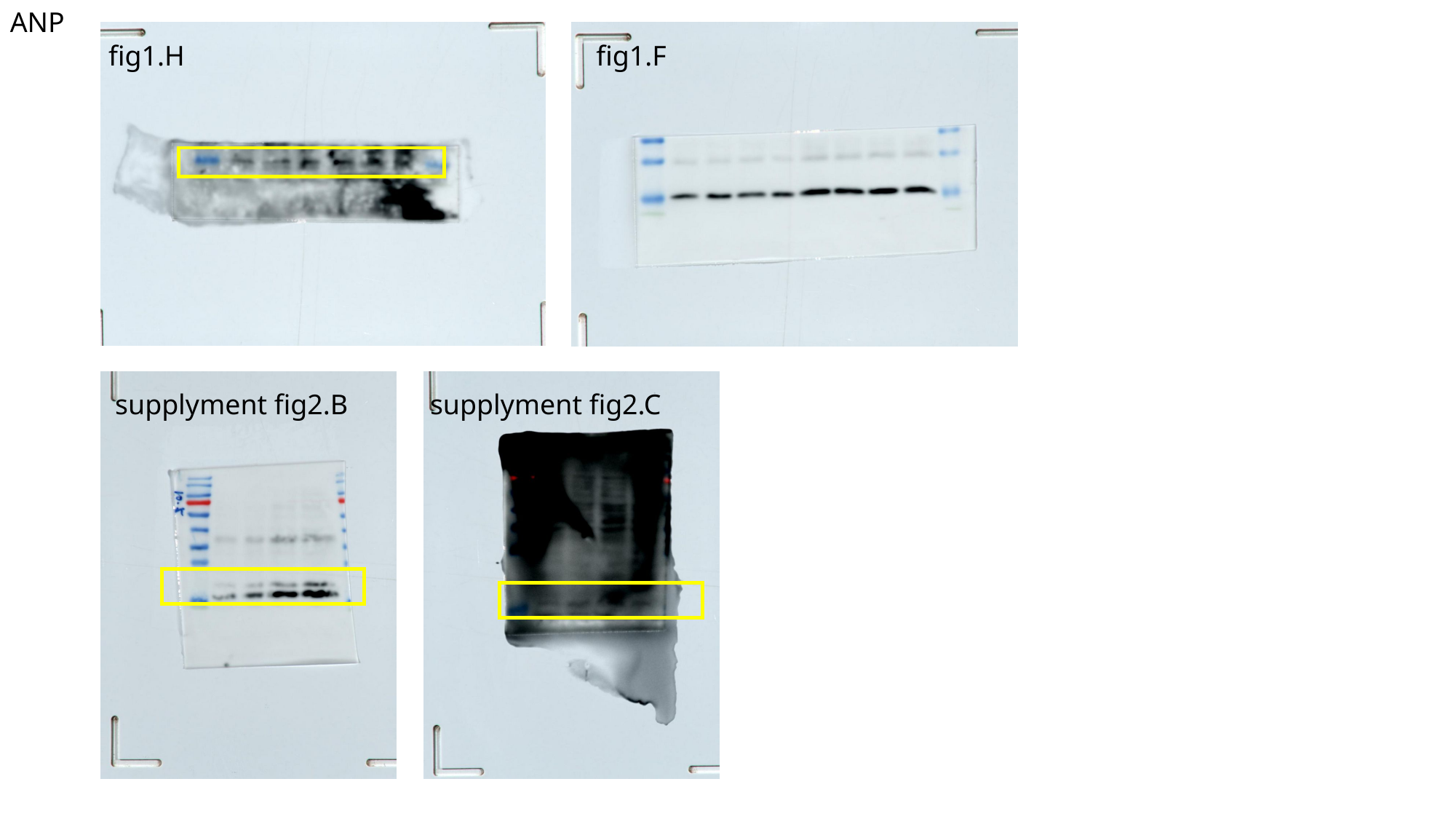

ANP
fig1.H
fig1.F
supplyment fig2.B
supplyment fig2.C

## Slide 2
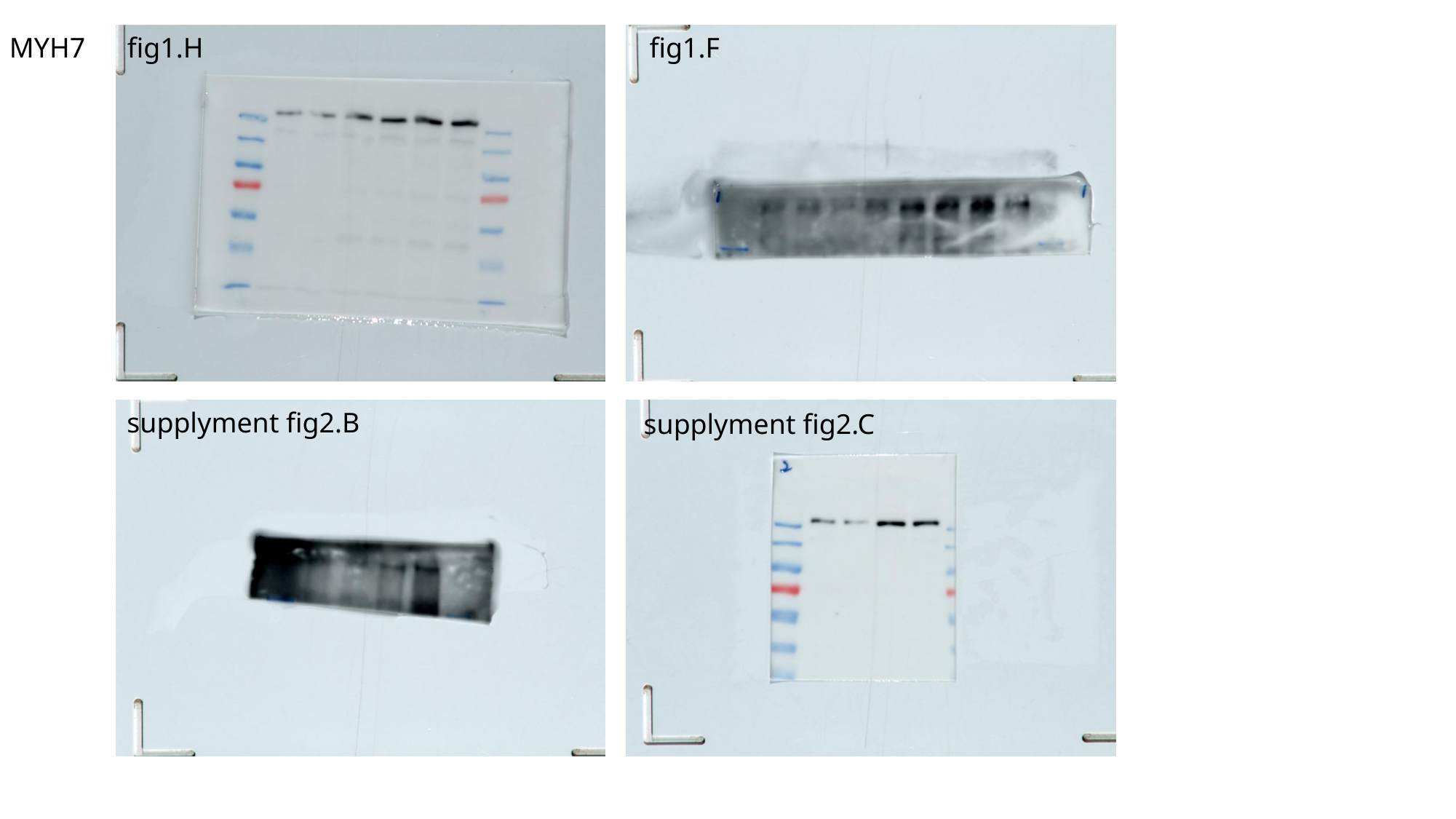

MYH7
fig1.H
fig1.F
supplyment fig2.B
supplyment fig2.C

## Slide 3
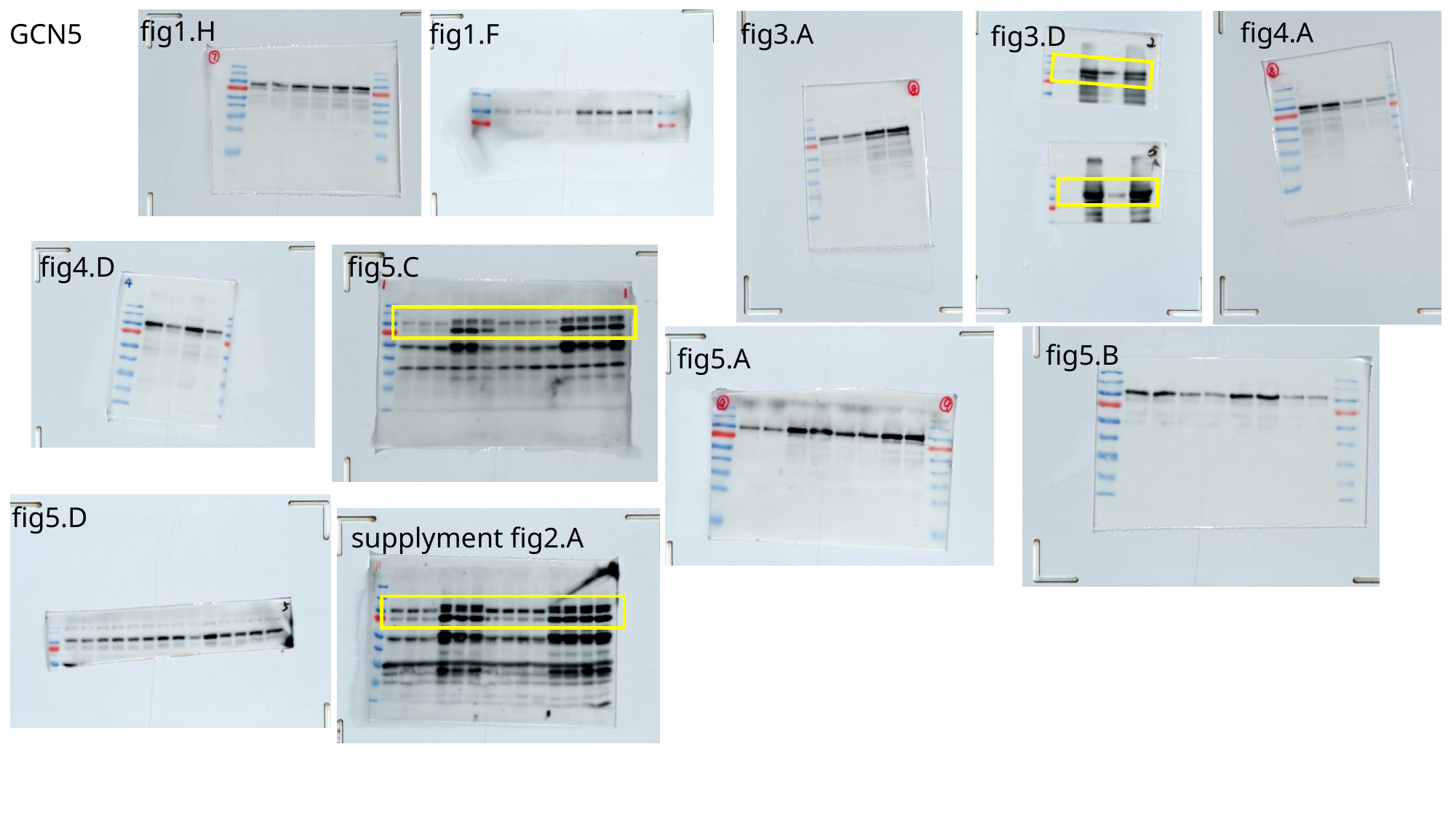

fig1.H
fig4.A
GCN5
fig1.F
fig3.A
fig3.D
fig4.D
fig5.C
fig5.B
fig5.A
fig5.D
supplyment fig2.A

## Slide 4
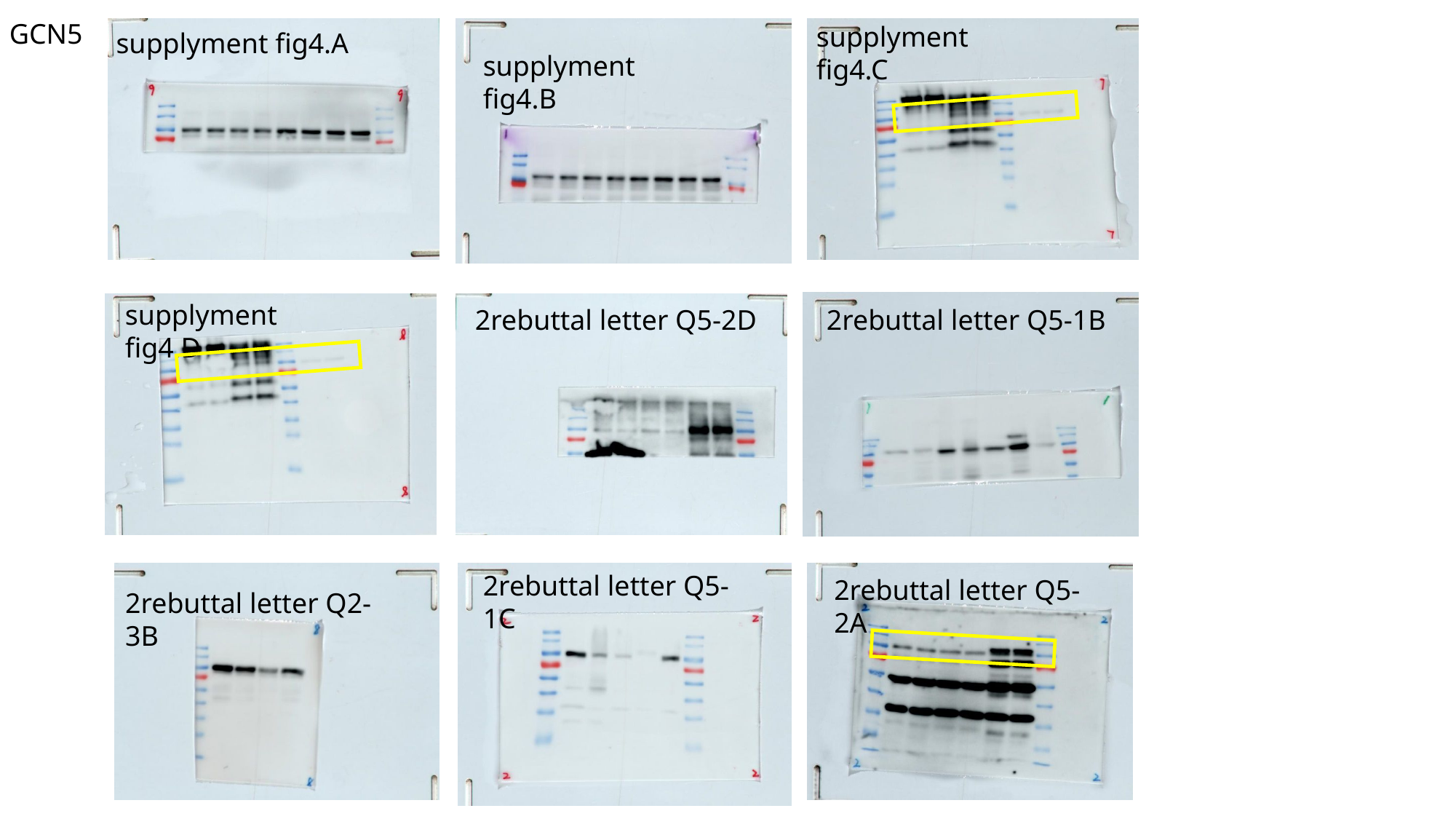

GCN5
supplyment fig4.C
supplyment fig4.A
supplyment fig4.B
supplyment fig4.D
2rebuttal letter Q5-2D
2rebuttal letter Q5-1B
2rebuttal letter Q5-1C
2rebuttal letter Q5-2A
2rebuttal letter Q2-3B

## Slide 5
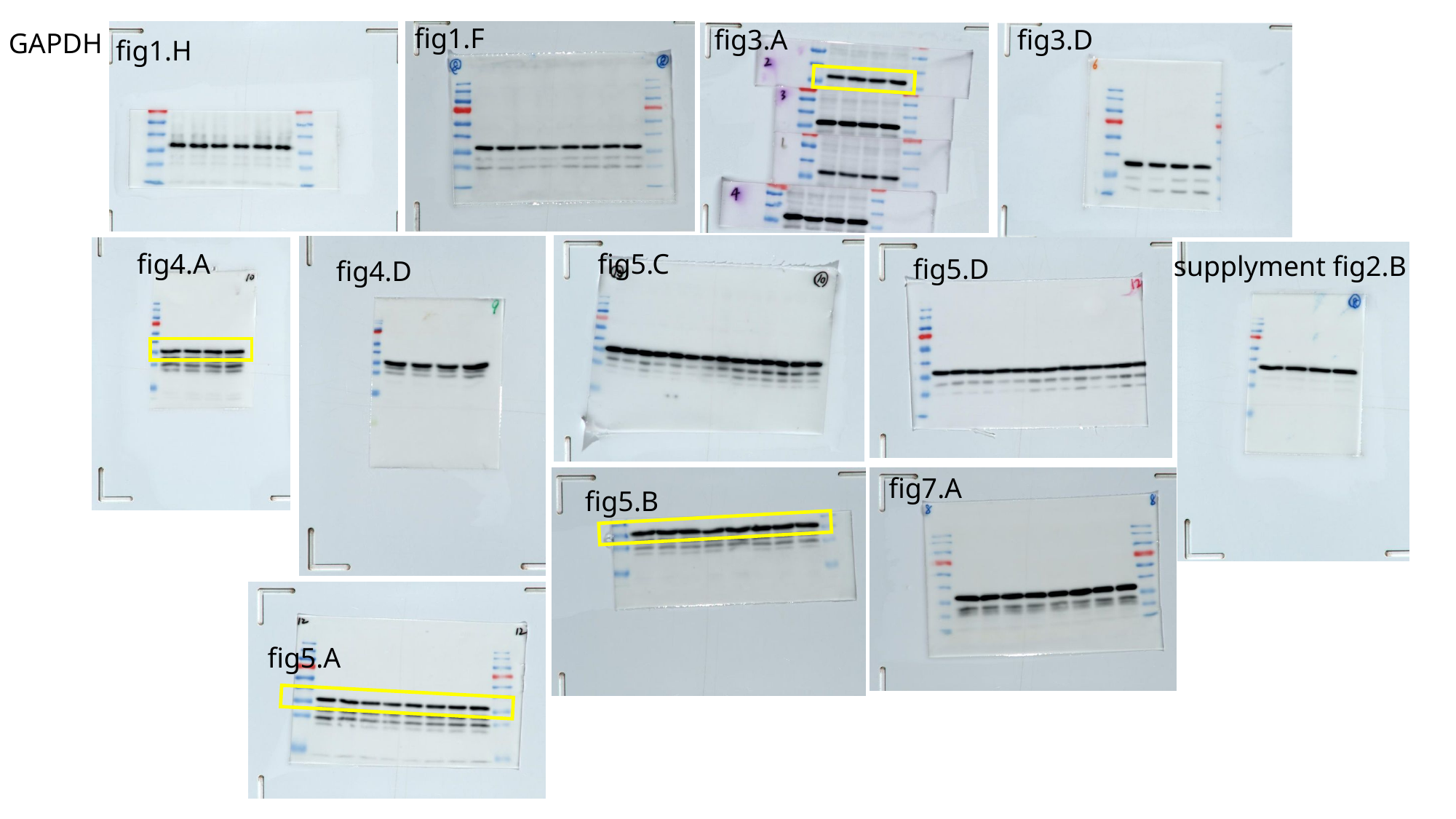

fig1.F
fig3.A
fig3.D
GAPDH
fig1.H
fig4.A
fig5.C
supplyment fig2.B
fig5.D
fig4.D
fig7.A
fig5.B
fig5.A

## Slide 6
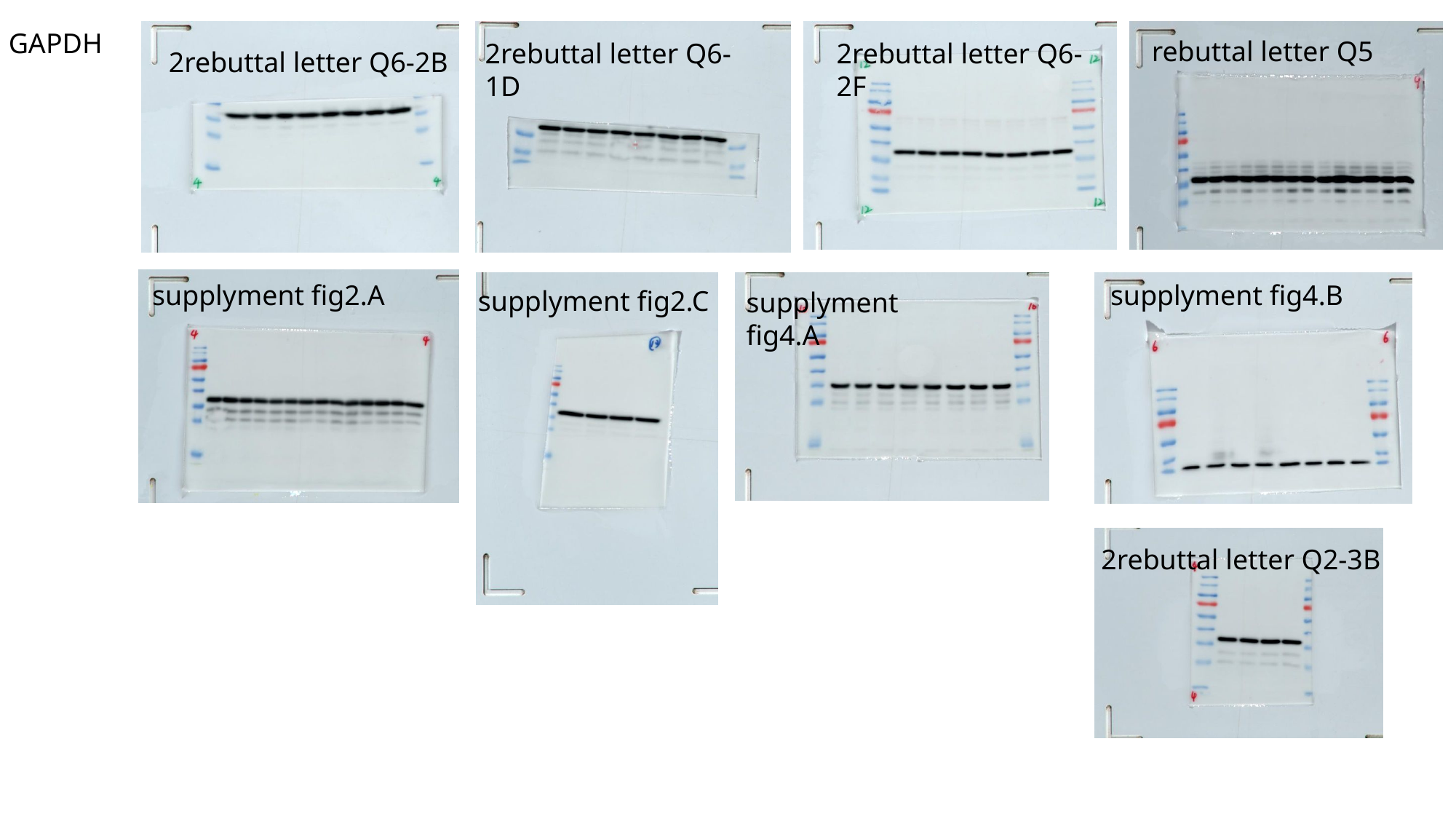

GAPDH
rebuttal letter Q5
2rebuttal letter Q6-1D
2rebuttal letter Q6-2F
2rebuttal letter Q6-2B
supplyment fig2.A
supplyment fig4.B
supplyment fig2.C
supplyment fig4.A
2rebuttal letter Q2-3B

## Slide 7
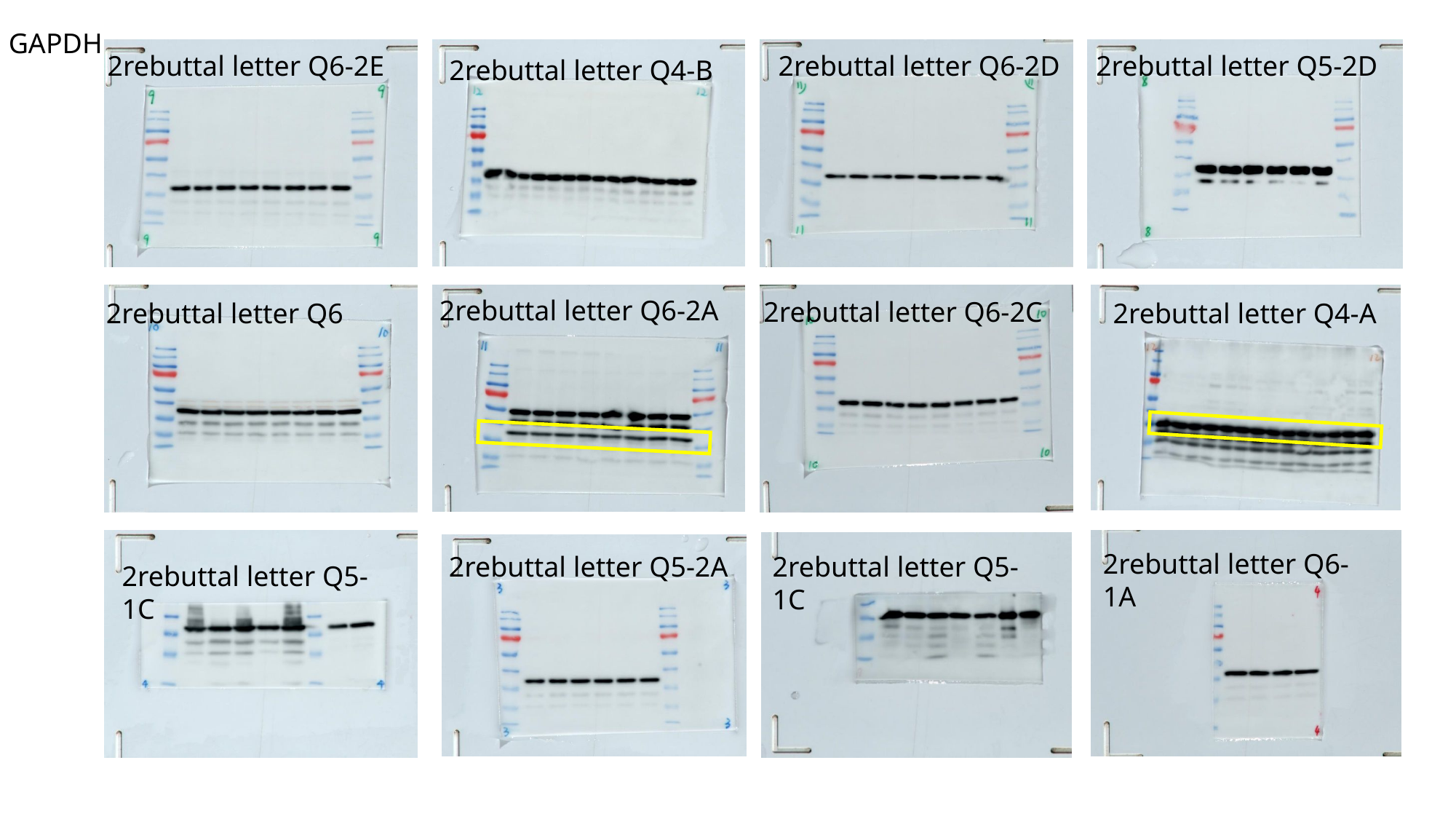

GAPDH
2rebuttal letter Q6-2E
2rebuttal letter Q6-2D
2rebuttal letter Q5-2D
2rebuttal letter Q4-B
2rebuttal letter Q6-2A
2rebuttal letter Q6-2C
2rebuttal letter Q6
2rebuttal letter Q4-A
2rebuttal letter Q6-1A
2rebuttal letter Q5-2A
2rebuttal letter Q5-1C
2rebuttal letter Q5-1C

## Slide 8
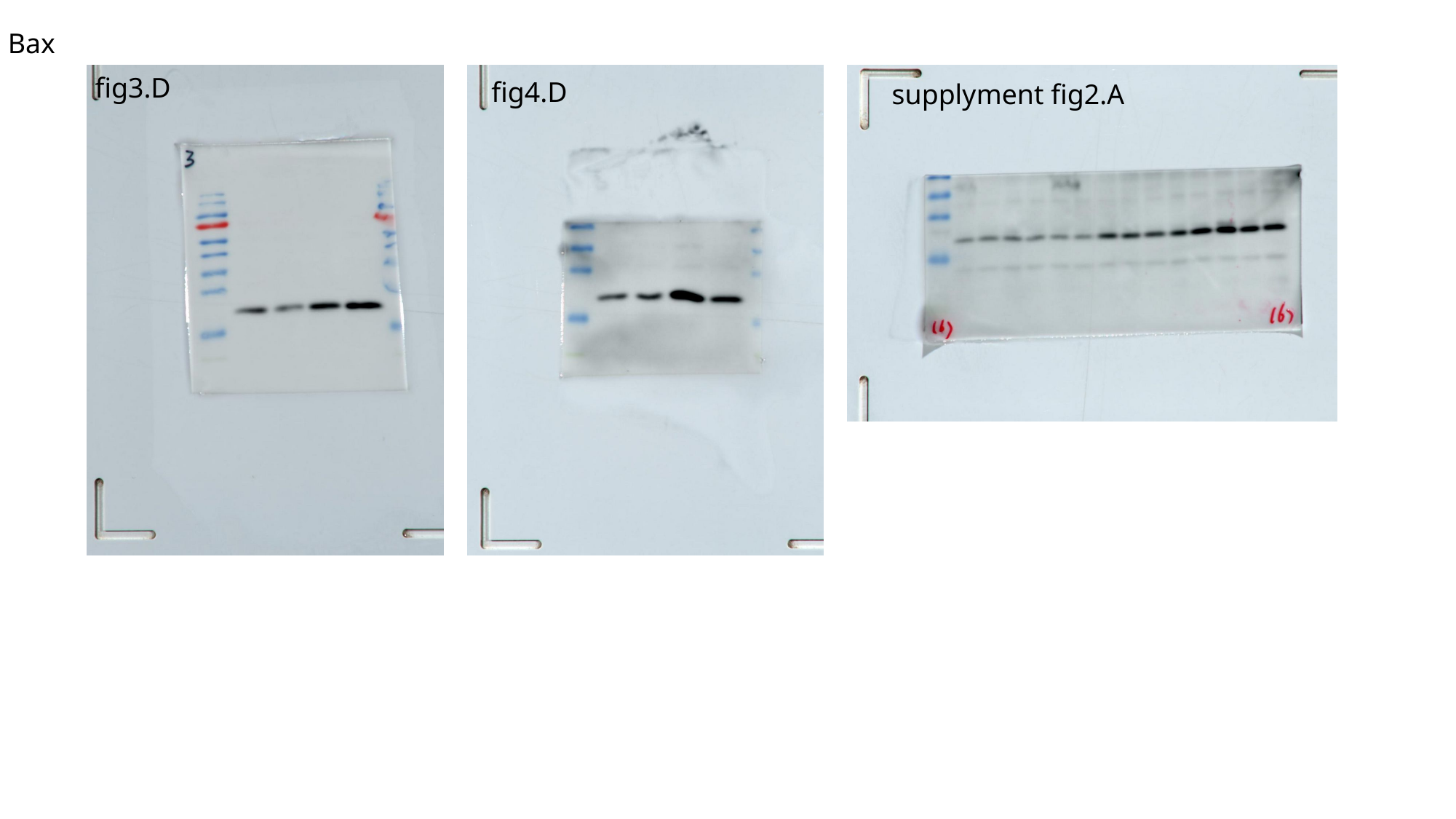

Bax
fig3.D
fig4.D
supplyment fig2.A

## Slide 9
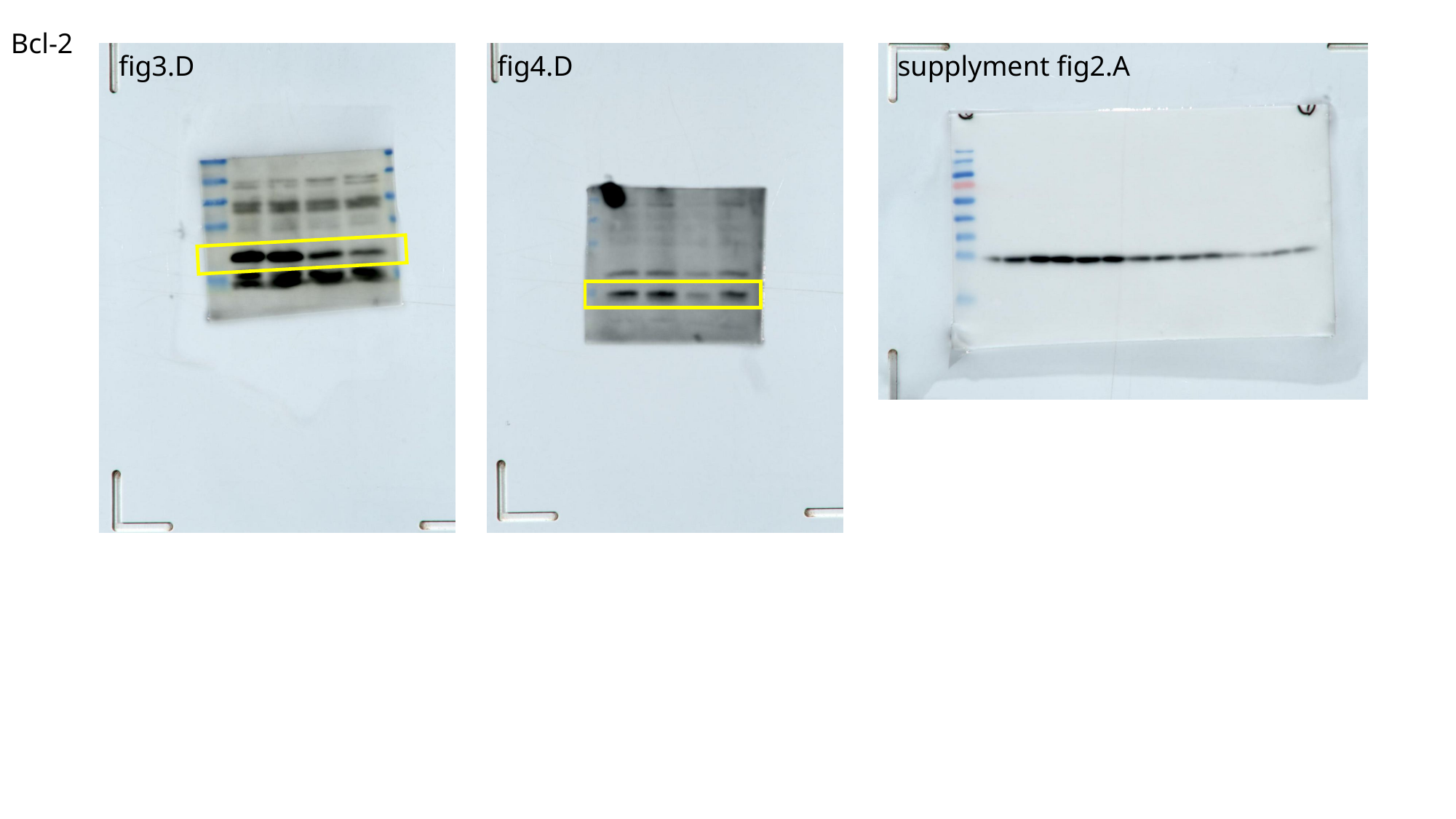

Bcl-2
fig3.D
fig4.D
supplyment fig2.A

## Slide 10
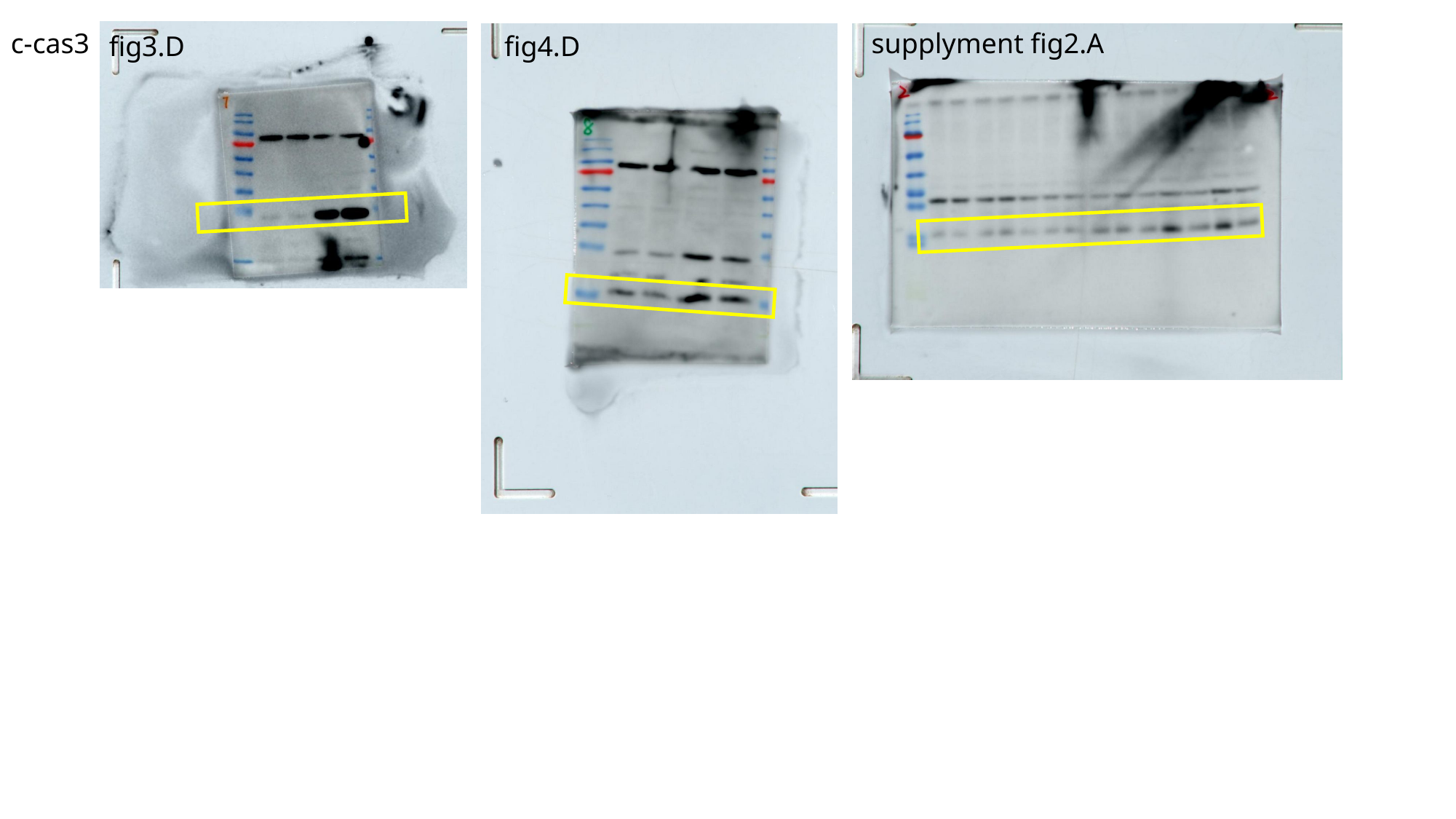

c-cas3
supplyment fig2.A
fig3.D
fig4.D

## Slide 11
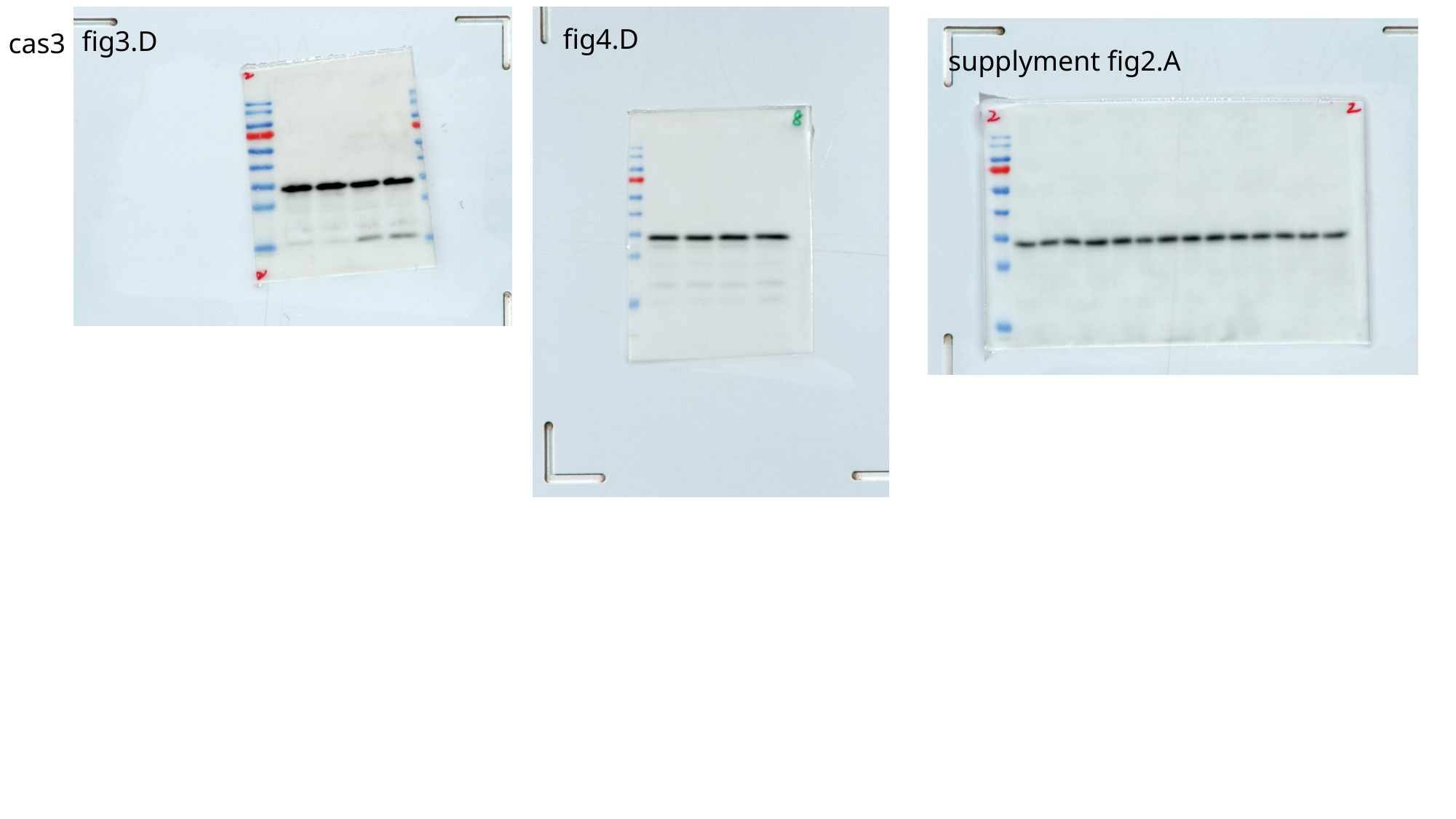

fig4.D
fig3.D
cas3
supplyment fig2.A

## Slide 12
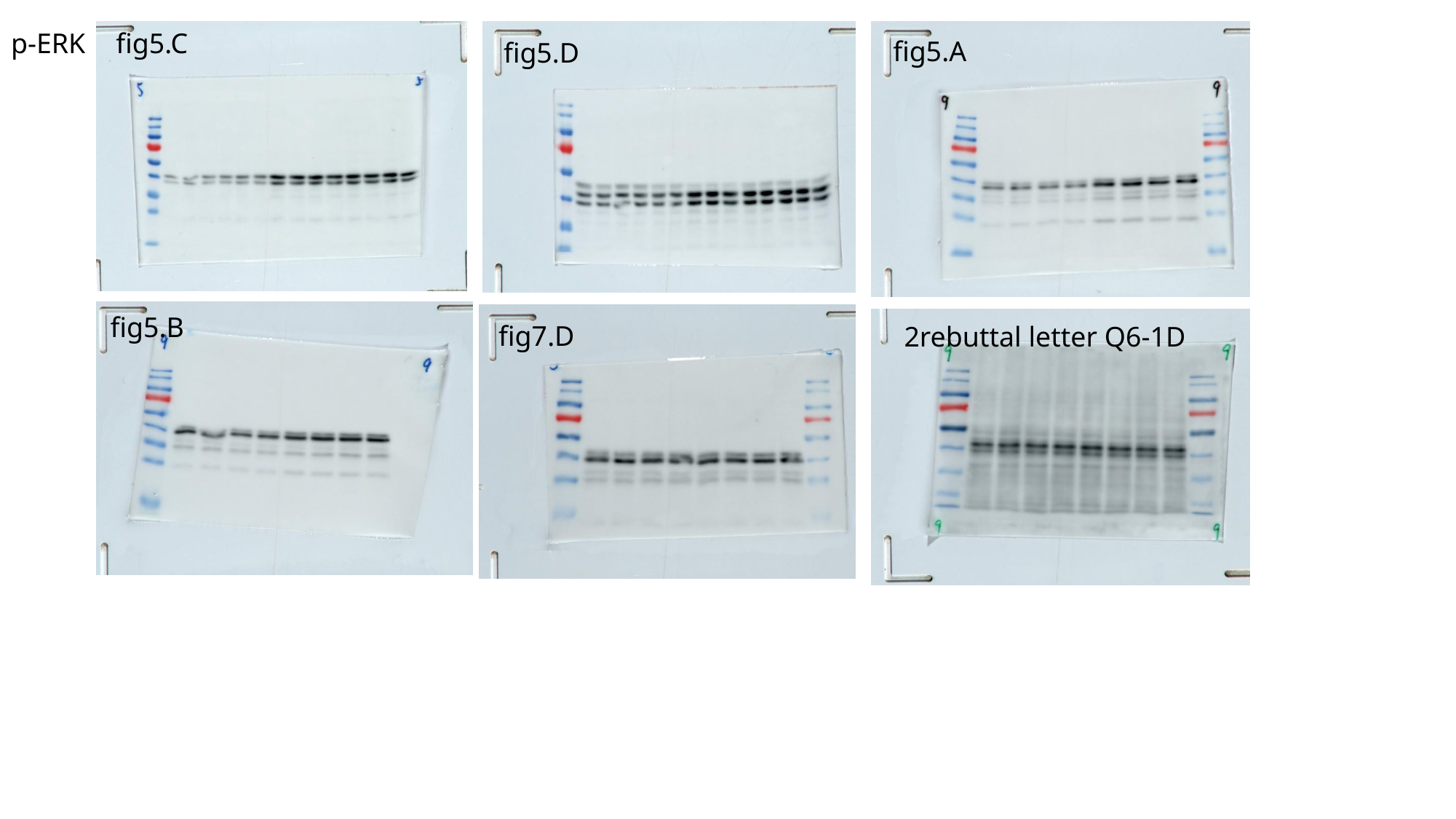

p-ERK
fig5.C
fig5.A
fig5.D
fig5.B
fig7.D
2rebuttal letter Q6-1D

## Slide 13
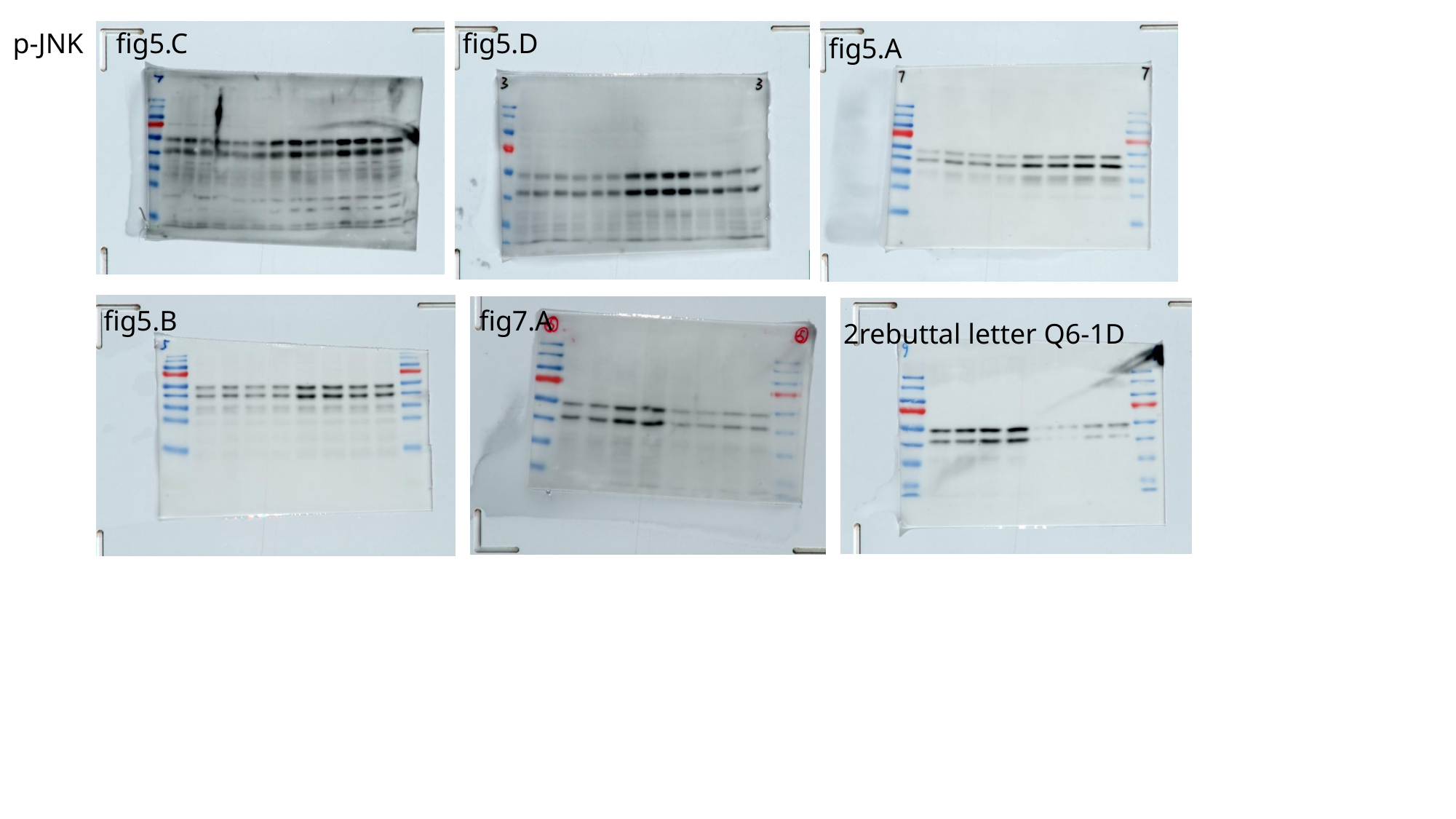

p-JNK
fig5.C
fig5.D
fig5.A
fig5.B
fig7.A
2rebuttal letter Q6-1D

## Slide 14
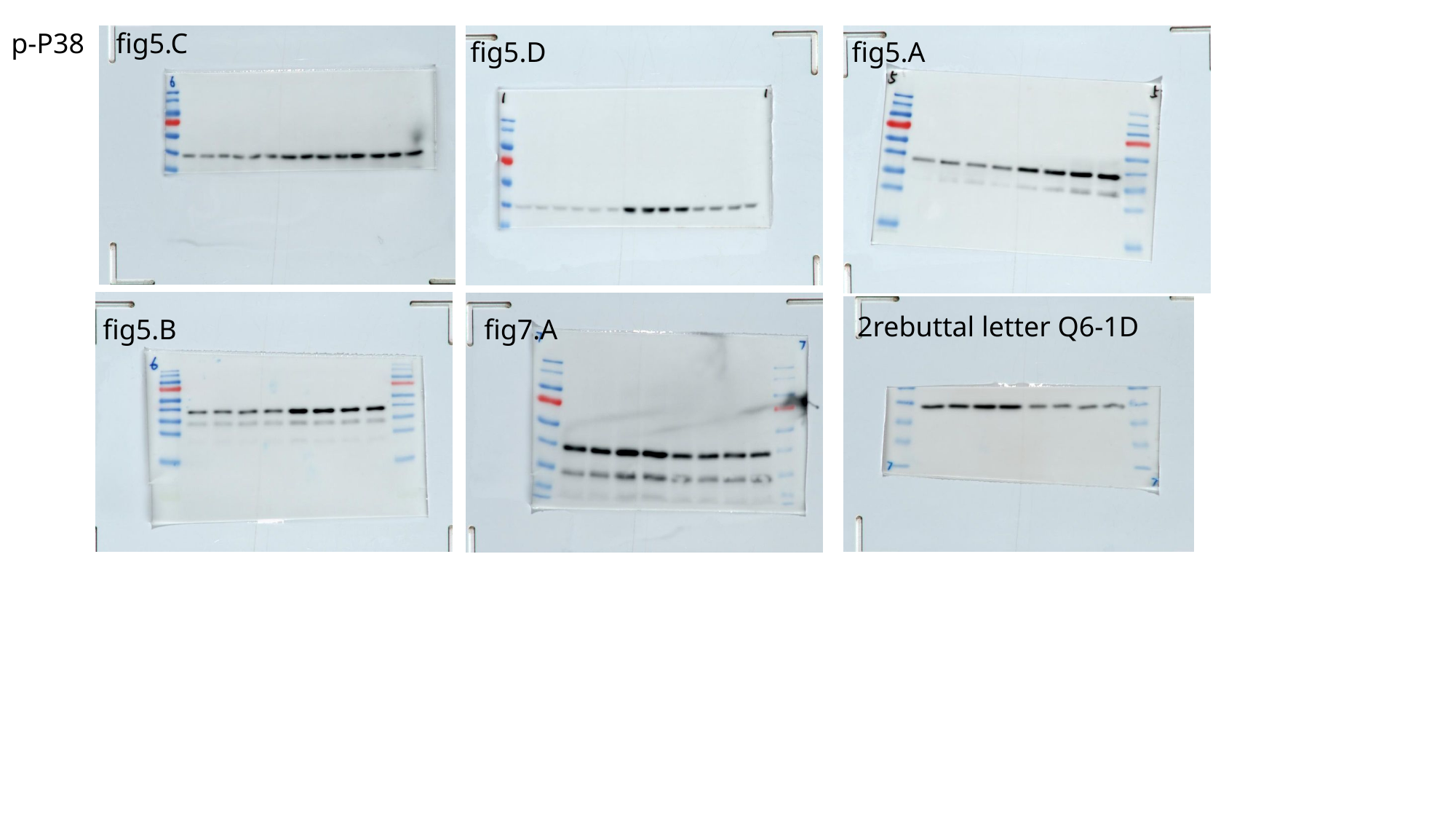

p-P38
fig5.C
fig5.D
fig5.A
2rebuttal letter Q6-1D
fig5.B
fig7.A

## Slide 15
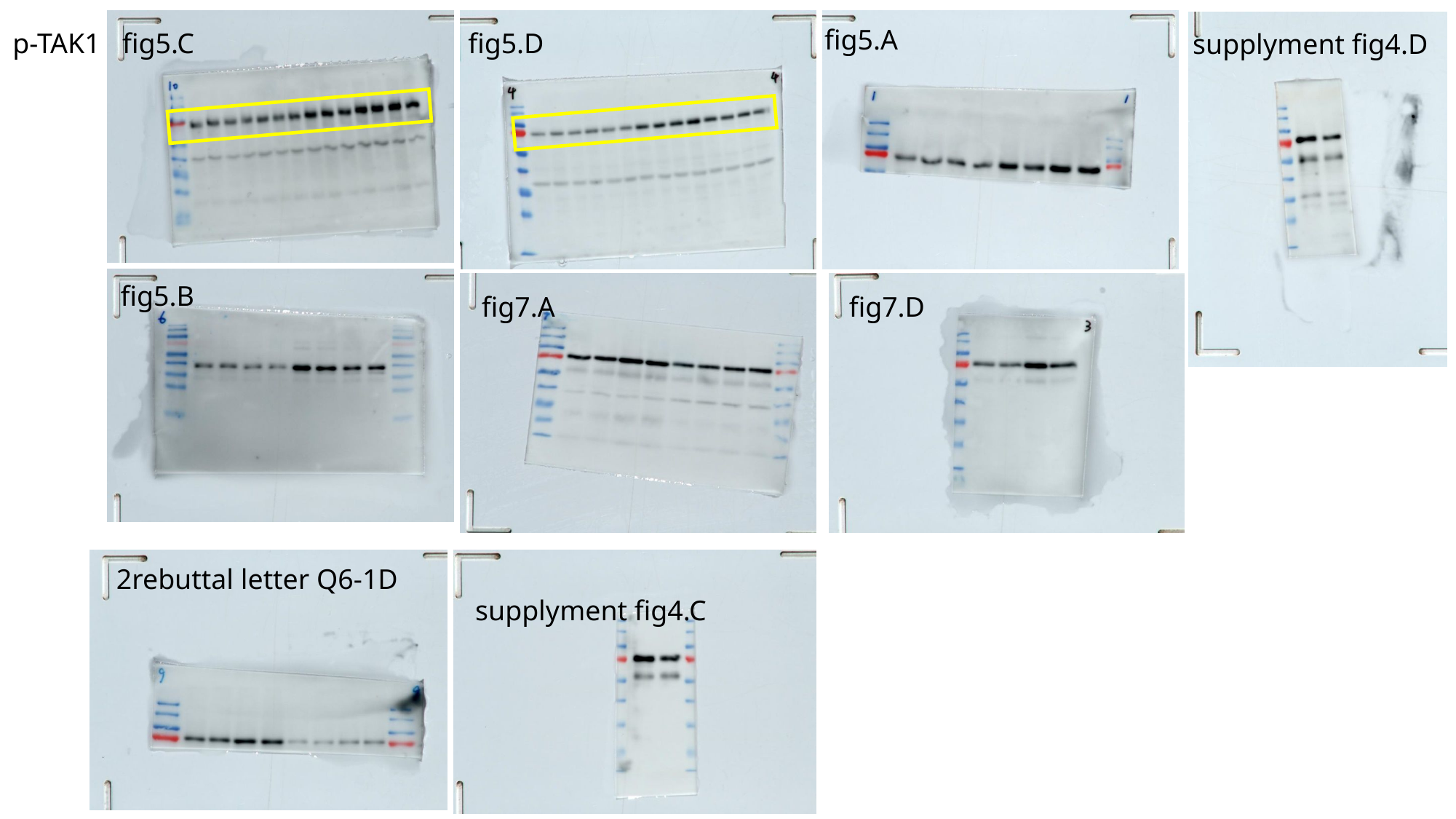

fig5.A
p-TAK1
fig5.C
fig5.D
supplyment fig4.D
fig5.B
fig7.A
fig7.D
2rebuttal letter Q6-1D
supplyment fig4.C

## Slide 16
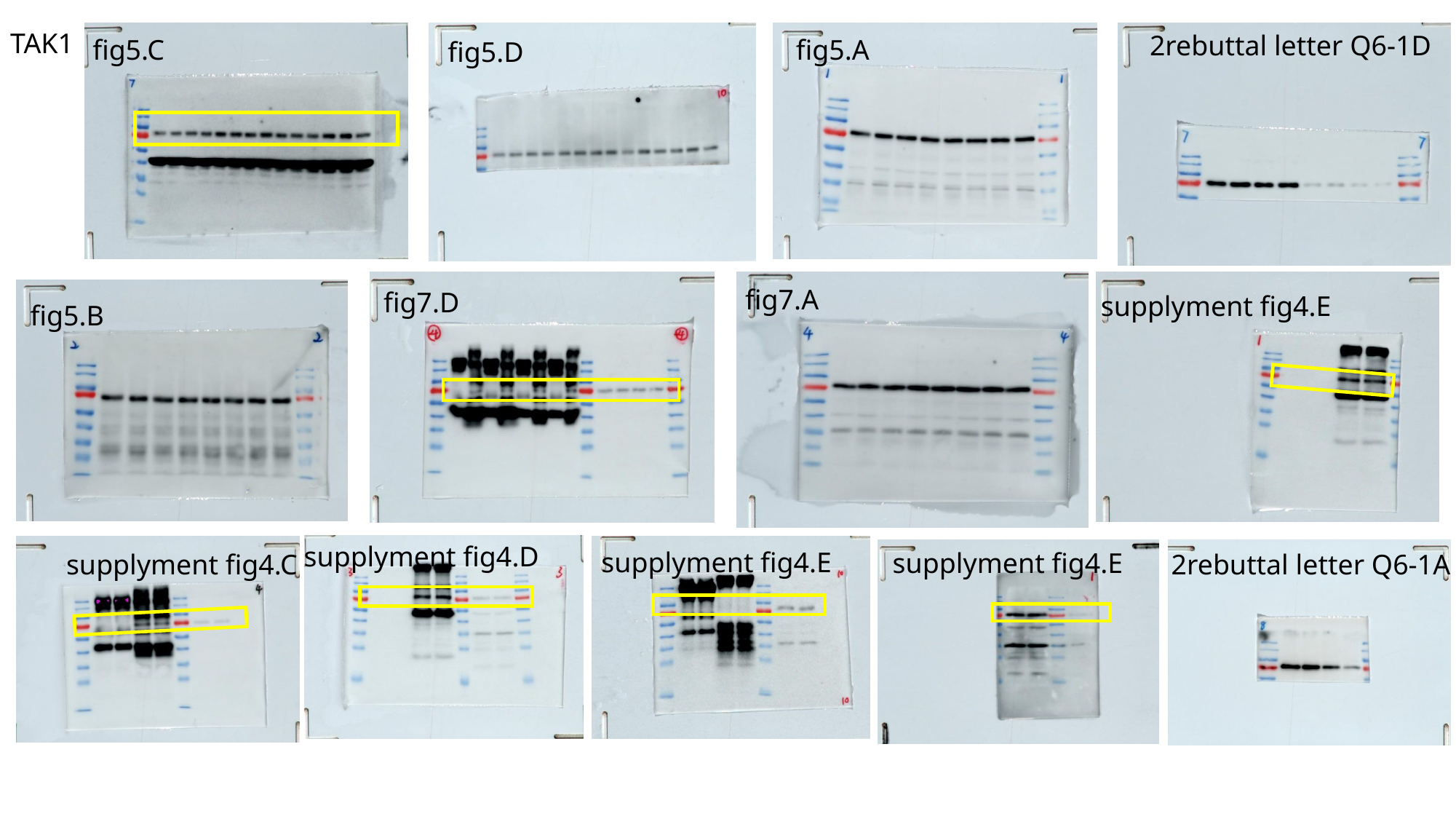

TAK1
2rebuttal letter Q6-1D
fig5.C
fig5.A
fig5.D
fig7.A
fig7.D
supplyment fig4.E
fig5.B
supplyment fig4.D
supplyment fig4.E
supplyment fig4.E
supplyment fig4.C
2rebuttal letter Q6-1A

## Slide 17
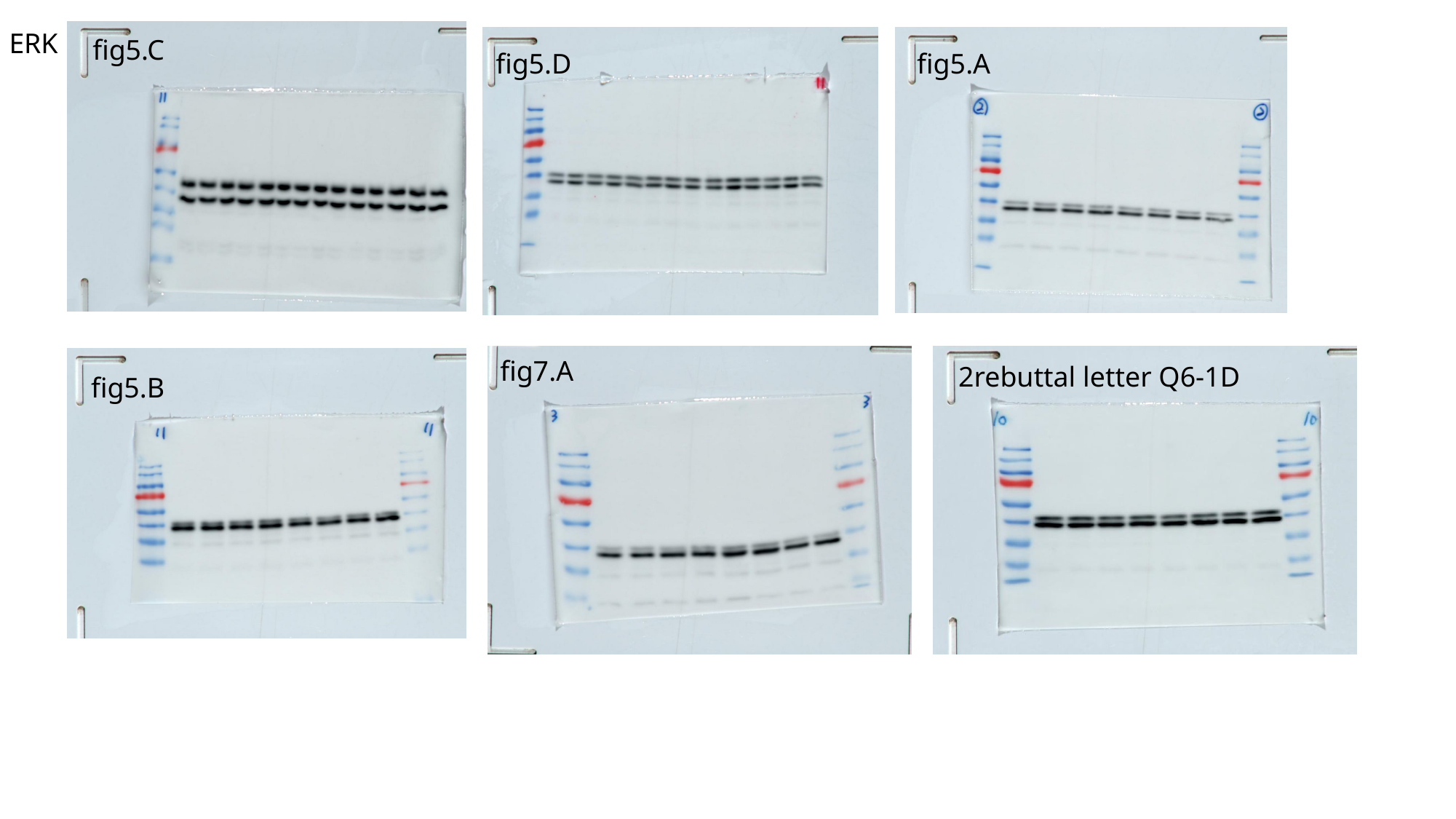

ERK
fig5.C
fig5.D
fig5.A
fig7.A
2rebuttal letter Q6-1D
fig5.B

## Slide 18
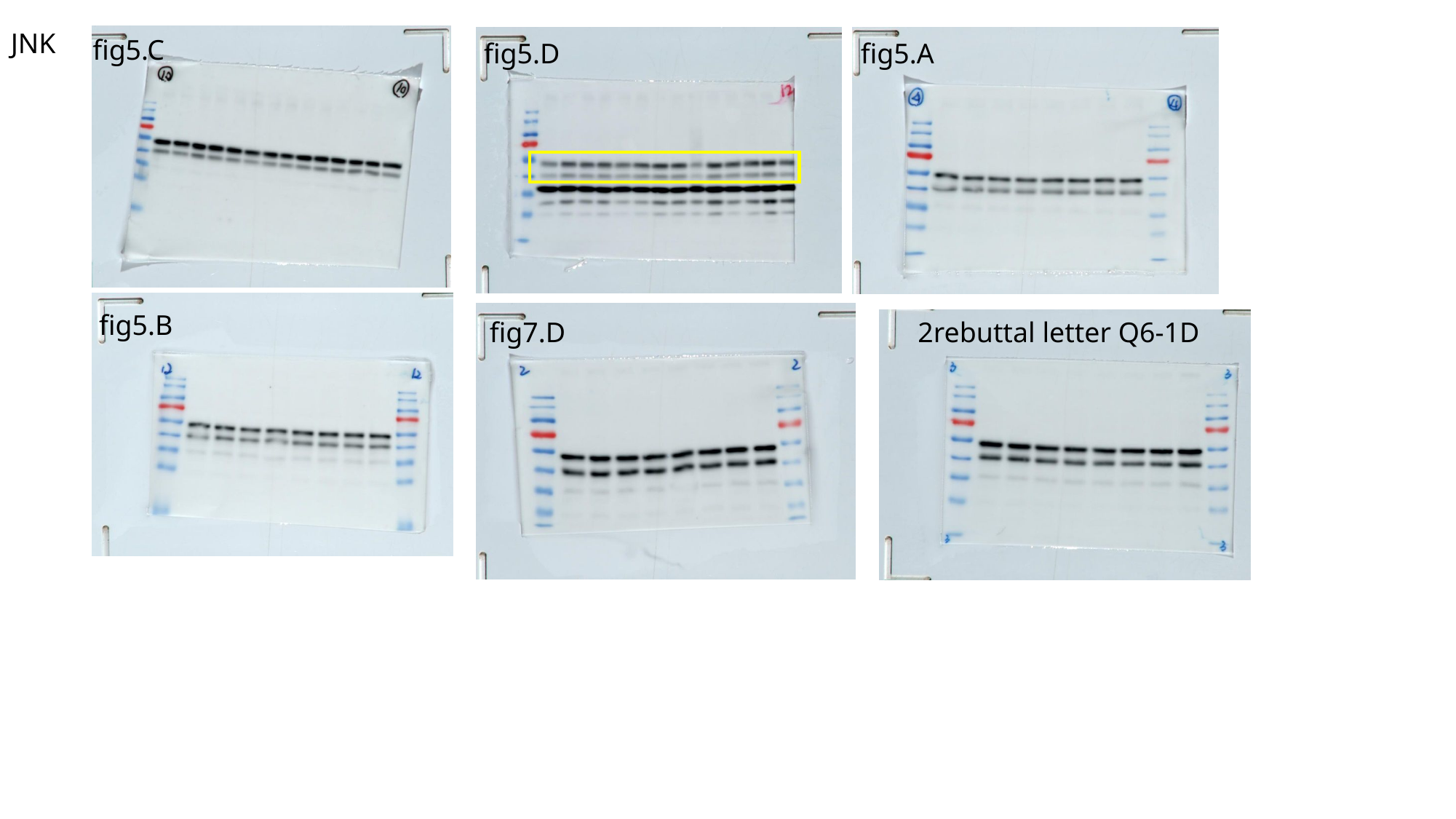

JNK
fig5.C
fig5.D
fig5.A
fig5.B
fig7.D
2rebuttal letter Q6-1D

## Slide 19
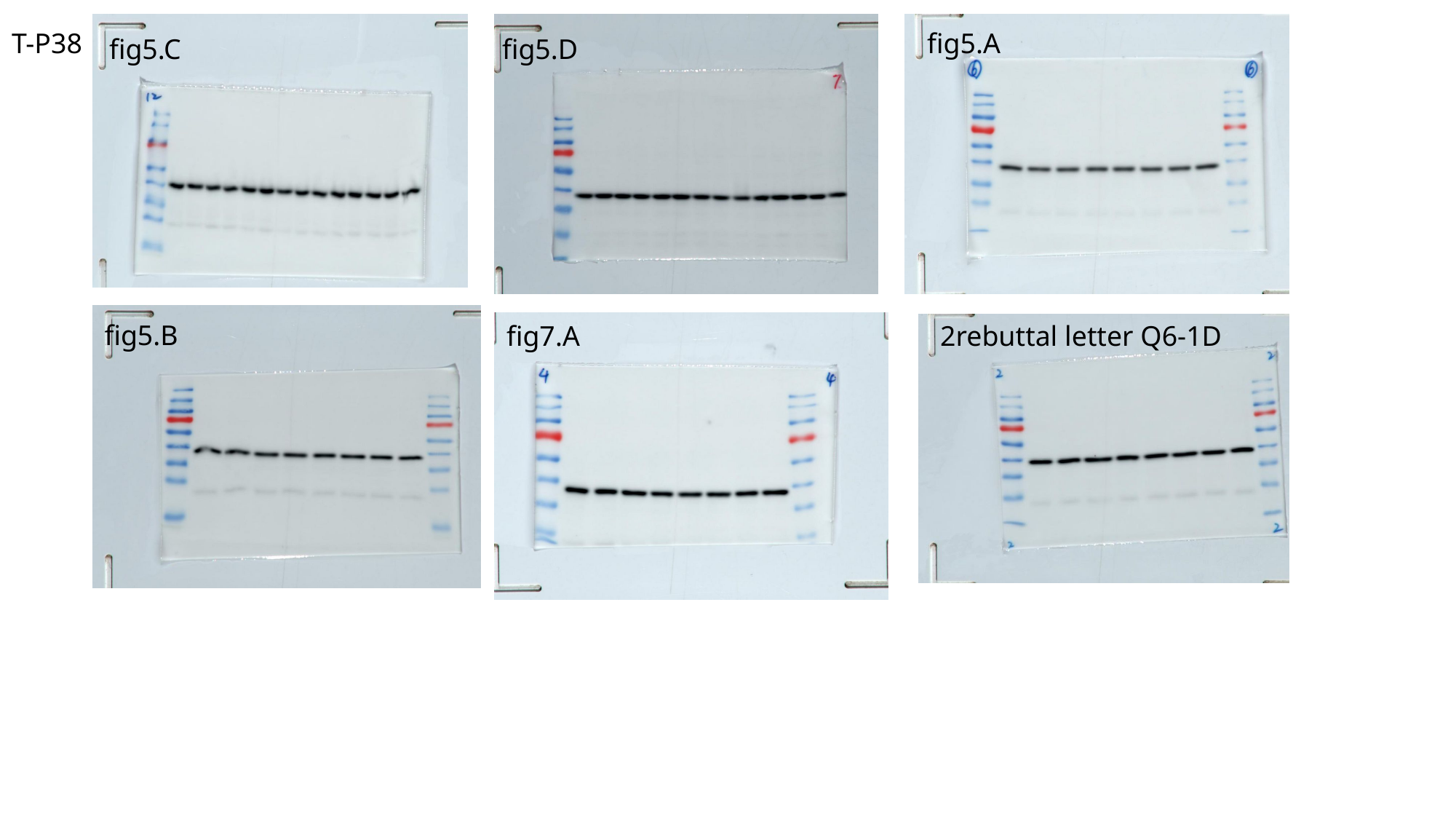

T-P38
fig5.A
fig5.C
fig5.D
fig5.B
fig7.A
2rebuttal letter Q6-1D

## Slide 20
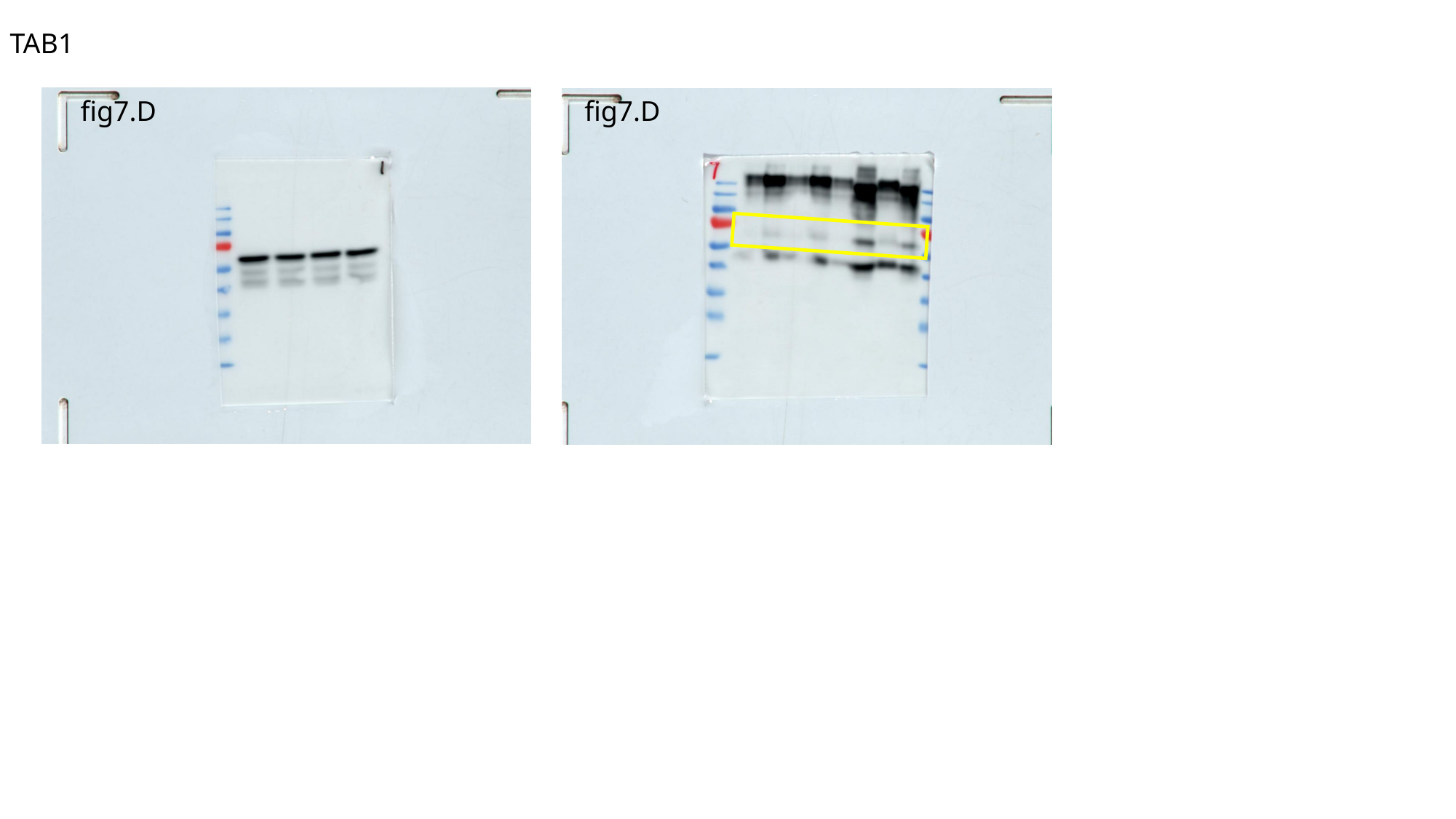

TAB1
fig7.D
fig7.D

## Slide 21
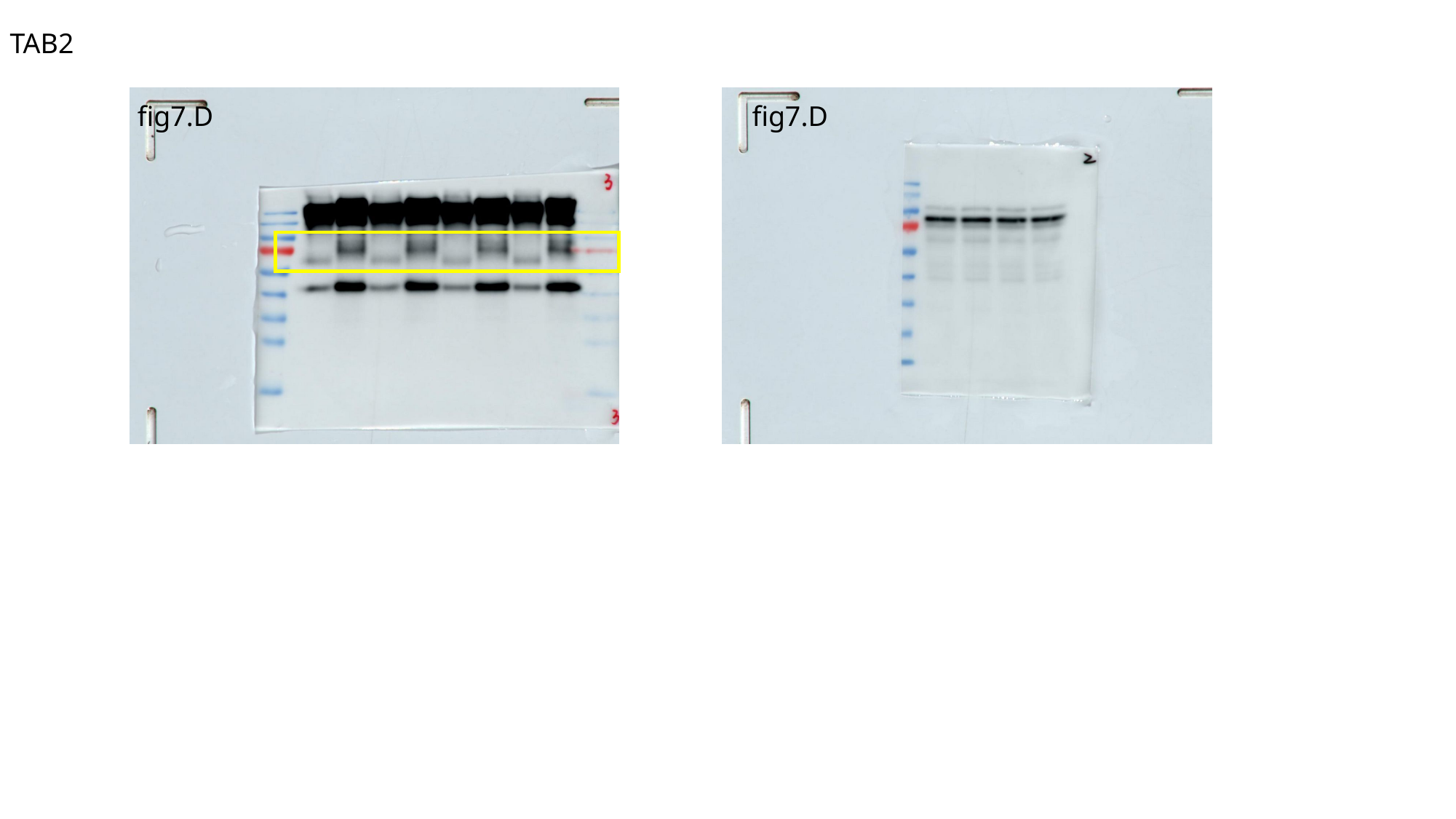

TAB2
fig7.D
fig7.D

## Slide 22
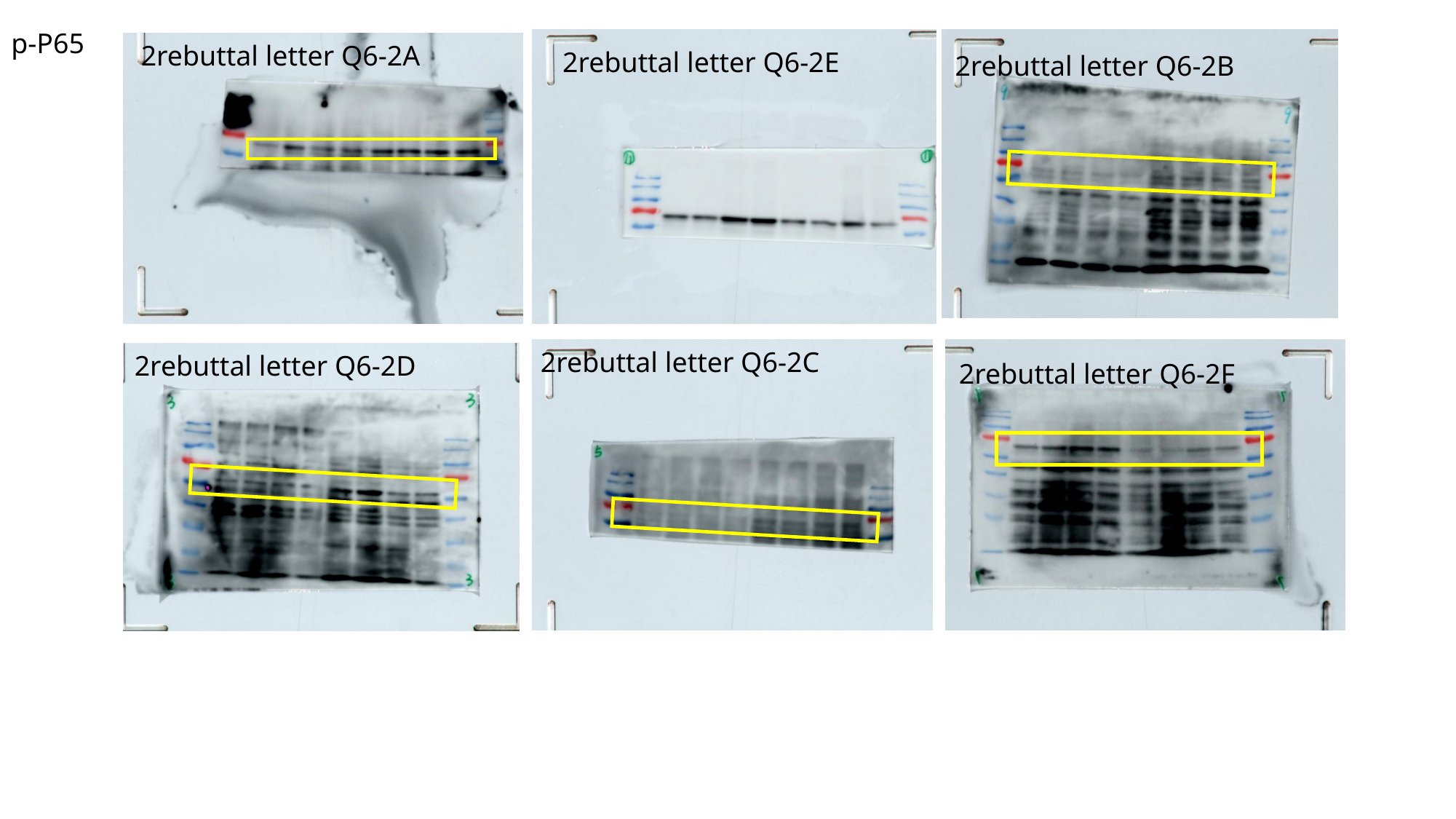

p-P65
2rebuttal letter Q6-2A
2rebuttal letter Q6-2E
2rebuttal letter Q6-2B
2rebuttal letter Q6-2C
2rebuttal letter Q6-2D
2rebuttal letter Q6-2F

## Slide 23
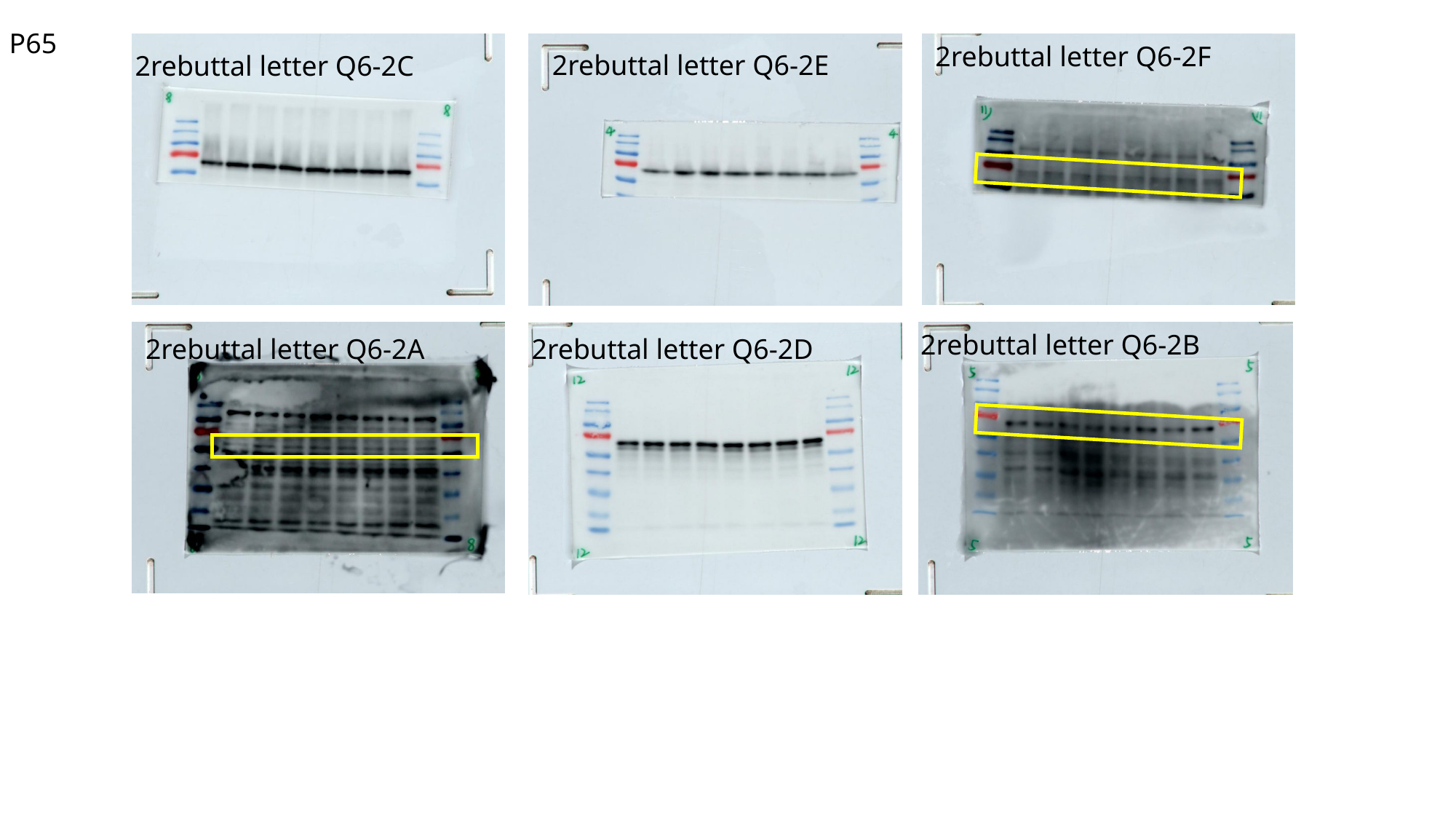

P65
2rebuttal letter Q6-2F
2rebuttal letter Q6-2E
2rebuttal letter Q6-2C
2rebuttal letter Q6-2B
2rebuttal letter Q6-2A
2rebuttal letter Q6-2D

## Slide 24
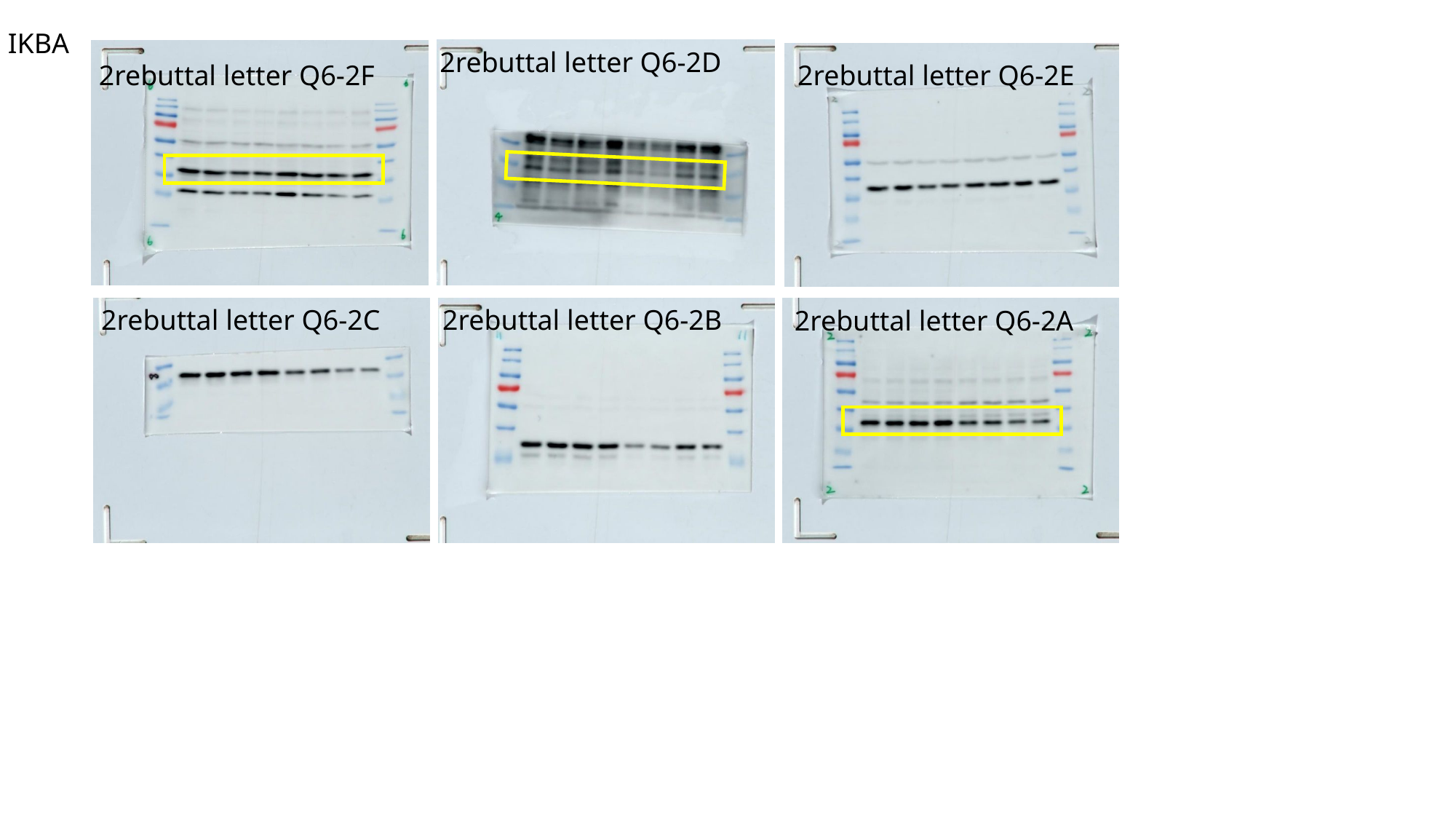

IKBA
2rebuttal letter Q6-2D
2rebuttal letter Q6-2F
2rebuttal letter Q6-2E
2rebuttal letter Q6-2C
2rebuttal letter Q6-2B
2rebuttal letter Q6-2A

## Slide 25
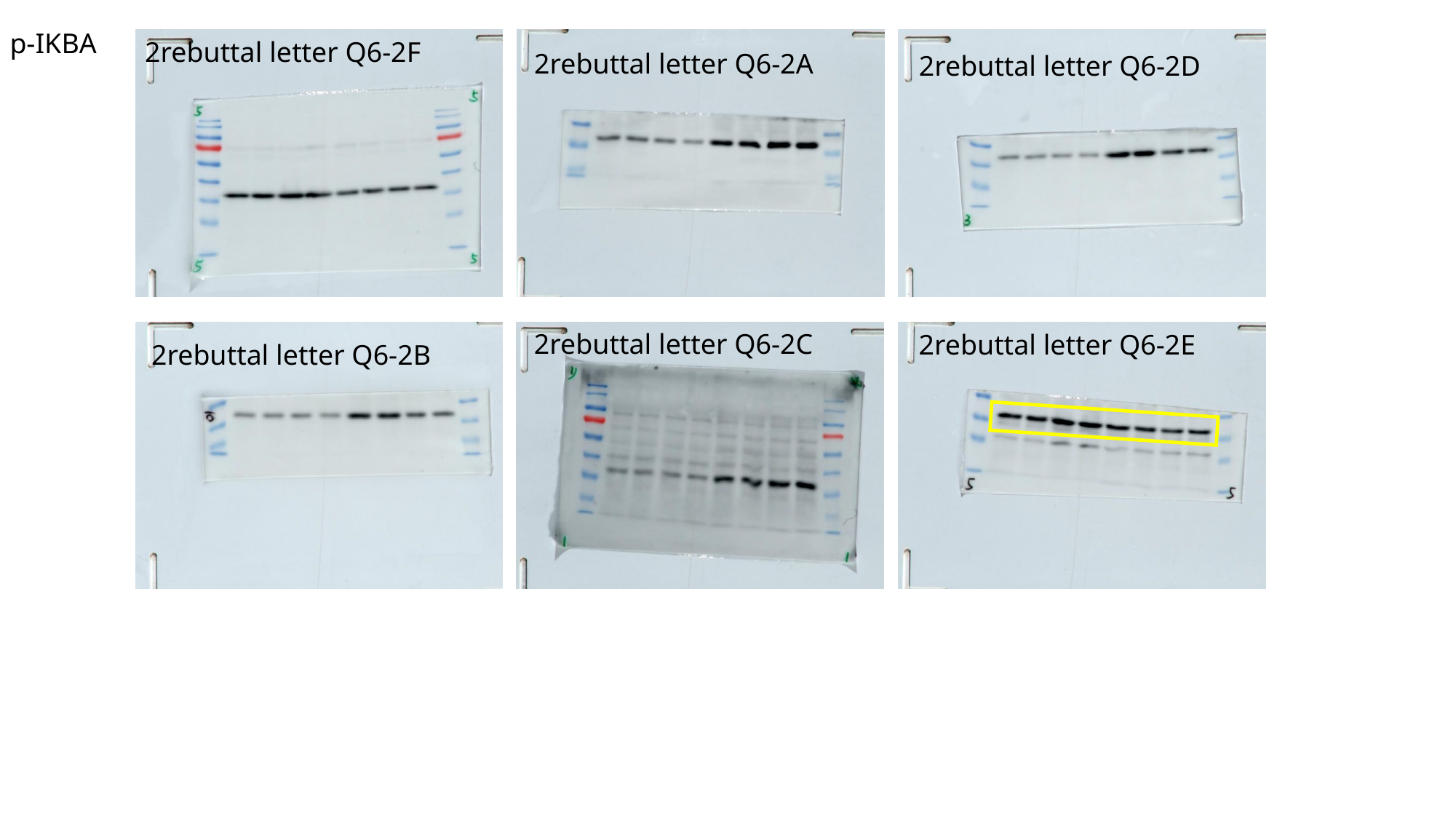

p-IKBA
2rebuttal letter Q6-2F
2rebuttal letter Q6-2A
2rebuttal letter Q6-2D
2rebuttal letter Q6-2C
2rebuttal letter Q6-2E
2rebuttal letter Q6-2B
