# Supplementary material for: GCN5-mediated regulation of pathological cardiac hypertrophy via activation of the TAK1-JNK/p38 signaling pathway
Source: Cell Death Dis. 2022 Apr 30;13(4):421. doi: 10.1038/s41419-022-04881-y (PMC9056507; doi:10.1038/s41419-022-04881-y)
Supplement: Supplementary file 5 — Author Contribution Statement [file 41419_2022_4881_MOESM5_ESM.docx]

**Author Contribution Statement**

J.L. conducted the research, J.L. and D.L. worte the manuscript, C.H.Y. contributed to methodology, Y.L.W and C.C participated in data analysis, K.H. and Y.L.H. designed the research and directed the study. All authors read and approved the final paper.
